# LncRNA FTO-IT1 promotes glycolysis and progression of hepatocellular carcinoma through modulating FTO-mediated N6-methyladenosine modification on GLUT1 and PKM2

**DOI:** 10.1186/s13046-023-02847-2

**Published:** 2023-10-16

**Authors:** Fan Wang, Yuhang Hu, Hongda Wang, Ping Hu, Hewei Xiong, Zhu Zeng, Shengbo Han, Decai Wang, Jie Wang, Yong Zhao, Yan Huang, Wenfeng Zhuo, Guozheng Lv, Gang Zhao

**Affiliations:** Department of Emergency Surgery, Union Hospital, Tongji Medical College, Huazhong University of Science and Technology, Hubei Province, 1277 Jiefang Avenue, Wuhan, 430022 People’s Republic of China

**Keywords:** Hepatocellular carcinoma, Glycolysis, FTO-IT1, m^6^A, FTO

## Abstract

**Background:**

Long non-coding RNAs (LncRNAs) have been extensively studied to play essential roles in tumor progression. However, more in-depth studies are waiting to be solved on how lncRNAs regulate the progression of hepatocellular carcinoma (HCC).

**Methods:**

Different expression levels of lncRNAs in HCC cells were compared by analysis of Gene Expression Omnibus and The Cancer Genome Atlas databases. The effects of lncRNA FTO Intronic Transcript 1 (FTO-IT1) on HCC cells were assessed by gain- and loss-of-function experiments. Colony formation assay, Edu assay, glucose uptake and lactic acid production assay were performed to evaluate the regulation of proliferation and glycolysis of HCC cells by FTO-IT1. The binding between protein interleukin enhancer binding factor 2/3 (ILF2/ILF3) and FTO-IT1 was determined by RNA pull-down, mass spectroscopy and RNA immunoprecipitation experiments. RNA stability assay, quantitative reverse transcription PCR and Western blot were employed to determine the regulatory mechanisms of FTO-IT1 on fat mass and obesity-associated (FTO). Methylated RNA immunoprecipitation assay was used to assessed the regulation of key enzymes of glycolysis by FTO. The role of FTO-IT1/FTO in vivo was confirmed via xenograft tumor model.

**Results:**

LncRNA FTO-IT1, an intronic region transcript of FTO gene, was highly expressed in HCC and associated with poor prognosis of patients with HCC. FTO-IT1 was related to proliferation and glycolysis of HCC cells, and contributed to the malignant progression of HCC by promoting glycolysis. Mechanistically, FTO-IT1 induced stabilization of FTO mRNA by recruiting ILF2/ILF3 protein complex to 3’UTR of FTO mRNA. As a demethylase for *N*^6^-methyladenosine (m^6^A), FTO decreased m^6^A modification on mRNAs of glycolysis associated genes including GLUT1, PKM2, and c-Myc which alleviated the YTH N6-methyladenosine RNA binding protein 2 (YTHDF2)-mediated mRNA degradation. Therefore, the upregulated expression of FTO-IT1 leaded to overexpression of GLUT1, PKM2, and c-Myc by which enhanced glycolysis of HCC. Meanwhile, it was found that c-Myc transcriptional regulated expression of FTO-IT1 by binding to its promoter area under hypo-glucose condition, forming a reciprocal loop between c-Myc and FTO-IT1.

**Conclusions:**

This study identified an important role of the FTO-IT1/FTO axis mediated m^6^A modification of glycolytic genes contributed to glycolysis and tumorigenesis of HCC, and FTO-IT1 might be served as a new therapeutic target for HCC.

**Supplementary Information:**

The online version contains supplementary material available at 10.1186/s13046-023-02847-2.

## Background

Energy metabolism reprogramming plays an important role in the occurrence and development of cancers. One major manifestation of this function is compared with normal differentiated cells that rely primarily on mitochondrial oxidative phosphorylation to produce the energy required for cellular processes, most cancer cells rely on aerobic glycolysis, a phenomenon known as “the Warburg effect” [1]. Glycolysis plays an important role in the development of hepatocellular carcinoma (HCC), including proliferation, invasion and metastasis, immune evasion and drug resistance [2]. For instance, CD36 exerted a stimulatory effect on HCC growth and metastasis, through mediating aerobic glycolysis by the Src/PI3K/AKT/mTOR signaling pathway [3]. Bi et al. demonstrated that HDAC11 regulated glycolysis through the LKB1/AMPK signaling pathway to maintain HCC stemness, progression, and sorafenib resistance [4]. Furthermore, a previous study has shown that a glycolysis-related gene signature could predict survival outcomes in patients with HCC, while high risk scores were associated with adverse survival outcomes [5]. Although these researches indicate a pivotal role of glycolysis of HCC progression, the precise mechanisms for the enhanced level of glycolysis have not been fully elucidated.

Long non-coding RNAs (lncRNAs) are the largest of the non-coding RNA subtypes which defined as transcripts of longer than 200 nucleotides that are not translated into proteins [6]. Numerous studies show that lncRNAs are dysregulated in multiple cancers and affects cancer progression by regulating glycolysis. Study showed that lncRNA EPB41L4A-AS1 regulated glycolysis and glutaminolysis by mediating nucleolar translocation of HDAC2 [7]. LncRNA NEAT1 promoted the proliferation and metastasis of breast cancer by accelerating glycolysis [8]. Liu et al. demonstrated that lncRNA AGPG bound to and stabilized PFKFB3, which subsequently activated glycolytic flux and promoted cell cycle progression [9]. However, the accurate mechanism for lncRNA involved in glycolysis of HCC needs to be further investigated.

In our study, we integrated Gene Expression Omnibus (GEO) and The Cancer Genome Atlas (TCGA) databases to reveal that FTO Intronic Transcript 1 (FTO-IT1) was a glycolysis associated lncRNA, which was upregulated and associated with the poor prognosis of HCC. Meanwhile, expression of FTO-IT1 in HCC cells was manipulated to observe the function of FTO-IT1 on proliferation and glycolysis. Moreover, RNA immunoprecipitation (RIP) and RNA pull-down were performed to explore the regulatory mechanism on its target fat mass and obesity-associated (FTO). Furthermore, the methylated RNA immunoprecipitation (MeRIP) and RNA pull-down were conducted to reveal the m^6^A modification of FTO on glycolysis associated genes. Nevertheless, the expression of FTO-IT1 in HCC was detected under various conditions including hypoxia, acidosis, hypo-glucose, and hypo-glutamine to explore the regulation of metabolic microenvironment on FTO-IT1 expression.

## Methods

### Clinical samples

We obtained 92 pairs of HCC tissue samples, and corresponding adjacent nontumorous tissue sample, along with related clinical information from patients undergoing hepatectomy at the Department of Hepatology Surgery in Union Hospital (Wuhan, China). Histopathological diagnosis was implemented according to the National Comprehensive Cancer Network (NCCN) guidelines by two pathologists. Part of the excised tissue specimens were fixed in 10% buffered formalin solution and then embedded in paraffin, and part of the samples were immediately frozen at liquid nitrogen after surgical resection.

### Glycolytic activity assay

Cellular glycolytic activity was measured by glucose uptake and lactate production. For glucose uptake assay, briefly, cells were seeded in 6-well plates and cultured for 24 h. Then, cells were treated with 2-NBDG (50 μmol/L; APExBIO, Houston, USA) for 1 h, followed by Fluorescence Activated Cell Sorting (FACS) analysis using BD LSRFortessa™ X-20 Flow Cytometer (BD Biosciences, Franklin Lakes, USA). Cellular glucose uptake was quantified using fluorescence intensity. For lactate production assay, cells were seeded in 6-well plates and cultured for 24 h. Then, cell culture medium was taken out, and lactate concentration in the media was determined using Lactic Acid Assay Kit (Nanjing Jiancheng Bioengineering Institute, Nanjing, China). Lactate production was expressed as lactate concentration per 1 × 10^4^ viable cells. Meanwhile, the pH of the media was measured by INESA REX PHSJ 4F pH meter (REX, Shanghai, China).

### Biotin-RNA pull-down assay and mass spectrometry

Total RNA was extracted from the cells using TRIzol (TaKaRa, Beijing, China) and was reverse transcribed and amplified by qPCR using primers containing a T7 promoter sequence-specific for FTO-IT1, and truncates or antisense of FTO-IT1, as well as 3’UTR of FTO mRNA or antisense of 3’UTR of FTO mRNA. After purification, the amplified product was transcribed into indicated RNA in vitro using the Transcript Aid T7 High Yield Transcription Kit (Thermo Fisher Scientific, Waltham, USA). Then biotin-labeled RNAs were synthesized by Pierce™ RNA 3’ End Desthiobiotinylation Kit (Thermo Fisher Scientific). Next, cell lysates were prepared using standard lysis buffers in Pierce™ Magnetic RNA–Protein Pull-Down Kit (Thermo Fisher Scientific). Added biotin-labeled RNAs with streptavidin magnetic beads bound into cell lysates to completed binding of RNA-Binding Proteins to RNA. Finally, the RNA-binding protein complexes were washed and eluted, and the protein products were analyzed by Western blot or mass spectrometry. Deletion-mapping analyses were conducted with specific constructed plasmids in a similar way as described above.

Mass spectrometry analysis included biotinylated FTO-IT1 pull-down group and FTO-IT1-AS pull-down group. Firstly, trypsin (mass ratio 1:50) (Promega, Madison, USA) was added to the reduced and alkylated protein samples, and the samples were enzymolyzed at 37 ℃ for 20 h. Secondly, loaded the enzymolysis products to Q Exactive mass spectrometer combined with EASY-nLC 1000 system (Thermo Fisher Scientific) the mass spectrometer for mass spectrometry analysis, and collect the corresponding mass spectrometry data. Finally, by searching the database (uniprot_Homo_sapiens_171145_20190515.fasta) with Proteome Discoverer 1.4 software, the raw files of the mass spectrometry were identified as the final protein results. Searching parameters were as follows: trypsin, up to 2 missed cleavage; carbamidomethyl (C) as fixed modification; oxidation (M) as fixed modification; filter by peptide confidence = High. Primer sequences of aforementioned RNAs for transcription in vitro are given in Supplementary Table S1.

### RNA immunoprecipitation (RIP)

RNA immunoprecipitation (RIP) was performed with antibodies specific to ILF2, ILF3, YTHDF2, or IgG by using Magna RIP™ RNA-Binding Protein Immunoprecipitation Kit (Millipore, Boston, USA) according to the manufacture’s protocol. Firstly, cell lysates were prepared using cell lysis buffer containing protease inhibitors and RNase inhibitors. Subsequently, magnetic beads were pre-incubated with antibodies or IgG for 30 min at room temperature and washed by RIP wash buffer. The lysates were then immunoprecipitated with the bead-bound antibodies at 4 °C overnight in RIP immunoprecipitation buffer included with 0.5 M EDTA and 5 μL RNase inhibitor. Magnetic beads were reserved and washed repeatedly after centrifugation. To purify the RNA bound with protein, proteinase K buffer was used to re-suspend magnetic beads. Supernatant was removed, and the immunoprecipitate was mixed with salt solution I, salt solution II, precipitate enhancer, and absolute ethanol overnight at -80 ℃. Finally, the samples were centrifuged at 14 000 rpm for 15 min at 4 ℃, and washed with 80% ethanol. Pellets were re-suspended in 10 to 20 μL of RNase-free water for analysis by qPCR.

### Methylated RNA immunoprecipitation (MeRIP)

m^6^A modifications of RNAs were measured by methylated RNA immunoprecipitation (MeRIP) assay. Total RNA was extracted from cells by using Trizol RNAiso Plus (TaKaRa), and purified using Spin Column RNA Cleanup & Concentration Kit (Sangon Biotech, Shanghai, China). Then, purified total RNA was treat using VAHTS mRNA capture beads to obtain poly-A-purified RNA (Vazyme, Nanjing, China). Next, intact poly-A-purified RNA was denatured at 70 °C for 10 min, transferred immediately on ice. Then RNAs were incubated with m^6^A antibody (Abcam, Cambridge, UK) in 1 mL buffer containing RNase inhibitor (Beyotime, Shanghai, China), 50 mmol/L Tris–HCl, 750 mmol/L NaCl, 0.5% (vol/vol) Igepal CA-630 and 200 mmol/L RVC (Sangon Biotech) for 2 h at 4°C. Protein G (Bimake, Shanghai, China) was washed, added to the mixture, and incubated for 2 h at 4 °C with rotation. The captured RNA was eluted twice with 6.7 mmol/L *N*^6^-methyladenosine 5’-monophosphate sodium salt at 4 °C for 1 h and precipitated with 5 μg glycogen, one-tenth volumes of 3 M sodium acetate in 2.5 volumes of 100% ethanol at -80 °C overnight. m^6^A enrichment was determined by qPCR analysis.

The global m^6^A levels in mRNA was measured with EpiQuik m^6^A RNA Methylation Quantification Kit (Colorimetric) (Epigentek, Farmingdale, USA) following the manufacturer’s protocol. Briefly, total RNA was isolated, and then added into reaction wells. Assay strip plates were covered with parafilm and incubated at 37 ℃ for 90 min. Next, capture antibody was added into reaction wells to capture RNA. After 60 min of incubation, the reaction wells were washed with washing buffer and enhancing solution, respectively. Finally, stop solution and developer solution were used to detected signal of reaction. The absorbance was read on a microplate reader at 450 nm. The relative m^6^A quantification was compared by absorbances.

### Animal experiments

Male BALB/c nude mice (aged 4–6 weeks; *n* = 5/group) were obtained from Vital River Laboratory Animal Technology (Beijing, China). MHCC97H or Huh7 cells (2 × 10^6^ cells/mouse) stably transfected with lentivirus containing different plasmids in 100 μL DMEM were subcutaneously or orthotopically implanted into the nude mice. Tumor volumes were measured every 4 days according to the formula V = 0.5 × L (length) × W^2^ (width). Mice were sacrificed after 4 weeks. Tumors were isolated and weighed. Then the tumor xenografts were embedded in paraffin, followed by staining with H&E and immunohistochemistry (IHC).

### Statistical analysis

All results were presented as means ± standard deviation (SD) by GraphPad Prism 8.0 (GraphPad Software, San Diego, USA). Student’s *t* tests were used to analyze the difference between two groups. The one-way ANOVA test was used for multiple comparisons. Pearson’s correlation analysis and *x*^2^ test were used to analyze the correlation between different two genes. Kaplan–Meier analysis with log-rank tests were used to compare the difference in survival rates of groups. Arithmetic mean was used to divide high group and low group of expression of RNA or protein. All statistical results were two-sided. An estimate of variation was performed within each group of data. The data meet the assumptions of the tests, respectively. The *P* value was indicated in the figure, and *P* < 0.05 was considered to indicate statistical significance. N.S. meant that the difference was not significant.

### Supplementary methods

Details of cell culture, RNA isolation, reverse transcription, and quantitative real-time PCR (qRT-PCR), transfection, cell proliferation assay, Cell migration and invasion assays, Cell apoptosis assay, RNA fluorescence in situ hybridization (RNA-FISH), Western blot, RNA stability assay, Co-immunoprecipitation (Co-IP), Chromatin immunoprecipitation (ChIP), luciferase activity assay and immunohistochemistry (IHC) were available in the Supplementary Methods section. The sequences of siRNAs were shown in Supplementary Table S2. The details of antibodies of above experiments were listed in Supplementary Table S3.

## Results

### FTO-IT1 was upregulated in HCC samples and might function as a tumor-promoting lncRNA

**Fig. 1:**
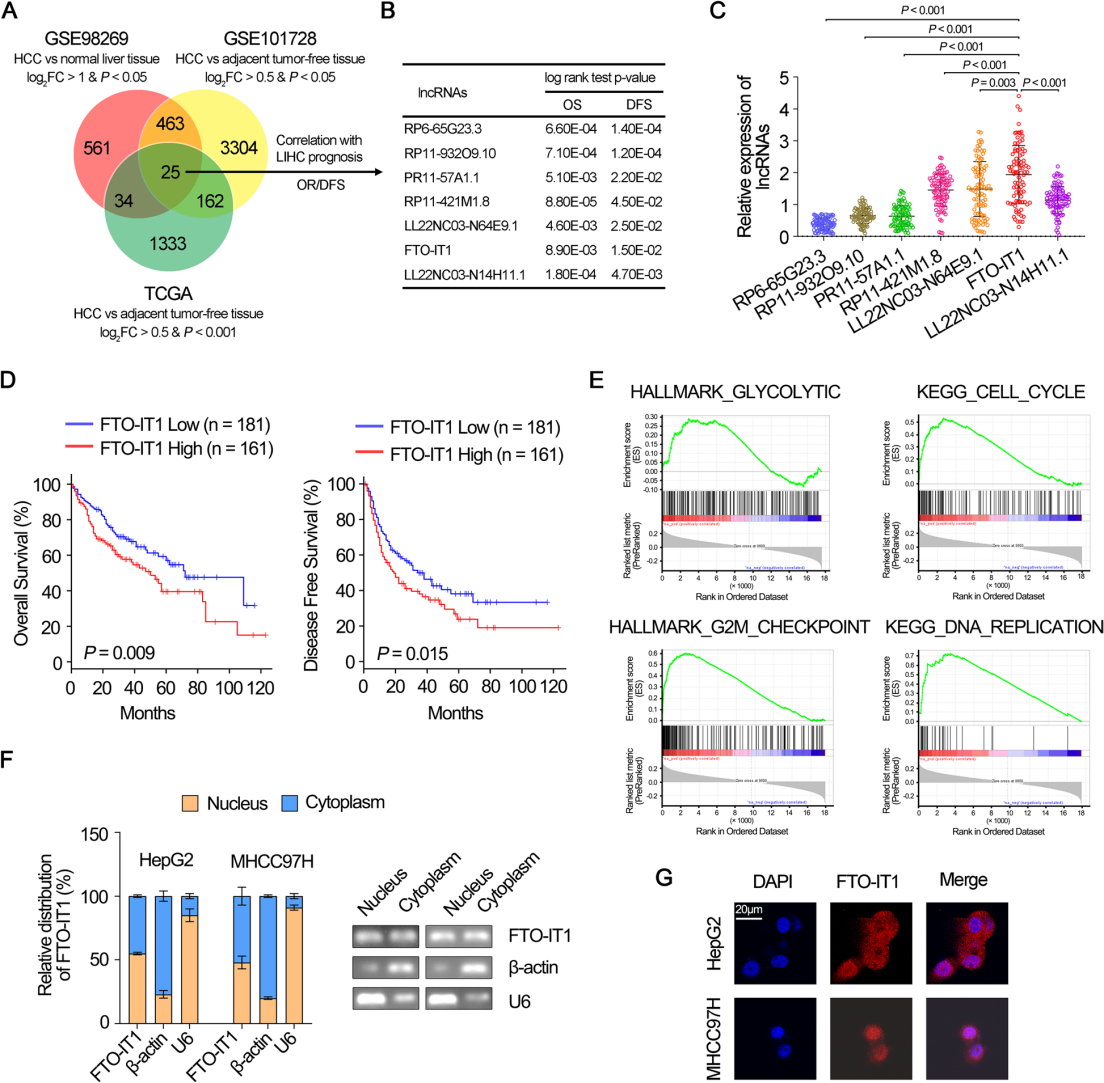
FTO-IT1 was upregulated in HCC samples and might function as a tumor-promoting lncRNA. **A** Venn diagram indicating the identification of differentially expressed lncRNAs among GSE98269, GSE101728 and TCGA datasets. **B** Log-rank test of OS and DFS of the 7 lncRNAs which screening from the aforementioned three datasets. **C** The relative expression of the 7 lncRNAs were detected in 92 HCC clinical specimens by qRT-PCR. **D** Kaplan–Meier survival analysis using online tool GEPIA (http://gepia.cancer-pku.cn) showed the OS and DFS of FTO-IT1. **E** Gene set enrichment analysis of FTO-IT1-correlated genes in TCGA database. NES, normalized enrichment score. **F** qRT-PCR detected the subcellular fractions of FTO-IT1 in HepG2 and MHCC97H cells (left). The qRT-PCR products were separated by 2% agarose gel electrophoresis; U6 and β-actin were used as markers of the nucleus and cytoplasm, respectively (right). **G** RNA-FISH analysis of FTO-IT1 (red) in HepG2 and MHCC97H cells. Scale bars, 20 μm

To identify lncRNAs which contribute to HCC tumorigenesis and progression, we first analyzed the microarray dataset of HCC derived from NCBI GEO series (GSE98269 and GSE101728) and TCGA database. We screened 25 lncRNAs which were simultaneously upregulated in the three datasets by comparing the differential expression between HCC tissues and normal hepatic tissues (Fig. 1A). Subsequently, Kaplan–Meier survival analysis was performed by employing the online tool GEPIA. We found that the high expression of 7 lncRNAs was associated with poor overall survival (OS) and disease-free survival (DFS) in patients with HCC (Fig. 1B). We next quantified these lncRNAs in 92 HCC clinical specimens by qRT-PCR analysis, and lncRNA FTO-IT1 was found to be most upregulated among these lncRNAs (Fig. 1C). The survival curve based on TCGA data also revealed that the OS and DFS of HCC patients with high expression of FTO-IT1 were significantly reduced (Fig. 1D). Meanwhile, analysis from Gene Set Enrichment Analysis (GSEA), Gene ontology (GO), and Kyoto Encyclopedia of Genes and Genomes (KEGG) displayed that FTO-IT1 was associated with glycolysis, cell cycle, and DNA replication (Fig. 1E; Supplementary Fig. S1A and B). The nuclear and cytoplasm fractions of HepG2 and MHCC97H cells were isolated, and the PCR results displayed the FTO-IT1 signal was detected in both nucleus and cytoplasm (Fig. 1F). Furthermore, RNA-FISH analysis further demonstrated FTO-IT1 was evenly distributed in nucleus and cytoplasm (Fig. 1G). The sequence of the full-length FTO-IT1 and its secondary structure based on online tool were shown respectively (Supplementary Fig. S1C and D). In addition, the Coding Potential Calculator 2 (CPC2) and PyhloCSF indicated that FTO-IT1 has very weak protein-coding potential (Supplementary Fig. S1E and F). Together, these results suggested that FTO-IT1 was highly expressed in HCC and associated with poor prognosis of HCC, which indicated that FTO-IT1 might function as an oncogenic promoter in HCC.

### FTO-IT1 promoted glycolysis and proliferation of HCC cells

**Fig. 2:**
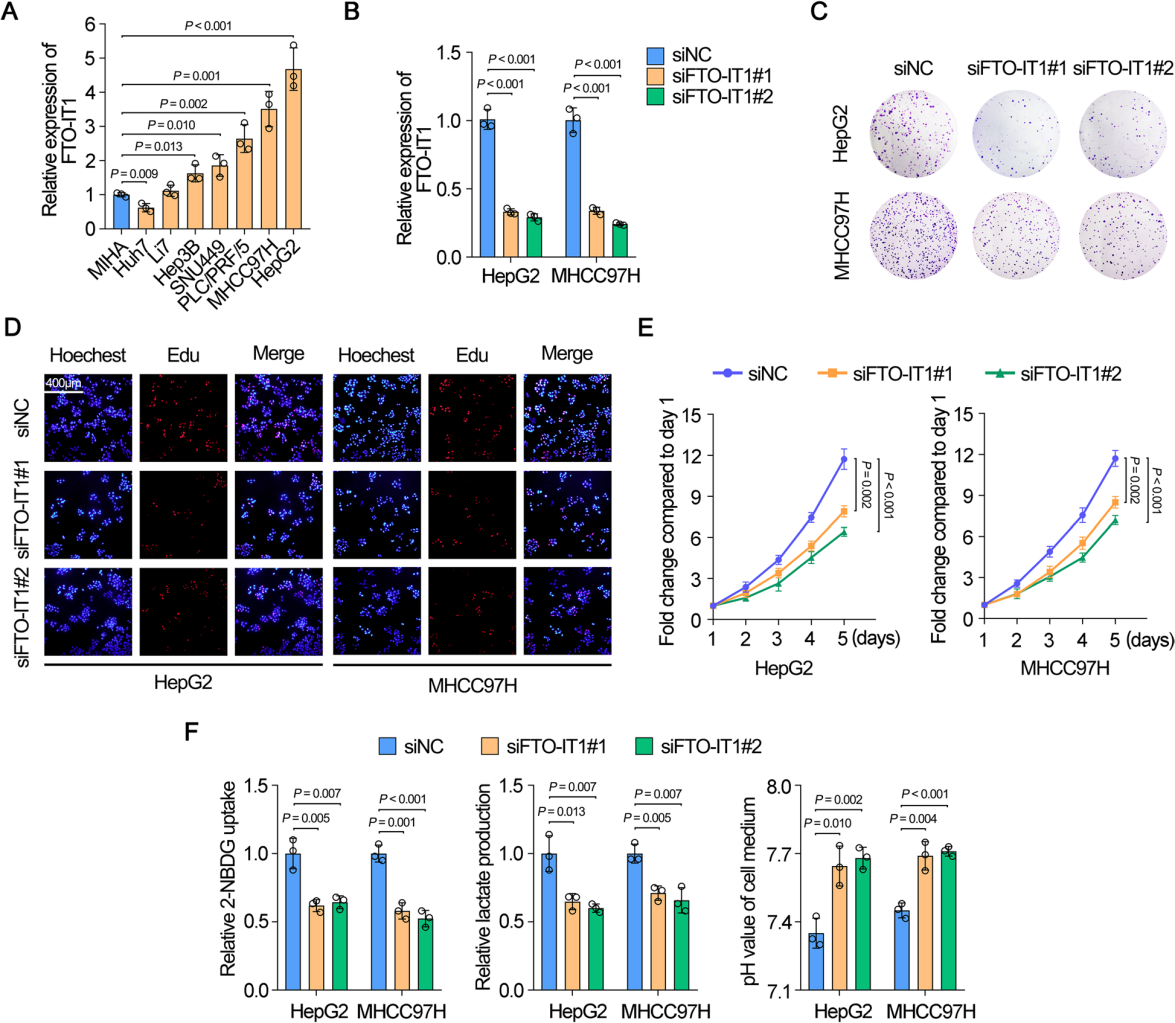
FTO-IT1 promoted glycolysis and proliferation of HCC cells. **A** qRT-PCR showed the relative levels of FTO-IT1 in normal liver cell line (MIHA) and HCC cell lines. **B** Knockdown efficiency of two siRNAs of FTO-IT1 was assessed via transfection assay followed by qRT-PCR. **C**-**E** Colony formation assays (**C**), EdU assays (**D**) and MTT assays (**E**) were used to evaluate the proliferation ability of HepG2/MHCC97H cells transfected with siFTO-IT1 or siNC. **F** The glucose uptake (left), lactate production (middle) and pH value (right) of HCC cells transfected with siNC or siFTO-IT1

To further investigate the functions of FTO-IT1 on HCC, we first quantified the relative expression levels of FTO-IT1 in seven HCC cell lines and normal liver cell line (MIHA). The results showed that FTO-IT1 was up-expression in most HCC cell lines, with the higher expression both in HepG2 and MHCC97H cells and the lowest expression in Huh7 cells (Fig. 2A). After knockdown of FTO-IT1 (Fig. 2B), the capacity of colony formation and proliferation in HepG2 and MHCC97H cells was significantly impaired (Fig. 2C-E).

Since bioinformatic analysis reminded an interaction between FTO-IT1 and glycolysis, we further investigated the role of FTO-IT1 in glycolysis of HCC. Results showed that the knockdown of FTO-IT1 obviously inhibited glucose uptake and lactate production, as well as increased the pH value in corresponding medium, which indicating an impaired capacity of glycolysis in HepG2 and MHCC97H (Fig. 2F). On the contrary, the overexpression of FTO-IT1 substantially enhanced the glycolytic capacity, which was further suppressed by treating with 2DG, an inhibitor of glycolysis (Supplementary Fig. S2A and B). Coincidently, the increased colony formation and proliferation ability of Huh7 cells induced by FTO-IT1 ectopic expression could be reversed by 2DG (Supplementary Fig. S2C-E). Together, these results suggested that FTO-IT1 promoted proliferation of HCC cells by enhancing glycolysis.

### FTO was a critical target for FTO-IT1 regulating glycolysis

**Fig. 3:**
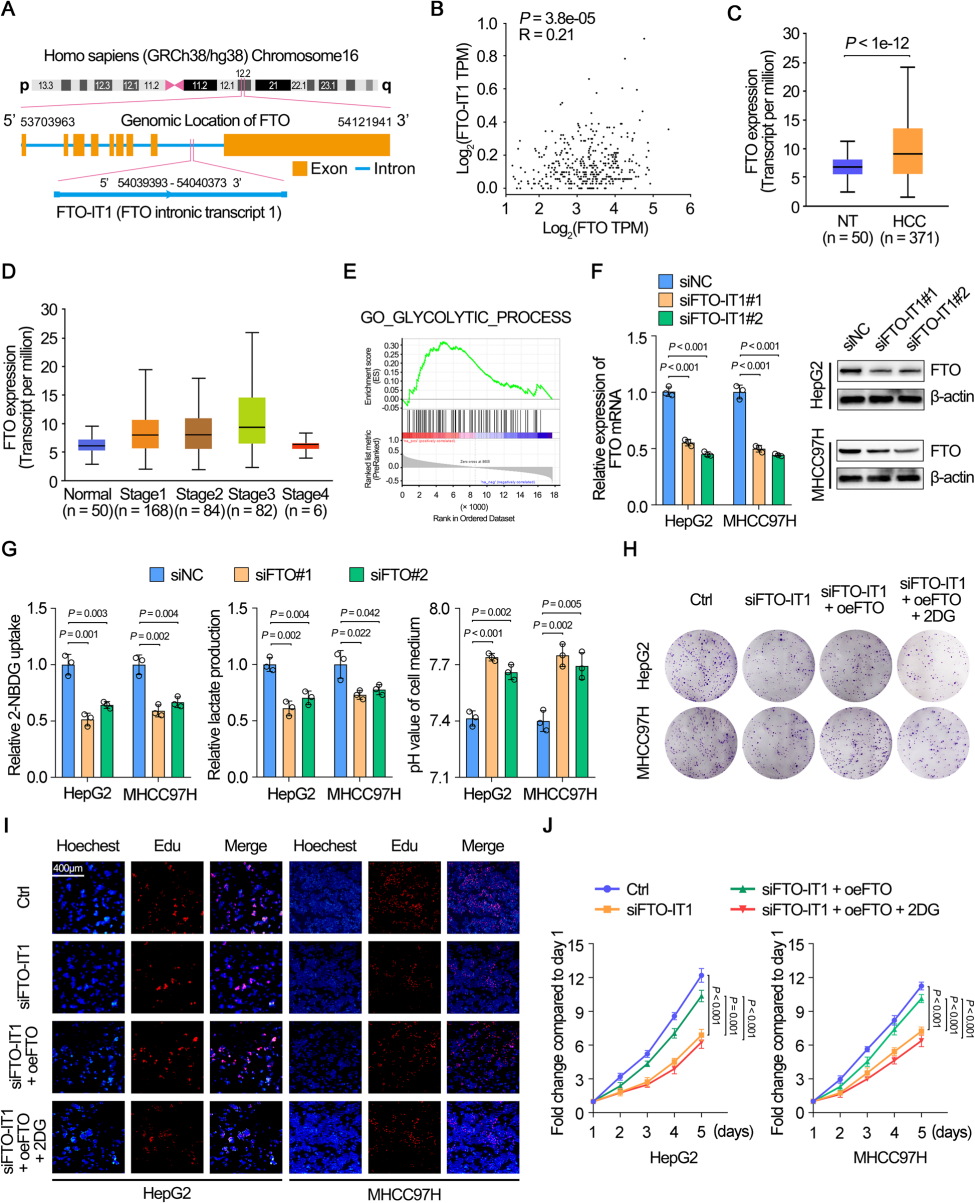
FTO was a critical target for FTO-IT1 regulating glycolysis. **A** Schematic illustration showing the genomic location of FTO-IT1 and FTO. **B** The expression correlation between FTO-IT1 and FTO in HCC tissues derived from the TCGA database. **C** The expression of FTO in HCC tissues and normal liver tissues by using the online database UALCAN (http://ualcan.path.uab.edu/analysis.html). NT: non-tumor. **D** The expression of FTO in different stages of HCC by using the online database UALCAN. **E** Gene set enrichment analysis of FTO-correlated genes in TCGA database. NES, normalized enrichment score. FDR, false discovery rate. **F** The mRNA (left) and protein (right) level of FTO after knocking down FTO-IT1. **G** The glucose uptake (left), lactate production (middle) and pH value (right) of HCC cells transfected with siNC or siFTO. **H**-**J** The proliferation of HepG2/MHCC97H cells transfected with siNC, siFTO-IT1 and empty vector or overexpression FTO plasmid and cultured with or without 2DG (5 mmol/L) was evaluated by colony formation assays (**H**), EdU assays (**I**) and MTT assays (**J**)

Since FTO-IT1 was an intronic lncRNA of FTO gene (Fig. 3A), we speculated whether FTO-IT1 exerted function in HCC by targeting FTO. Analysis from dataset of TCGA showed that FTO was positively correlated with FTO-IT1 (Fig. 3B). Meanwhile, FTO was remarkably overexpressed in HCC and correlated with advanced tumor stage (Fig. 3C and D). The expression of FTO was also much higher in MHCC97H and HepG2 cell lines which was highly similar to FTO-IT1 (Supplementary Fig. S3A). In addition, GSEA analysis displayed that FTO was associated with glycolysis process, glycolysis gluconeogenesis, glucose import, regulation of glucose metabolic process, glucose metabolic process (Fig. 3E; Supplementary Fig. S3B). Moreover, knockdown of FTO-IT1 resulted in dramatically decreased FTO in HCC cells at both the mRNA and protein levels (Fig. 3F), while the overexpression of FTO-IT1 showed the opposite effect (Supplementary Fig. S3C). We next investigated the effect of FTO on glycolysis in HCC cells. Results showed that knockdown of FTO significantly decreased, while overexpression of FTO obviously increased glucose uptake, lactate production, and the acidification of HepG2 and MHCC97H cells (Fig. 3G; Supplementary Fig. S3D and E). Coincidently, overexpression of FTO increased the colony formation and proliferation ability of Huh7 cells, which could be reversed by 2DG (Supplementary Fig. S3F-H). In addition, we have evaluated the regulation of migration, invasion and apoptosis of HCC cells by FTO. We found that FTO knockdown inhibited, while FTO overexpression promoted the migration and invasion metastasis of HCC cells. Moreover, knockdown of FTO induced apoptosis of HCC cells, but overexpression of FTO had no effect on apoptosis of HCC cells (Supplementary Fig. S4A-F). Meanwhile, the colony formation and proliferation of HepG2 and MHCC97H cells were remarkably inhibited by knockdown of FTO-IT1, but further rescued by overexpression of FTO (Fig. 3H-J). Furthermore, the overexpression of FTO-IT1 enhanced colony formation and proliferation of Huh7 cells were reversed by knockdown of FTO (Supplementary Fig. S3I-K). Thereby, our findings suggested that FTO was a critical target for FTO-IT1 to regulate glycolysis and proliferation of HCC cells.

### FTO-IT1 enhanced the interaction between ILF2 and ILF3 protein

**Fig. 4:**
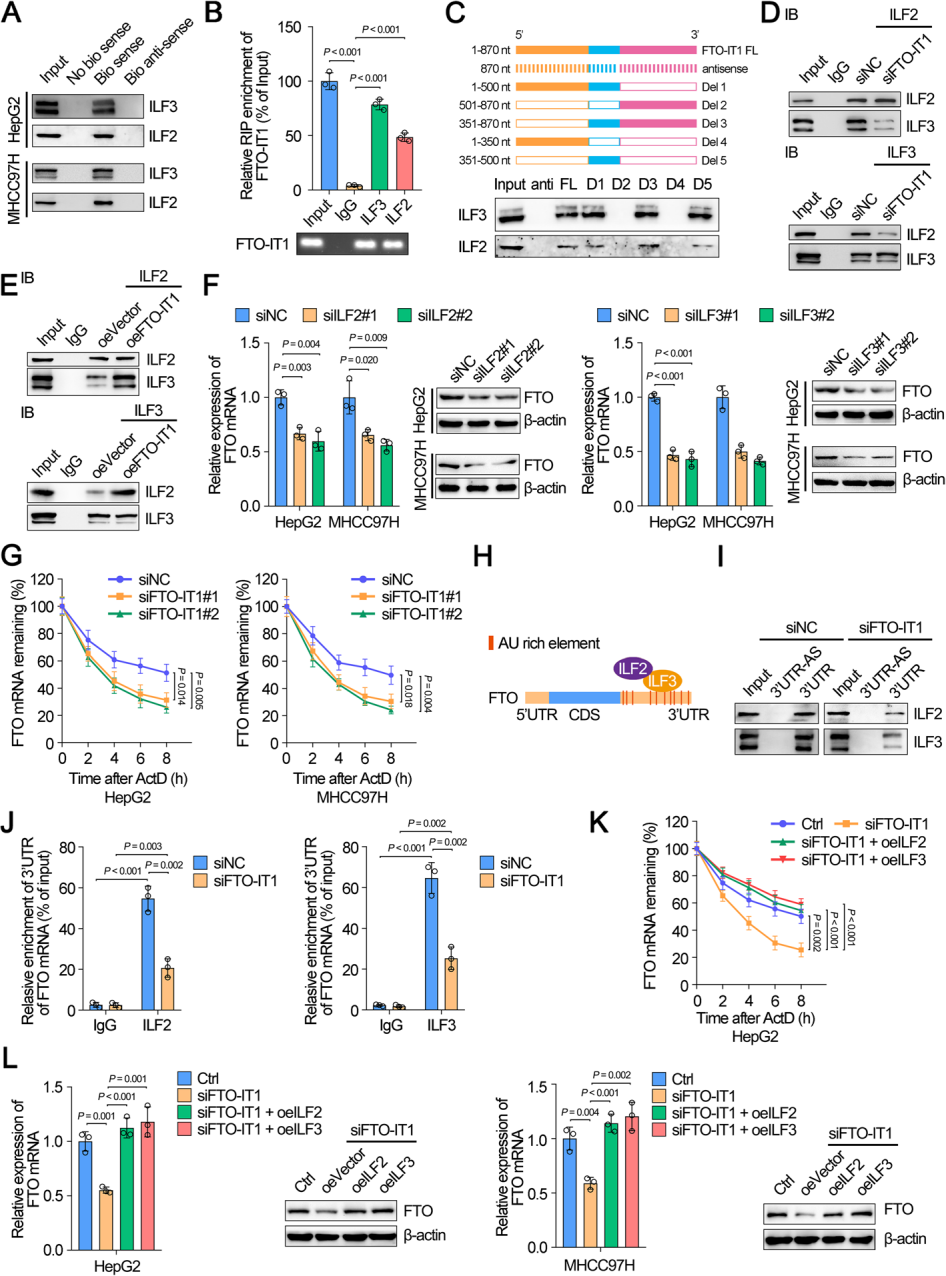
FTO-IT1 enhanced the interaction between ILF2 and ILF3 protein and stabilized FTO mRNA by strengthening the binding between ILF2/ILF3 complex and FTO mRNA. **A** Biotin-labeled RNA pull-down followed by Western blot showed the interaction between FTO-IT1 and ILF2/ILF3 protein in HepG2/MHCC97H cells. **B** RIP assay was applied using the anti-ILF2/ILF3 antibody and IgG antibody. The detection of FTO-IT1 (upper) was performed using specific primers in HepG2 cells. Then the PCR products were run on a 2% agarose gel (lower). **C** Western blot of ILF2/ILF3 pulled down by full-length (FL: 1–870 nt) or a series of FTO-IT1 truncates (Del 1–5) or antisense in HepG2 cells. **D**, **E** Co-IP of endogenous ILF2 and ILF3 with transfected siNC, siFTO-IT1 (**D**), empty vector or overexpression FTO-IT1 plasmid (**E**) in HepG2 cells. **F** The mRNA (left) and protein (right) levels of FTO in ILF2 knockdown or ILF3 knockdown HepG2/MHCC97H cells compared with control. **G** HepG2/MHCC97H cells transfected with siNC, siFTO-IT1, and then treated with ActD (5 μg/mL) for 0, 2, 4, 6 and 8 h, respectively, followed by qRT-PCR assays for FTO mRNA. **H** Schematic illustration showed the binding between ILF2/ILF3 and AREs in 3’UTR of FTO mRNA using online tool AREsite2 (http://rna.tbi.univie.ac.at/AREsite2/welcome). **I** Western blot of ILF2/ILF3 pulled down by 3’UTR of FTO or antisense (3’UTR-AS) in HepG2 cells transfected with siNC or siFTO-IT1. **J** RIP assays were applied using the anti-ILF2/ILF3 antibody and IgG antibody in HepG2 cells transfected with siNC or siFTO-IT1. qRT-PCR was used to detect the corresponding enrichment of 3’UTR of FTO mRNA. **K** HepG2 cells transfected with siNC, siFTO-IT1, or co-transfected with overexpression ILF2 or overexpression ILF3 plasmid and treated with ActD for corresponding hours followed by qRT-PCR assays for FTO mRNA. **L** The mRNA (left) and protein (right) levels of FTO in HepG2/MHCC97H cells transfected with siNC, siFTO-IT1 or co-transfected with overexpression ILF2 or overexpression ILF3 plasmid

To explore the underlying regulatory mechanism of FTO by FTO-IT1, we performed RNA pull-down assay followed by mass spectrometry using biotin-labeled FTO-IT1 as bait to identify FTO-IT1-interacting proteins in HepG2 cells. The results revealed that some proteins were pulled down by biotin-labeled FTO-IT1 but not FTO-IT1-antisense (Supplementary Table S4). Among those proteins, ILF2 and ILF3 had been reported to form protein complex and which could bind with AU-rich elements (AREs) in 3’UTR of target mRNA to enhance the post-transcriptional mRNA stabilization [10, 11]. Therefore, we selected ILF2 and ILF3 as targets for further exploration of the function and relative mechanism of FTO-IT1 in HCC. ILF2 and ILF3 were pulled down by biotin-labeled FTO-IT1 but not FTO-IT1-antisense, which were further identified by Western blot (Fig. 4A; Supplementary Fig. S5A). Moreover, the online tool *cat*RAPID indicated an intensive interaction between ILF2/ILF3 and FTO-IT1 (Supplementary Fig. S5B). Furthermore, RIP assay showed a significant accumulation of FTO-IT1 by anti-ILF2 or anti-ILF3 antibody but not IgG (Fig. 4B). Then, deletion-mapping analysis demonstrated that the 351-500nt region was indispensable for the binding of FTO-IT1 to ILF2 and ILF3 (Fig. 4C). Since ILF2 and ILF3 could interact with each other in a complex and function as regulator in various tumors [12–14], it was presumed that FTO-IT1 might strengthen the interaction between ILF2 and ILF3. Co-IP assays of ILF2 and ILF3 in the presence or absence of FTO-IT1 were performed to assess this hypothesis. Interestingly, the interaction between ILF2 and ILF3 was promoted by overexpression of FTO-IT1, while inhibited by knockdown of FTO-IT1 in HCC cells (Fig. 4D and E). We further explored whether FTO-IT1 could regulate the expression of ILF2 and ILF3. Indeed, knockdown or overexpression of FTO-IT1 had no effect on the mRNA or protein expression of ILF2 and ILF3 (Supplementary Fig. S5C-E). Meanwhile, knockdown or overexpression of ILF2 or ILF3 did not affect the expression of FTO-IT1 as well (Supplementary Fig. S5F-J). Altogether, these findings demonstrated a direct interaction between FTO-IT1 and ILF2/ILF3 protein complex.

### FTO-IT1 stabilized FTO mRNA by strengthening the binding between ILF2/ILF3 complex and FTO mRNA

Studies have shown that the function of ILF2/ILF3 complex to increase the stability of messenger RNA and, in some cases, as transcription factors [15, 16]. Thus, we speculated that the ILF2/ILF3 complex might play a role in regulating FTO mRNA. Coincidently, knockdown of ILF2 or ILF3 significantly decreased, while overexpression of ILF2 or ILF3 distinctively increased the mRNA and protein level of FTO (Fig. 4F; Supplementary Fig. S6A). Meanwhile, both knockdown or overexpression of FTO-IT1 did not affect transcription activity of FTO (Supplementary Fig. S6B and C). After treated with transcription inhibitor, actinomycin D (ActD), the mRNA stability of FTO was significantly impaired by knockdown of FTO-IT1, but enhanced by overexpression of FTO-IT1 (Fig. 4G; Supplementary Fig. S6D). These results implied that FTO-IT1 modulated the expression of FTO at post-transcriptional rather than transcription level. Coincidently, the analysis of AREsite2, a database for AU-rich elements, demonstrated 10 potential AREs in the 3’UTR of FTO mRNA (Fig. 4H). To confirm the combination between ILF2/ILF3 complex and FTO 3’UTR, RNA pull-down assays were performed using in vitro transcribed plasmids containing FTO 3’UTR or its antisense strand. Results revealed an obvious binding in 3’UTR of FTO mRNA which was weakened by knockdown of FTO-IT1, but strengthened by overexpression of FTO-IT1 (Fig. 4I; Supplementary Fig. S6E). In addition, RIP assays suggested significant enrichment of 3’UTR of FTO mRNA (Supplementary Fig. S6F), which was inhibited or enhanced by knockdown of FTO-IT1 or overexpression of FTO-IT1 (Fig. 4J; Supplementary Fig. S6G).

Subsequently, we investigated whether FTO-IT1 affected the interaction between ILF2/3 complex and FTO mRNA. Knockdown of FTO-IT1 obviously impaired mRNA stability of FTO, which could be rescued by overexpression of ILF2 or ILF3 (Fig. 4K). Conversely, overexpression of FTO-IT1 improved mRNA stability of FTO, which was repressed by knockdown of ILF2 or ILF3 (Supplementary Fig. S6H). Coincidently, the decreased expression of FTO in HepG2 and MHCC97H cells by treatment with knockdown of FTO-IT1 was visibly rescued by overexpression of ILF2 or ILF3, while the increased expression of FTO in Huh7 cells by overexpression of FTO-IT1 was reversed by knockdown of ILF2 or ILF3 (Fig. 4L; Supplementary Fig. S6I). Together, these results clearly indicated that FTO-IT1 stabilized FTO mRNA and promoted FTO expression in post-transcriptional level through enhancing the interaction between ILF2/ILF3 complex and FTO mRNA.

### FTO facilitated glycolysis in HCC cells by targeting glycolytic enzymes

**Fig. 5:**
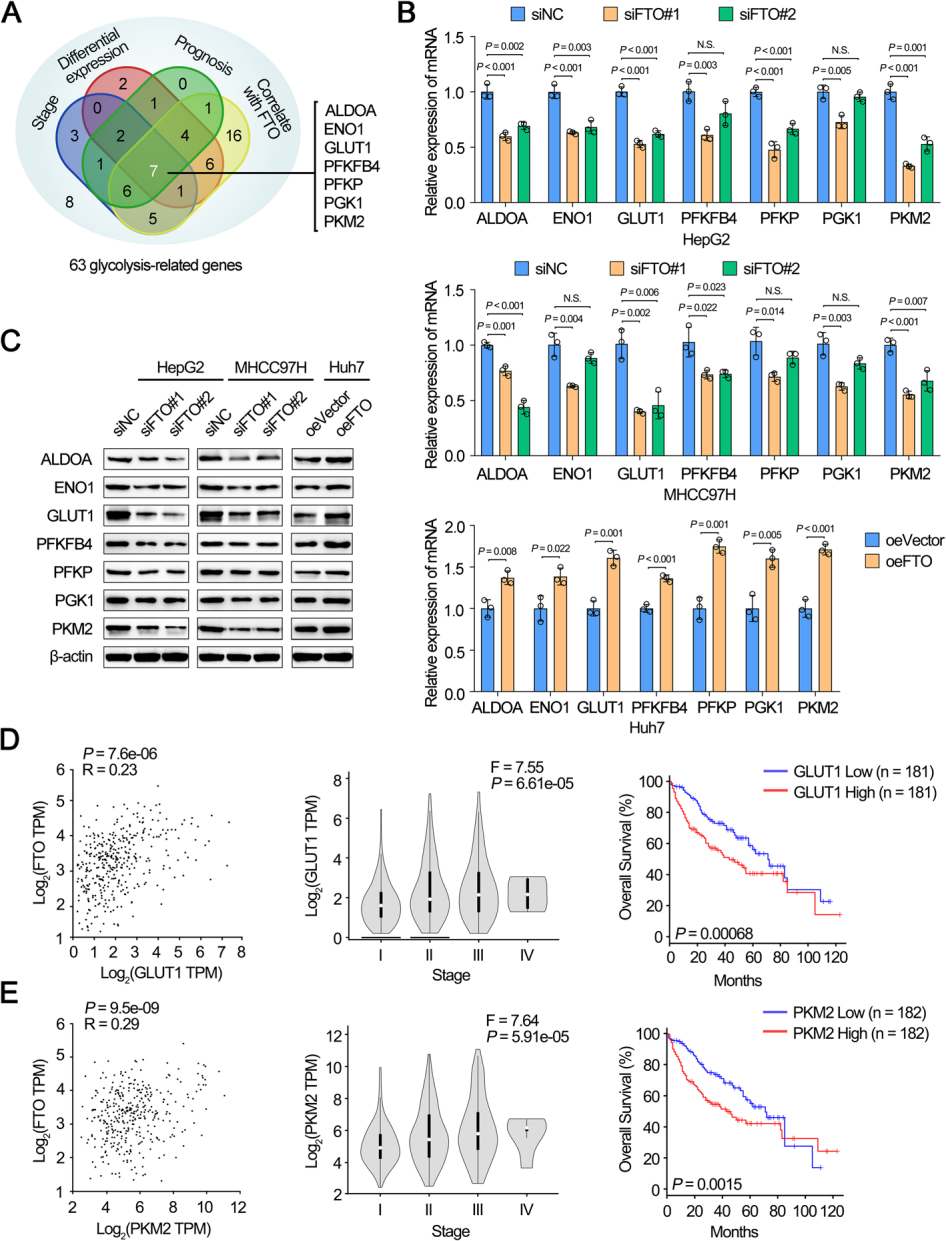
FTO facilitated glycolysis in HCC cells by targeting glycolytic enzymes. **A** Venn diagram indicating the identification of glycolysis-related genes were differentially expressed (fold change > 2.0, *P* < 0.05) in TCGA LIHC datasets with clinical stages, prognosis of survival and correlation with FTO (*R* > 0, *P* < 0.05). **B**, **C** The mRNA (**B**) and protein (**C**) expression of glycolysis-related genes in HepG2/MHCC97H cells transfected with siNC, siFTO or in Huh7 cells transfected with empty vector, overexpression FTO plasmid. **D**, **E** The correlation of the expression between FTO and GLUT1 (**D**) or PKM2 (**E**) (left). The relative transcript levels of GLUT1 (**D**) or PKM2 (**E**) in HCC tissues with different pathological stages (middle). Kaplan–Meier analyses of OS in HCC patients with low and high levels of GLUT1 (**D**) or PKM2 (**E**) (right) from GEPIA online database

Subsequently, we investigated the molecular mechanism by which FTO regulated glycolysis. We first analyzed the expression correlation of FTO and 63 genes which were involved in glucose transport and glucose metabolism in HCC samples from TCGA dataset (Supplementary Table S5). Among those 63 genes, 7 glycolytic genes (ALDOA, ENO1, GLUT1, PEKFB4, PFKP, PGK1, PKM2; Fig. 5A) were overexpressed (fold change > 2.0, *P* < 0.05) in HCC and correlated with advanced tumor stages and poor prognosis, respectively. Meanwhile, qRT-PCR and Western blot revealed that GLUT1 and PKM2 showed the most obvious fold change at mRNA and protein level by both knockdown and overexpression of FTO (Fig. 5B and C). Meanwhile, analysis from online dataset GEPIA demonstrated that the 7 glycolytic genes were positively related to FTO expression, as well as late tumor stage and poor OS (Fig. 5D and E; Supplementary Fig. S7A-E). The expression of GLUT1 or PKM2 were positively correlated to FTO in HCC and the stage of HCC. Besides, high GLUT1 or PKM2 levels were associated with poor OS of HCC patients. Thereby, these results suggested that FTO functioned as an activator in glycolysis by regulating glycolysis-related genes of HCC cells.

### FTO-IT1/FTO signaling increased mRNA stabilization of GLUT1 and PKM2 by decreasing m^6^A modification

**Fig. 6:**
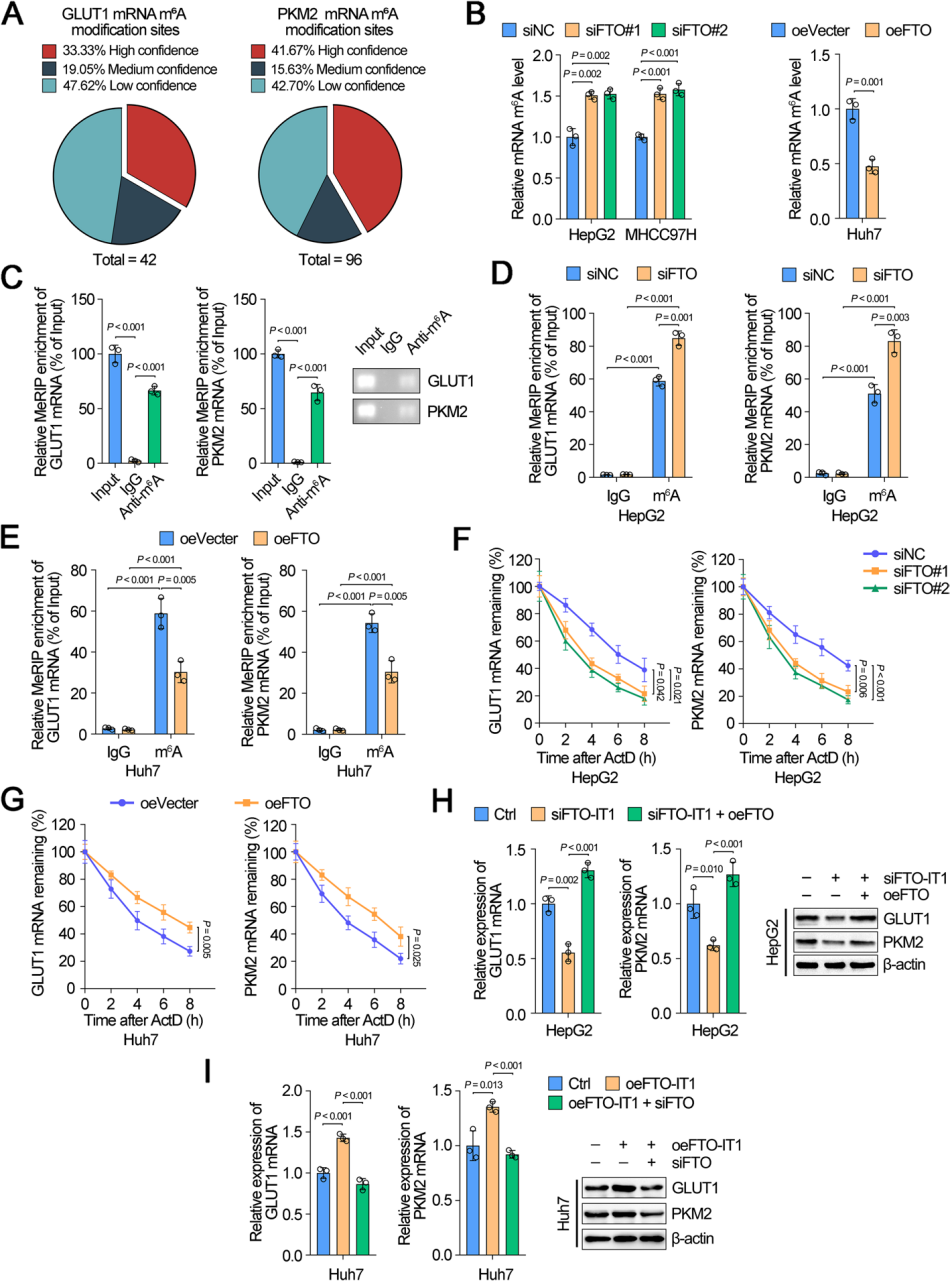
FTO-IT1/FTO signaling increased mRNA stabilization of GLUT1 and PKM2 by decreasing m^6^A modification. **A** Pie charts analysis showing the m^6^A modification sites of all sources of GLUT1 and PKM2 from the RMvar online database (https://rmvar.renlab.org/index.html). **B** Global m^6^A levels in mRNA were detected using colorimetric method in HepG2/MHCC97H cells transfected with siNC, siFTO (left) or in Huh7 cells transfected with empty vector, overexpression FTO plasmid (right). **C** MeRIP assays were applied using the anti-m^6^A antibody and IgG antibody in HepG2 cells. qRT-PCR was used to detect the corresponding enrichment of GLUT1 and PKM2. **D**, **E** MeRIP assays were applied using the anti-m^6^A antibody and IgG antibody in HepG2 cells transfected with siNC, siFTO (**D**) or in Huh7 cells transfected with empty vector, overexpression FTO plasmid (**E**) to detect the corresponding enrichment of GLUT1 and PKM2. **F**, **G** Cells transfected with siNC, siFTO-IT1 (**F**), or empty vector, overexpression FTO plasmid (**G**), and treated with ActD for corresponding hours followed by qRT-PCR assays for GLUT1 and PKM2 mRNA. **H** The mRNA (left) and protein (right) levels of GLUT1 and PKM2 in HepG2 cells transfected with siFTO-IT1 and/or overexpression FTO plasmid. **I** The mRNA (left) and protein (right) levels of GLUT1 and PKM2 in Huh7 cells transfected with overexpression FTO-IT1 plasmid and/or siFTO

Since GLUT1 and PKM2 were the two most significantly regulated genes by FTO, we further explored the regulatory mechanism of FTO on GLUT1 and PKM2. FTO is a demethylase function as erasing role in the reversible methylation and has been reported to play an oncogenic role in various cancers [17, 18]. Considering the essential role of FTO in m^6^A regulation, we wonder whether FTO regulated expression of GLUT1 and PKM2 by m^6^A modification. Coincidently, the online dataset for RNA modification displayed that more than one third high-confidence m^6^A modification sites on GLUT1 and PKM2 mRNAs (Fig. 6A). Meanwhile, knockdown of FTO obviously increased m^6^A level of total mRNAs in HepG2 and MHCC97H cells, while overexpression of FTO significantly impaired m^6^A level of total mRNAs in Huh7 cells (Fig. 6B). Subsequently, we assessed the existence of m^6^A modification on mRNA of GLUT1 and PKM2 using MeRIP. As expected, the enrichment of GLUT1 or PKM2 mRNA fragments was immunoprecipitated with m^6^A antibodies but not IgG (Fig. 6C). Moreover, knockdown of FTO increased, while overexpression of FTO decreased the enrichment of GLUT1 or PKM2 RNA fragments by m^6^A antibodies (Fig. 6D and E). Specifically, knockdown of FTO crucially reduced the mRNA stability of GLUT1 and PKM2 (Fig. 6F), whereas overexpression of FTO induced their stability (Fig. 6G). In addition, knockdown of FTO-IT1 invisibly inhibited expression of GLTU1 and PKM2 in HepG2 cells, which could be rescued by overexpression of FTO (Fig. 6H). Contrarily, overexpression of FTO-IT1 distinctively induced expression of GLUT1 and PKM2 in Huh7 cells, which was reversed by knockdown of FTO (Fig. 6I). These data demonstrated that FTO-IT1/FTO signaling stabilized mRNA of GLUT1 and PKM2 by m^6^A demethylation in HCC cells.

### FTO-mediated demethylation inhibited YTHDF2-mediated mRNA degradation of GLUT1 and PKM2

The function of m^6^A in regulating gene expression is executed mostly through the readers, including YTH domain-containing family proteins in mammalian cells [19]. A set of m^6^A-modified transcripts could be recognized by the YTHDF2 reader, which exhibits a shorter half-life than non-methylated ones [20]. RIP assays were conducted to investigated whether YTHDF2 could bind to GLUT1 and PKM2 mRNA. The results showed that mRNA of GLUT1 and PKM2 were critically enriched on anti-YTHDF2 antibody compared with IgG (Supplementary Fig. S8A). Moreover, knockdown of FTO increased, while overexpression of FTO decreased the enrichment of GLUT1 and PKM2 mRNA on anti-YTHDF2 antibody (Supplementary Fig. S8B and C). As expected, knockdown of YTHDF2 noticeably rescued the stability and expression of GLUT1 and PKM2 in FTO-knockdown HCC cells (Supplementary Fig. S8D-F). On the contrary, overexpression of FTO increased stability and expression of GLUT1 and PKM2 in Huh7 cell, which was considerably inhibited by overexpression of YTHDF2 (Supplementary Fig. S8G and H). Together, these findings indicated that the m^6^A demethylation caused by FTO regulated the expression of GLUT1 and PKM2 by influencing the stability of GLUT1 and PKM2 mRNA transcripts by the way of YTHDF2-dependent mechanism.

### FTO-IT1 was transcriptionally regulated by c-Myc under hypo-glucose condition

**Fig. 7:**
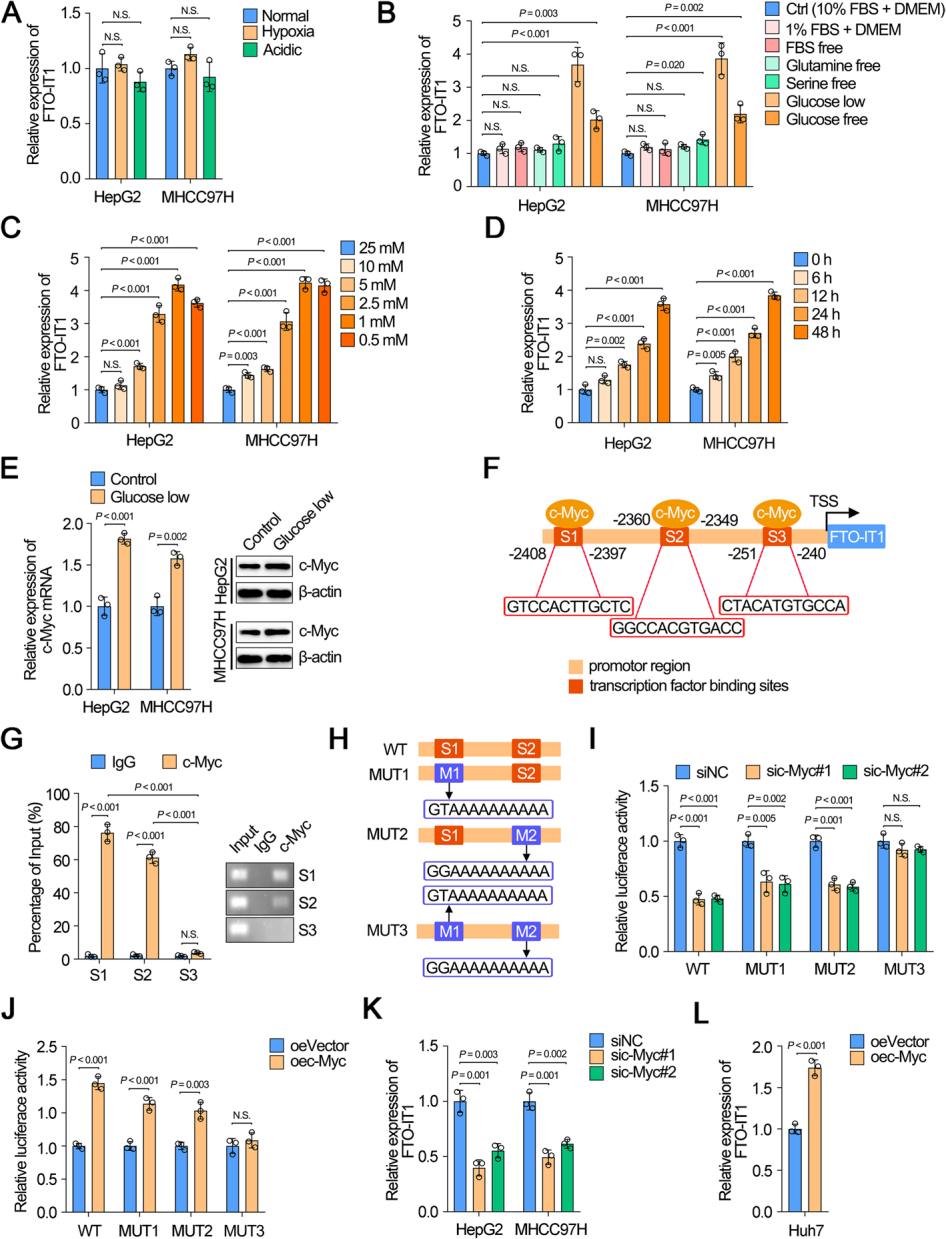
FTO-IT1 was transcriptionally regulated by c-Myc under hypo-glucose condition. **A** Expressions of FTO-IT1 in HepG2/MHCC97H cells were detected under hypoxia and acidic conditions compared with normal condition. **B** Expressions of FTO-IT1 in HepG2/MHCC97H cells were detected under 1% FBS, FBS free, glutamine-free, serine-free, glucose low (1 mmol/L) and glucose-free conditions compared with normal condition. **C** Cells were cultured with sequentially decreased concentration of glucose conditions (25, 10, 5, 2.5, 1, 0.5 mmol/L), then the FTO-IT1 level was evaluated. **D** Cells were exposed to low glucose condition (1 mmol/L) for 0, 6, 12, 24 and 48 h, then the FTO-IT1 level was evaluated. **E** Cells were cultured with low glucose conditions (1 mmol/L), then the mRNA and protein level of c-Myc were evaluated. **F** Schematic illustration of the proximal region of the FTO-IT1 promoter and three putative c-Myc binding sites using JASPAR database (https://jaspar.genereg.net). TSS, Transcriptional start site. **G** ChIP assays were performed to verify the binding capacity between c-Myc and the three binding sites of FTO-IT1 promoter in HepG2 cells (left). Then the qRT-PCR products were separated by 2% agarose gel electrophoresis (right). **H** The potential binding site was mutated as indicated. WT, wild-type; MUT, mutation. **I** WT or MUT FTO-IT1 promoter sequence luciferase reporter activity in HepG2 cells transfected with or without sic-Myc were measured by luciferase reporter assay. Renilla luciferase served as a control. **J** WT or MUT FTO-IT1 promoter sequence luciferase reporter activity in Huh7 cells transfected with or without overexpression c-Myc plasmid were measured by luciferase reporter assay. **K**, **L** The level of FTO-IT1 after knocking down (**K**) or overexpressing (**L**) c-Myc

The energy balance is the key to adapt cells to the tumor microenvironment in cancer cells, which tends to show low nutritional. We first examined the expression of FTO-IT1 under hypoxic and acidic conditions, as these two conditions were the most common in microenvironments. However, there were no change in FTO-IT1 expression (Fig. 7A). Thus, we further explored the potential mechanism of upregulation of FTO-IT1 in energy stress. Compared with normal condition, FTO-IT1 was markedly increased under hypo-glucose condition, while FTO-IT1 showed no significant change in other environments such as 1% FBS, FBS free, glutamine-free, and serine-free, and FTO-IT1 was slightly increased under glucose-free condition (Fig. 7B). Moreover, the increased expression of FTO-IT1 was concentration dependent as well as time dependent under hypo-glucose condition. (Fig. 7C and D). As expected, the mRNA and protein levels of FTO also indirectly increased with decreasing glucose concentration (Supplementary Fig. S9A).

c-Myc is a multifunctional transcription factor that is deregulated in many human cancers and a key regulator for cancer cell metabolism [21]. Research revealed that c-Myc protein levels was increased in specific cells during glucose deprivation [22]. Therefore, we assumed whether glucose deprivation-induced FTO-IT1 was regulated by c-Myc. Coincidently, c-Myc was increased in both HepG2 and MHCC97H cells during glucose deprivation condition (Fig. 7E). Meanwhile, sequence analysis by JASPAR demonstrated three putative binding sites (S1: -2408 to -2397, S2: -2360 to -2349, S3: -251 to -240; Supplementary Table S6) in the FTO-IT1 promoter region for the transcription factor c-Myc (Fig. 7F). Subsequently, ChIP assay suggested obvious enrichment of S1 and S2 by anti-c-Myc antibody but not S3 (Fig. 7G). We further constructed dual-luciferase reporter plasmids containing wild type (WT) or mutant sequence (MUT1-3) of promoter and then transfected into HCC cells (Fig. 7H). Results showed the luciferase activities of WT, MUT1, MUT2 groups but not MUT3 group were obviously attenuated by knockdown of c-Myc, while enhanced by overexpression of c-Myc, which indicated both S1 and S2 were required for the binding between c-Myc and FTO-IT1 promoter (Fig. 7I and J). Furthermore, FTO-IT1 was significantly inhibited by knockdown of c-Myc, while overexpression of c-Myc could promoted the expression of FTO-IT1 in HCC cells (Fig. 7K and L; Supplementary Fig. S9B).

### c-Myc was reciprocally regulated by FTO-IT1 via FTO-mediated m^6^A demethylation

Research reported that FTO decreased m^6^A modification and stabilized mRNA of c-Myc in leukemia cells [23], thereafter, we hypothesized that FTO-IT1 might regulated c-Myc expression to form a reciprocal feedback. Coincidently, overexpression of FTO-IT1 increased, while knockdown of FTO-IT1 inhibited c-Myc expression both at mRNA and protein level (Supplementary Fig. S9C and D). Meanwhile, analysis of RMvar database showed that there were a large number of highly credible m^6^A modification sites in mRNA of c-Myc (Supplementary Fig. S9E). Furthermore, MeRIP results showed that c-Myc mRNA was greatly enriched by m^6^A antibody (Supplementary Fig. S9F). Moreover, FTO-IT1 knockdown or overexpression obviously increased or decreased enrichment of c-Myc on m^6^A antibody, which was successfully reversed or rescued by FTO overexpression or knockdown (Supplementary Fig. S9G and H). Similarly, the weakened or strengthened stability of c-Myc mRNA induced by FTO-IT1 knockdown or overexpression could be noticeably rescued or reversed by FTO overexpression or knockdown (Supplementary Fig. S9I and J).

### FTO-IT1/FTO signaling promoted proliferation of HCC cells in vivo

**Fig. 8:**
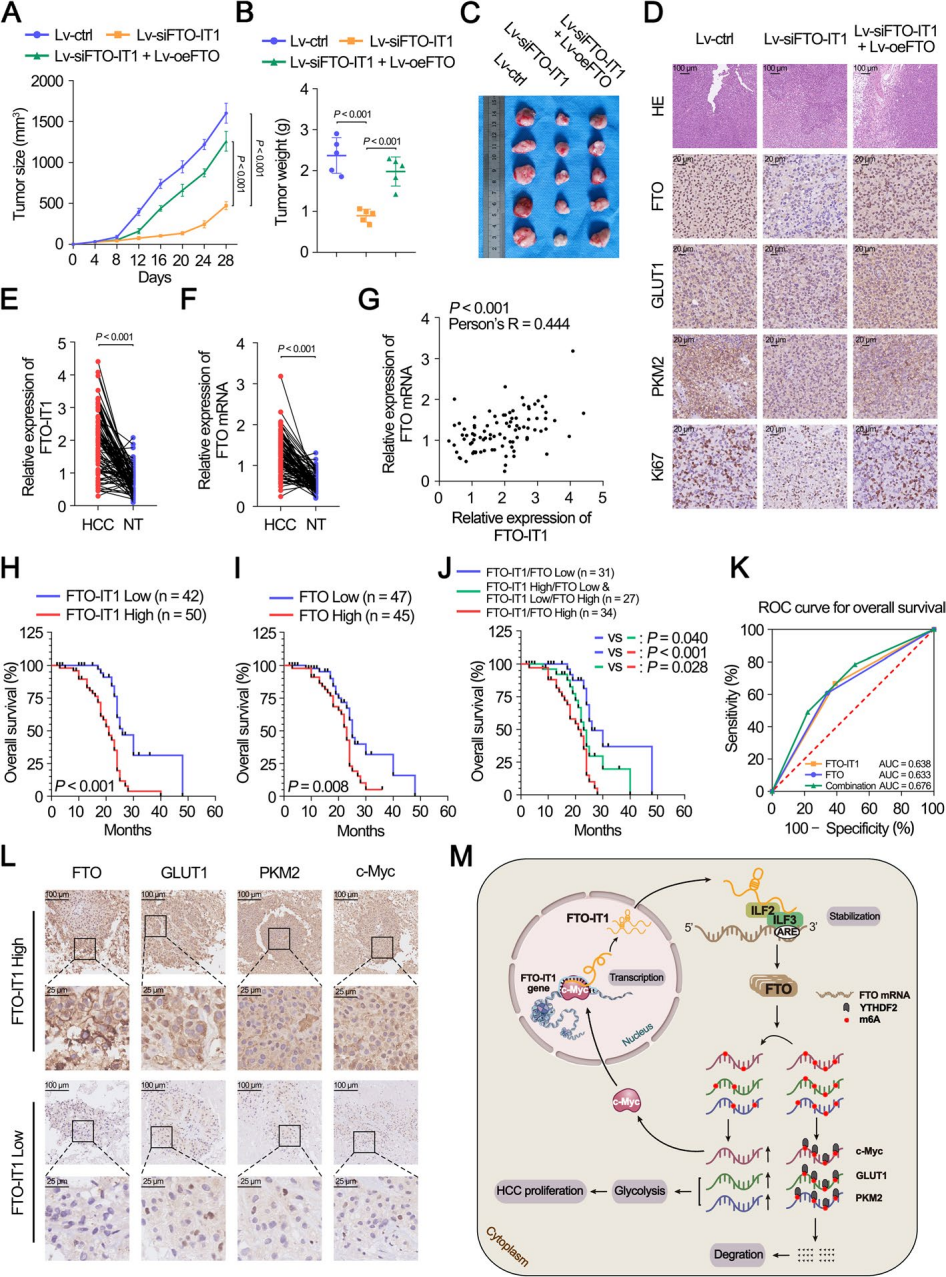
FTO-IT1/FTO signaling promoted proliferation of HCC cells in vivo and correlated with poor clinical outcomes. **A**-**C** Subcutaneous implantation mouse models were established by using MHCC97H cells that were transfected with lentivirus containing siFTO-IT1 plasmid and/or overexpression FTO plasmid. Growth curve (**A**), tumor weight (**B**) and representative images (**C**) of xenografts (*n* = 5) in the three treatment groups for 28 days were shown. **D** Representative HE and IHC staining for FTO, GLUT1, PKM2 and Ki67 expression in the subcutaneous xenografts. The xenografts were collected 4 weeks after tumor implantation. **E**, **F** The relative expression of FTO-IT1 (**E**) and FTO mRNA (**F**) was detected in 92 pairs HCC clinical specimens and matched paracancerous tissues by qRT-PCR. NT: non-tumor. **G** The expression correlation between FTO-IT1 and FTO in 92 pairs HCC clinical specimens and matched paracancerous tissues. NT: non-tumor. **H** Kaplan–Meier curves showed OS of 92 HCC patients with high or low FTO-IT1 expression. **I** Kaplan–Meier curves showed OS of 92 HCC patients with high or low FTO expression. **J** 92 HCC patients were divided into three groups based on the expression level of FTO-IT1 and FTO as indicated. OS of those patients was evaluated via Kaplan–Meier analysis. **K** ROC curve analysis for OS for FTO-IT1 (*P* = 0.023), FTO (*P* = 0.029) as individual biomarkers or for the combined panel (*P* = 0.004). Setting parameters were as follows: death as positive event; score of high FTO-IT1 or FTO samples was 1, while low FTO-IT1 or FTO samples was 0. Score of the combined panel was the sum of the scores of each sample. Area under the curve (AUC) was used to evaluate predictive capability. **L** The representative IHC images of FTO, GLUT1, PKM2 and c-Myc in 92 HCC tissues with high or low levels of FTO-IT1. Scale bar, 100/25 μm. **M** Schematic illustration of FTO-IT1/FTO/c-Myc axis maintaining the malignancy of HCC

To further evaluate the biological function of FTO-IT1 in vivo, HCC cells stably transfected with lentivirus containing plasmids were transplanted subcutaneously or orthotopically into the nude mice respectively. A significant decrease in both tumor growth and weight was observed in Lv-siFTO-IT1 group compared to Lv-ctrl group. Nevertheless, co-transfection with Lv-oeFTO restored the tumor growth and weight alleviated by knockdown of FTO-IT1 (Fig. 8A-C). Moreover, the IHC staining revealed that FTO, GLUT1 and PKM2 were reduced in Lv-siFTO-IT1 group (Fig. 8D). Ki-67 staining was also decreased in tumors derived from implantation of Lv-siFTO-IT1 group compared with control (Fig. 8D), indicating impaired proliferation of tumor cells. Consistently, tumor size and weight, and expression of FTO, GLUT1, PKM2 and Ki67 increased in Lv-oeFTO-IT1 group compared to Lv-ctrl group, while co-transfection with Lv-siFTO reversed the tumor size and weight, and expression of FTO, GLUT1, PKM2 and Ki67 facilitated by overexpression of FTO-IT1 (Supplementary Fig. S10A-D). The orthotopic transplants displayed a similar trend in the tumor size and weight, and expression of above proteins (Supplementary Fig. S10E-G).

### FTO-IT1/FTO signaling was upregulated in HCC patients and correlated with poor clinical outcomes

We then examined the expression of FTO-IT1 and FTO in 92 paired HCC and paracancerous tissues (Supplementary Tables S7 and S8). The results showed that FTO-IT1 and FTO were highly expressed (Fig. 8E and F; Supplementary Fig. S10H) and positively correlated in HCC (Fig. 8G). Moreover, HCC patients with higher FTO-IT1 or FTO expression displayed a poorer OS (Fig. 8H and I). Further survival analysis showed that low level of both FTO-IT1 and FTO was much more beneficial to OS of HCC patients, which followed by low level of either FTO-IT1 or FTO, while high level of both FTO-IT1 and FTO was unfavorable for prognosis (Fig. 8J). Moreover, the combination FTO-IT1 and FTO showed a better predictive value for OS compared with any individual target in the ROC curve analysis (Fig. 8K). The IHC and *chi*-square test showed that FTO, GLUT1, PKM2 and c-Myc all increased in HCC patients with high FTO-IT1 expression (Fig. 8L; Supplementary Fig. S10I). In addition, the expression of FTO was also positively correlation with expressions of GLUT1, PKM2 and c-Myc (Supplementary Fig. S10J). Taken together, these results suggested an important role of the FTO-IT1/FTO/c-Myc axis in maintaining the malignancy of HCC (Fig. 8M).

## Discussion

The role of energy metabolism on the progression of cancers has gradually attracted attention. Metabolic change mechanistically linked to self-sufficiency in growth signals, evading apoptosis, limitless replicative potential, sustained angiogenesis, tissue invasion and metastasis and avoidance of immunosurveillance of cancers [24]. Aerobic glycolysis is one of the important metabolic modes of cancers. Mounting evidences have suggested that lncRNAs play crucial regulatory roles in glycolysis of cancers through diverse mechanisms. For example, lncRNA GLCC1 stabilized c-Myc from ubiquitination and further specifies the transcriptional modification pattern on c-Myc target genes, such as LDHA, consequently reprogram glycolytic metabolism for CRC proliferation [25]. Our previous research revealed that lncRNA DICER1‑AS1 promoted proliferation and metastasis of pancreatic cancer by enhancing glycolysis through modulating the DICER1/miR‑5586‑5p axis [26]. However, the role of lncRNAs in glycolysis of HCC are still not well understood. Our present study demonstrated that FTO-IT1 was highly expressed in HCC samples, and associated with poor prognosis of HCC. Meanwhile, we found that FTO-IT1 promoted proliferation of HCC cells in glycolysis-dependent manner. Therefore, our study identified a crucial onco-lncRNA which constructed a bridge between glycolysis and progression of HCC.

The regulation of carcinogenesis by lncRNA often requires mediation of key proteins. FTO-IT1 is a lncRNA encoded by the intron of FTO gene. Coincidentally, FTO was highly expressed in HCC and positively correlated with FTO-IT1 in HCC tissue. More importantly, FTO was also found to be positively correlated with cancer glycolysis through bioinformatics analysis. Therefore, we speculated that FTO was downstream target of FTO-IT1. Knockdown and overexpression experiments confirmed that FTO-IT1 could regulate the expression of FTO both at mRNA and protein levels, and alter the biological effects of HCC through FTO. Notably, antisense lncRNA was revealed to regulate the expression of the sense gene via various mechanisms by quite a few reports [27, 28], but intronic lncRNA has received less attention in the past researches, let alone the relationship between the intronic lncRNA and the transcript encoded by same gene. Current study identified that overexpression of intronic lncRNA RUNX1-IT1 impaired the growth, metastasis and stem-like features of HCC cells in vivo by modulating GSK-3β/WNT/β-catenin pathway [29]. LncRNA CPS1-IT1 suppressed the metastasis of HCC by regulating HIF-1α activity and inhibiting epithelial-mesenchymal transition [30]. Therefore, our study provided novel evidence for intronic lncRNA to regulate the expression of transcript encoded by same gene.

Majority of previous studies demonstrated that lncRNAs regulated expression of targets by means of specific protein. Our results of RNA pull-down assay followed by mass spectrometry and RIP assays indicated that ILF2 and ILF3 were directed binding with FTO-IT1. ILF3, also known as NF90/NF110, encodes a double-stranded RNA binding protein that formed a heterodimeric complex with ILF2 protein, also known as NF45. This mode of heterodimeric complex increased the respective protein stability of ILF2 and ILF3, contributing to exert their coregulation function [31]. Co-IP assays confirmed that FTO-IT1 increased the binding of ILF2 and ILF3, which consequently enhanced the regulation of FTO by ILF2/ILF3 protein complex. This result suggested the key role of FTO-IT1 in the regulation of FTO. Meanwhile, it provided new evidence for the lncRNA regulating the binding compactness of protein complex.

LncRNAs have been illustrated to regulate target molecules in transcription or post-transcription manner. For example, lncRNA ZNFTR interacted with ATF3 and sequestered ATF3 away from the ZNF24 promoter, which consequently increased the expression of ZNF24 in transcriptional level [32]. HIF1A-AS1 post-transcriptionally augmented HIF-1α expression to promote gemcitabine resistance of pancreatic cancer by enhancing glycolysis [33]. Interestingly, function assays confirmed that ILF2/ILF3 protein complex promoted the expression of FTO by the means of maintaining mRNA stability instead of activating transcription. This finding improved our understanding of the post-transcription regulatory function of lncRNAs in cancers.

m^6^A exist internally in eukaryotic mRNA and can affect the metabolism and function of mRNA. Furthermore, FTO, the key “eraser” of m^6^A, has been confirmed to be an essential regulator in several cancers. For example, FTO was entrusted crucial role as an m^6^A demethylase in promoting melanoma tumorigenesis and anti-PD-1 resistance [34]. Another research found that the specific inhibitor of FTO dramatically suppressed glioblastoma stem cell self-renewal and tumorigenesis [35]. In acute myeloid leukemia, FTO was directly defined as an oncogene when it played a m^6^A RNA demethylase role [36]. Recent studies have gradually begun to focus on the relationship between m^6^A and cancer metabolism, especially aerobic glycolysis. It was found that FTO-dependent m^6^A regulates the progression of endometriosis via the ATG5/PKM2 Axis [37]. Another study suggested that FTO suppressed glycolysis and growth of papillary thyroid cancer via decreasing stability of APOE mRNA in an m^6^A dependent manner [38]. However, the status of FTO and the underlying regulatory mechanism in HCC, especially in the glycolytic metabolism remains little known. Glycolytic assays revealed that FTO facilitated the glycolysis level of HCC cells. Meanwhile, FTO promoted the expression of multiple key glycolytic proteins. These results further suggested that FTO had potentially important implications for cancer progression and glycolysis.

Through the screening of bioinformatics material including the clinical data, we selected GLUT1 and PKM2 as the potential glycolytic proteins of FTO regulating glycolysis in HCC cells. FTO altered the m^6^A levels of GLUT1 and PKM2 and consequentially regulated their expression. These results indicated that GLUT1 and PKM2 likely played a critical role as the key targets of FTO-IT1/FTO signal in the pathogenesis of HCC. Previous studies suggested that mRNA transcripts with m^6^A modifications tended to be regulated by YTHDFs or IGF2BPs as the direct m^6^A readers [39, 40]. YTHDF2 is a pivotal member of the m^6^A readers, which can specifically reduce the expression of genes by promoting their mRNA decay [20]. For instance, YTHDF2 facilitated m^6^A-dependent mRNA decay of LXRA and HIVEP2, which impacted the glioma patient survival [41]. Consistently, our results demonstrated that YTHDF2 inhibited the mRNA stability of GLUT1 and PKM2, a process that was simultaneously regulated by FTO. Thus, we revealed the concrete mechanism and targets of FTO-IT1/FTO signal regulating glycolytic metabolism of HCC cells in m^6^A-depentent manner. Meanwhile, our findings provided a novel and essential evidence that the crosstalk between lncRNA and m^6^A modifications especially caused by FTO regulated cancer progression and metabolism.

Glucose deprivation induced the expression differential of FTO-IT1 and c-Myc implied that c-Myc might involve the regulation of FTO-IT1. Function assays validated the transcription activation of c-Myc on FTO-IT1. c-Myc was found to function as a key oncogene by forming feedback loops with various molecules. We previously reported reciprocal feedback between lncRNA GLS-AS and c-Myc, was involved in the proliferation and invasion of pancreatic cancer during glutamine deprivation [42]. Meanwhile, c-Myc was regulated by FTO via m^6^A modification in HCC, which was similarity in leukemia, colorectal cancer, gastric cancer, glioma and lung adenocarcinoma [23, 43–46]. Therefore, this study provided an evidence that FTO-IT1 reciprocally regulated the expression of c-Myc via to form a reciprocal feedback in HCC.

## Conclusions

Collectively, the present study implicated a novel pathway of FTO-IT1/FTO/c-Myc which promoted progression of HCC in glycolysis-dependent manner. FTO-IT1 recruited the ILF2 and ILF3 proteins to facilitate the expression level of FTO. Then FTO upregulated the expression of GLUT1 and PKM2 with the mediation of YTHDF2 in m^6^A-dependent manner. FTO-IT1 and c-Myc form a positive feedback loop to promote this regulation. It was suggested that the FTO-IT1 was a potentially valuable therapeutic target to enhance the treatment response in HCC.

### Supplementary Information

**Additional file 1:** **Supplementary Figure S1.** FTO-IT1 was a lncRNA upregulated in HCC samples and associated with poor prognosis. A, B. GO (A) and KEGG (B) analyses of FTO-IT1-correlated genes in HCC tissues from TCGA database. C. The sequence of FTO-IT1 transcript from the Nucleotide database in NCBI. D. Predicted secondary structure of FTO-IT1 from the RNAfold web server (http://rna.tbi.univie.ac.at/cgi-bin/RNAWebSuite/RNAfold.cgi). E-F. The Coding potentials of FTO-IT1 were evaluated using CPC2 (E, http://cpc2.cbi.pku.edu.cn) and PyhloCSF (F). **Supplementary Figure S2. **FTO-IT1 promoted glycolysis and proliferation of HCC cells. A. Overexpression efficiency of pcDNA-FTO-IT1 plasmid was assessed via transfection assay followed by qRT-PCR. B. The glucose uptake (left), lactate production (middle) and pH value (right) of HCC cells transfected with empty vector or overexpression FTO-IT1 plasmid with or without the treatment of 2DG. C-E. Colony formation assays (C), EdU assays (D) and MTT assays (E) depicting the change in viability of Huh7 cells transfected with overexpression FTO-IT1 plasmid with or without the treatment of 2DG. **Supplementary Figure S3. **FTO was a critical target for FTO-IT1 regulating glycolysis. A. qRT-PCR (left) and Western blot (right) showed the relative levels of FTO in normal liver cell line (MIHA) and HCC cell lines. B. GSEA analyses of FTO-correlated genes in HCC tissues from TCGA database. C. The mRNA (left) and protein (right) level of FTO after overexpressing FTO-IT1. D. The knockdown and overexpression efficiency of FTO were detected in HCC cells. E. The glucose uptake (left), lactate production (middle) and pH value (right) in cell medium of Huh7 cells transfected with empty vector or overexpression FTO plasmid. F-H. Colony formation assays (F), EdU assays (G) and MTT assays (H) depicting the change in viability of Huh7 cells transfected with overexpression FTO plasmid with or without the treatment of 2DG. I-K. The proliferation of Huh7 cells transfected with empty vector, overexpression FTO-IT1 plasmid and siFTO was evaluated by colony formation assays (I), EdU assays (J) and MTT assays (K). **Supplementary Figure S4. **FTO regulated the invasion, metastasis and apoptosis of HCC cells. A. Wound-healing assay was performed to evaluate the migration ability of HCC cells transfected with siNC or siFTO. Representative images of three time points (0 h, 24 h) after would scratching of cells were shown. The result displayed that the wound healing was smaller in siFTO group than siNC group. B. Transwell assay was performed to evaluate the invasion ability of HCC cells transfected with siNC or siFTO. Representative images of invaded cells stained with crystal violet were shown. The result displayed that the invaded cells was less in siFTO group than siNC group. C. Apoptosis of HCC cells transfected with siNC or siFTO were analyzed by flow cytometry. Q2, terminal apoptotic cells; Q3, early apoptotic cells. The result displayed that the number of apoptosis cells was more in siFTO group than siNC group. D. Wound-healing assay was performed to evaluate the migration ability of Huh7 cells transfected with oeVector or oeFTO. Representative images of three time points (0 h, 24 h) after would scratching of cells were shown. The result displayed that the wound healing was bigger in oeFTO group than oeVector group. E. Transwell assay was performed to evaluate the invasion ability of Huh7 cells transfected with oeVector or oeFTO. Representative images of invaded cells stained with crystal violet were shown. The result displayed that the invaded cells was more in oeFTO group than oeVector group. F. Apoptosis of Huh7 cells transfected with oeVector or oeFTO were analyzed by flow cytometry. Q2, terminal apoptotic cells; Q3, early apoptotic cells. The result displayed that the number of apoptosis cells did not change significantly in oeFTO group compared to oeVector group. **Supplementary Figure S5. **FTO-IT1 enhanced the interaction between ILF2 and ILF3 protein. A. Biotin-labeled RNA pull-down followed by mass spectrometry showed the one of unique peptides of ILF2 and ILF3. B. RNA interaction profile from *cat*RAPID (http://service.tartaglialab.com/page/catrapid_group) suggested that FTO-IT1 binds to the ILF2/ILF3 protein. C, D. Effects of knockdown of FTO-IT1 on ILF2/ILF3 mRNA (C) and protein (D) levels. E. Effects of overexpression of FTO-IT1 on ILF2/ILF3 mRNA (left) and protein (right) levels. F, G. The knockdown efficiency of ILF2 (F) and ILF3 (G) were detected in HCC cells. H. Effects of knockdown of ILF2/ILF3 on FTO-IT1. I. The overexpression efficiency of ILF2 (left) and ILF3 (right) were detected in HCC cells. J. Effects of overexpression of ILF2/ILF3 on FTO-IT1. **Supplementary Figure S6. **FTO-IT1 stabilized FTO mRNA by strengthening the binding between ILF2/ILF3 complex and FTO mRNA. A. The mRNA (left) and protein (right) levels of FTO in ILF2 overexpression or ILF3 overexpression Huh7 cells compared with control. B. HepG2/MHCC97H cells were transfected with wild-type pGL3 FTO promotor vector and then treated with siNC or siFTO-IT1. After 48 hours, firefly luciferase activity was detected. C. Huh7 cells were transfected with wild-type pGL3 FTO promotor vector and then treated with empty vector or overexpression FTO-IT1 plasmid. After 48 hours, firefly luciferase activity was detected. D. Huh7 cells transfected with empty vector, overexpression FTO-IT1 plasmid, and then treated with ActD for 0, 2, 4, 6 and 8 hours, respectively, followed by qRT-PCR assays for FTO mRNA. E. Western blot of ILF2/ILF3 pulled down by 3’UTR of FTO or antisense (3’UTR-AS) in Huh7 cells transfected with empty vector or overexpression FTO-IT1 plasmid. F. RIP assay was applied using the anti-ILF2/ILF3 antibody and IgG antibody in HepG2 cells. qRT-PCR was used to detect the corresponding enrichment of 3’UTR of FTO mRNA. G. RIP assay was applied using the anti-ILF2/ILF3 antibody and IgG antibody in Huh7 cells transfected with empty vector or overexpression FTO-IT1 plasmid. H. Huh7 cells transfected with empty vector or overexpression FTO-IT1 plasmid, or co-transfected with siILF2 or siILF3 and treated with ActD for corresponding hours followed by qRT-PCR assays for FTO mRNA. I. The mRNA (left) and protein (right) levels of FTO in Huh7 cells transfected with empty vector or overexpression FTO-IT1 plasmid, or co-transfected with siILF2 or siILF3. **Supplementary Figure S7. **FTO facilitated glycolysis in HCC cells by targeting glycolytic enzymes. A-E. The correlation of the expression between FTO and ALDOA (A), ENO1 (B), PFKFB4 (C), PFKP (D) or PGK1 (E) (left). The relative transcript levels of ALDOA (A), ENO1 (B), PFKFB4 (C), PFKP (D) or PGK1 (E) in HCC tissues with different status of tumor stage (middle). Kaplan-Meier analyses of OS in HCC patients with low and high levels of ALDOA (A), ENO1 (B), PFKFB4 (C), PFKP (D) or PGK1 (E) (right) from GEPIA online database. **Supplementary Figure S8. **FTO-mediated demethylation inhibited YTHDF2-mediated mRNA degradation of GLUT1 and PKM2. A. RIP assay was applied using the anti-YTHDF2 antibody and IgG antibody in HepG2 cells. qRT-PCR was used to detect the corresponding enrichment of GLUT1 and PKM2 mRNA. B. RIP assay was applied using the anti-YTHDF2 antibody and IgG antibody in HepG2 cells transfected with siNC or siFTO. qRT-PCR was used to detect the corresponding enrichment of GLUT1 and PKM2 mRNA. C. RIP assay was applied using the anti-YTHDF2 antibody and IgG antibody in Huh7 cells transfected with empty vector or overexpression FTO plasmid. qRT-PCR was used to detect the corresponding enrichment of GLUT1 and PKM2 mRNA. D. The knockdown and overexpression efficiency of YTHDF2 were detected in HCC cells. E. HepG2 cells transfected with siNC, siFTO, or co-transfected with siYTHDF2 and then treated with ActD for corresponding hours followed by qRT-PCR assays for GLUT1 and PKM2 mRNA. F. The mRNA (left) and protein (right) levels of GLUT1 and PKM2 in HepG2 cells transfected with siNC, siFTO, or co-transfected with siYTHDF2. G. Huh7 cells transfected with empty vector, overexpression FTO plasmid, or co-transfected with overexpression YTHDF2 plasmid and then treated with ActD for corresponding hours followed by qRT-PCR assays for GLUT1 and PKM2 mRNA. H. The mRNA (left) and protein (right) levels of GLUT1 and PKM2 in Huh7 cells transfected with empty vector, overexpression FTO plasmid, or co-transfected with overexpression YTHDF2 plasmid. **Supplementary Figure S9. **c-Myc was reciprocally regulated by FTO-IT1 via FTO-mediated m^6^A demethylation. A. Cells were cultured with sequentially decreased concentration of glucose conditions (25, 10, 5, 2.5, 1, 0.5 mmol/L), then the mRNA (left) and protein (right) levels of FTO level were evaluated. B. The knockdown and overexpression efficiency of c-Myc were detected in HCC cells. C, D. Effects of knockdown (C) and overexpression (D) of FTO-IT1 on c-Myc mRNA and protein levels. E. Pie charts analysis showing the m^6^A modification sites of all sources of c-Myc from the RMvar online database. F. MeRIP assays were applied using the anti-m^6^A antibody and IgG antibody in HepG2 cells. qRT-PCR was used to detect the corresponding enrichment of c-Myc. G. MeRIP assay was applied using the anti-m^6^A antibody and IgG antibody in HepG2 cells transfected with siNC, siFTO-IT1, or co-transfected with overexpression FTO plasmid. qRT-PCR was used to detect the corresponding enrichment of c-Myc. H. MeRIP assay was applied using the anti-m^6^A antibody and IgG antibody in Huh7 cells transfected with empty vector, overexpression FTO plasmid, or co-transfected with siFTO. qRT-PCR was used to detect the corresponding enrichment of c-Myc. I. HepG2 cells transfected with siNC, siFTO-IT1, or co-transfected with overexpression FTO plasmid and then treated with ActD for corresponding hours followed by qRT-PCR assays for c-Myc mRNA. J. Huh7 cells transfected with empty vector, overexpression FTO-IT1 plasmid, or co-transfected with siFTO and then treated with ActD for corresponding hours followed by qRT-PCR assays for c-Myc mRNA. **Supplementary Figure S10. **FTO-IT1/FTO signaling promoted proliferation of HCC cells *in vivo* and correlated with poor clinical outcomes. A-C. Subcutaneous implantation mouse models were established by using Huh7 cells that were transfected with lentivirus containing overexpression FTO-IT1 plasmid and/or siFTO plasmid. Growth curve (A), tumor weight (B) representative images (C) of xenografts (*n* = 5) in the three treatment groups for 28 days were shown. D. Representative HE and IHC staining for FTO, GLUT1, PKM2 and Ki67 expression in the subcutaneous xenografts. The xenografts were collected 4 weeks after tumor implantation. E, F. Orthotopic implantation mouse models were established by using Huh7 cells transfected with lentivirus containing overexpression FTO-IT1 plasmid and/or siFTO plasmid. Representative images (E), tumor size and weight (F) of xenografts (*n* = 4) in the three treatment groups for 28 days were shown. G. Representative HE and IHC staining for FTO, GLUT1, PKM2 and Ki67 expression in the orthotopic xenografts. The xenografts were collected 4 weeks after tumor implantation. H. Three representative IHC images of FTO in 92 pairs HCC clinical specimens and matched paracancerous tissues. Scale bar, 100/25 μm. I. Correlation between FTO-IT1 and FTO, GLUT1, PKM2, c-Myc in specimens of 92 patients with HCC was analyzed by the *Chi*-square test. J. Correlation between FTO and GLUT1, PKM2, c-Myc in specimens of 92 patients with HCC was analyzed by the *Chi*-square test.

**Additional file 2.** Supplementary methods.

**Additional file 3:** **Supplementary Table S1. **The sequence of PCR primers. **Supplementary Table S2. **The sequence of siRNA. **Supplementary Table S3. **The information of antibodies used. **Supplementary Table S4.** Mass spectrometry data of proteins and peptides pulled down by FTO-IT1. **Supplementary Table S5. **The 63 glycolysis-related genes. **Supplementary Table S6. **Three putative c-Myc binding sites of FTO-IT1 promoter using JASPAR database. **Supplementary Table S7. **Clinical information and relative expression of mRNA of 92 patients with HCC. **Supplementary Table S8. **Correlation between FTO-IT1 and clinical characteristics of 92 patients with HCC.

## Data Availability

The datasets used and/or analyzed during the current study are available from the corresponding author on reasonable request.

## Abbreviations

LncRNA: Long non-coding RNA
HCC: Hepatocellular carcinoma
FTO: Fat mass and obesity-associated
FTO-IT1: FTO Intronic Transcript 1
ILF2: Interleukin enhancer binding factor
ILF3: Interleukin enhancer binding factor 3
m^6^A: *N*^6^-Methyladenosine
c-Myc: MYC proto-oncogene
YTHDF2: YTH N6-methyladenosine RNA binding protein 2
GEO: Gene Expression Omnibus
TCGA: The Cancer Genome Atlas
RNA-FISH: RNA fluorescence in situ hybridization
Co-IP: Co-immunoprecipitation
ChIP: Chromatin immunoprecipitation
IHC: Immunohistochemistry
RIP: RNA immunoprecipitation
MeRIP: Methylated RNA immunoprecipitation
qRT-PR: Quantitative reverse transcription PCR
OS: Overall survival
DFS: Disease-free survival
SD: Standard deviation
GSEA: Gene Set Enrichment Analysis
GO: Gene ontology
KEGG: Kyoto Encyclopedia of Genes and Genomes
ActD: Actinomycin D
AREs: AU-rich elements
WT: Wild type
MUT: Mutant
NT: Non-tumor

## References

[1] Vander Heiden MG, Cantley LC, Thompson CB (2009). Understanding the Warburg effect: the metabolic requirements of cell proliferation. Science.

[2] Feng J, Li J, Wu L, Yu Q, Ji J, Wu J (2020). Emerging roles and the regulation of aerobic glycolysis in hepatocellular carcinoma. J Exp Clin Cancer Res.

[3] Luo X, Zheng E, Wei L, Zeng H, Qin H, Zhang X (2021). The fatty acid receptor CD36 promotes HCC progression through activating Src/PI3K/AKT axis-dependent aerobic glycolysis. Cell Death Dis.

[4] Bi L, Ren Y, Feng M, Meng P, Wang Q, Chen W (2021). HDAC11 Regulates Glycolysis through the LKB1/AMPK Signaling Pathway to Maintain Hepatocellular Carcinoma Stemness. Cancer Res.

[5] Jiang L, Zhao L, Bi J, Guan Q, Qi A, Wei Q (2019). Glycolysis gene expression profilings screen for prognostic risk signature of hepatocellular carcinoma. Aging (Albany NY).

[6] Quinn JJ, Chang HY (2016). Unique features of long non-coding RNA biogenesis and function. Nat Rev Genet.

[7] Liao M, Liao W, Xu N, Li B, Liu F, Zhang S (2019). LncRNA EPB41L4A-AS1 regulates glycolysis and glutaminolysis by mediating nucleolar translocation of HDAC2. EBioMedicine.

[8] Park MK, Zhang L, Min KW, Cho JH, Yeh CC, Moon H (2021). NEAT1 is essential for metabolic changes that promote breast cancer growth and metastasis. Cell Metab.

[9] Liu J, Liu ZX, Wu QN, Lu YX, Wong CW, Miao L (2020). Long noncoding RNA AGPG regulates PFKFB3-mediated tumor glycolytic reprogramming. Nat Commun.

[10] Kuwano Y, Pullmann R, Marasa BS, Abdelmohsen K, Lee EK, Yang X (2010). NF90 selectively represses the translation of target mRNAs bearing an AU-rich signature motif. Nucleic Acids Res.

[11] Li K, Wu JL, Qin B, Fan Z, Tang Q, Lu W (2020). ILF3 is a substrate of SPOP for regulating serine biosynthesis in colorectal cancer. Cell Res.

[12] Guarnerio J, Zhang Y, Cheloni G, Panella R, Mae Katon J, Simpson M (2019). Intragenic antagonistic roles of protein and circRNA in tumorigenesis. Cell Res.

[13] Wen X, Liu X, Mao YP, Yang XJ, Wang YQ, Zhang PP (2018). Long non-coding RNA DANCR stabilizes HIF-1α and promotes metastasis by interacting with NF90/NF45 complex in nasopharyngeal carcinoma. Theranostics.

[14] Surka C, Jin L, Mbong N, Lu CC, Jang IS, Rychak E (2021). CC-90009, a novel cereblon E3 ligase modulator, targets acute myeloid leukemia blasts and leukemia stem cells. Blood.

[15] Castella S, Bernard R, Corno M, Fradin A, Larcher JC (2015). Ilf3 and NF90 functions in RNA biology. Wiley Interdiscip Rev RNA.

[16] Kiesler P, Haynes PA, Shi L, Kao PN, Wysocki VH, Vercelli D (2010). NF45 and NF90 regulate HS4-dependent interleukin-13 transcription in T cells. J Biol Chem.

[17] Su R, Dong L, Li Y, Gao M, Han L, Wunderlich M (2020). Targeting FTO Suppresses Cancer Stem Cell Maintenance and Immune Evasion. Cancer Cell.

[18] Niu Y, Lin Z, Wan A, Chen H, Liang H, Sun L (2019). RNA N6-methyladenosine demethylase FTO promotes breast tumor progression through inhibiting BNIP3. Mol Cancer.

[19] Dominissini D, Moshitch-Moshkovitz S, Schwartz S, Salmon-Divon M, Ungar L, Osenberg S (2012). Topology of the human and mouse m6A RNA methylomes revealed by m6A-seq. Nature.

[20] Wang X, Lu Z, Gomez A, Hon GC, Yue Y, Han D (2014). N6-methyladenosine-dependent regulation of messenger RNA stability. Nature.

[21] Dejure FR, Eilers M (2017). MYC and tumor metabolism: chicken and egg. Embo j.

[22] Wu S, Yin X, Fang X, Zheng J, Li L, Liu X (2015). c-MYC responds to glucose deprivation in a cell-type-dependent manner. Cell Death Discov.

[23] Su R, Dong L, Li C, Nachtergaele S, Wunderlich M, Qing Y (2018). R-2HG Exhibits Anti-tumor Activity by Targeting FTO/m(6)A/MYC/CEBPA Signaling. Cell.

[24] Hanahan D, Weinberg RA (2011). Hallmarks of cancer: the next generation. Cell.

[25] Tang J, Yan T, Bao Y, Shen C, Yu C, Zhu X (2019). LncRNA GLCC1 promotes colorectal carcinogenesis and glucose metabolism by stabilizing c-Myc. Nat Commun.

[26] Hu Y, Tang J, Xu F, Chen J, Zeng Z, Han S (2022). A reciprocal feedback between N6-methyladenosine reader YTHDF3 and lncRNA DICER1-AS1 promotes glycolysis of pancreatic cancer through inhibiting maturation of miR-5586-5p. J Exp Clin Cancer Res.

[27] Wight M, Werner A (2013). The functions of natural antisense transcripts. Essays Biochem.

[28] Hu Y, Wang F, Xu F, Fang K, Fang Z, Shuai X (2020). A reciprocal feedback of Myc and lncRNA MTSS1-AS contributes to extracellular acidity-promoted metastasis of pancreatic cancer. Theranostics.

[29] Sun L, Wang L, Chen T, Shi Y, Yao B, Liu Z (2020). LncRNA RUNX1-IT1 which is downregulated by hypoxia-driven histone deacetylase 3 represses proliferation and cancer stem-like properties in hepatocellular carcinoma cells. Cell Death Dis.

[30] Wang TH, Yu CC, Lin YS, Chen TC, Yeh CT, Liang KH (2016). Long noncoding RNA CPS1-IT1 suppresses the metastasis of hepatocellular carcinoma by regulating HIF-1α activity and inhibiting epithelial-mesenchymal transition. Oncotarget.

[31] Guan D, Altan-Bonnet N, Parrott AM, Arrigo CJ, Li Q, Khaleduzzaman M (2008). Nuclear factor 45 (NF45) is a regulatory subunit of complexes with NF90/110 involved in mitotic control. Mol Cell Biol.

[32] Li W, Han S, Hu P, Chen D, Zeng Z, Hu Y (2021). LncRNA ZNFTR functions as an inhibitor in pancreatic cancer by modulating ATF3/ZNF24/VEGFA pathway. Cell Death Dis.

[33] Xu F, Huang M, Chen Q, Niu Y, Hu Y, Hu P (2021). LncRNA HIF1A-AS1 Promotes Gemcitabine Resistance of Pancreatic Cancer by Enhancing Glycolysis through Modulating the AKT/YB1/HIF1α Pathway. Cancer Res.

[34] Yang S, Wei J, Cui YH, Park G, Shah P, Deng Y (2019). m(6)A mRNA demethylase FTO regulates melanoma tumorigenicity and response to anti-PD-1 blockade. Nat Commun.

[35] Cui Q, Shi H, Ye P, Li L, Qu Q, Sun G (2017). m(6)A RNA Methylation Regulates the Self-Renewal and Tumorigenesis of Glioblastoma Stem Cells. Cell Rep.

[36] Li Z, Weng H, Su R, Weng X, Zuo Z, Li C (2017). FTO Plays an Oncogenic Role in Acute Myeloid Leukemia as a N(6)-Methyladenosine RNA Demethylase. Cancer Cell.

[37] Wang H, Liang Z, Gou Y, Li Z, Cao Y, Jiao N (2022). FTO-dependent N(6)-Methyladenosine regulates the progression of endometriosis via the ATG5/PKM2 Axis. Cell Signal.

[38] Huang J, Sun W, Wang Z, Lv C, Zhang T, Zhang D (2022). FTO suppresses glycolysis and growth of papillary thyroid cancer via decreasing stability of APOE mRNA in an N6-methyladenosine-dependent manner. J Exp Clin Cancer Res.

[39] Wang X, Zhao BS, Roundtree IA, Lu Z, Han D, Ma H (2015). N(6)-methyladenosine Modulates Messenger RNA Translation Efficiency. Cell.

[40] Huang H, Weng H, Sun W, Qin X, Shi H, Wu H (2018). Recognition of RNA N(6)-methyladenosine by IGF2BP proteins enhances mRNA stability and translation. Nat Cell Biol.

[41] Fang R, Chen X, Zhang S, Shi H, Ye Y, Shi H (2021). EGFR/SRC/ERK-stabilized YTHDF2 promotes cholesterol dysregulation and invasive growth of glioblastoma. Nat Commun.

[42] Deng SJ, Chen HY, Zeng Z, Deng S, Zhu S, Ye Z (2019). Nutrient Stress-Dysregulated Antisense lncRNA GLS-AS Impairs GLS-Mediated Metabolism and Represses Pancreatic Cancer Progression. Cancer Res.

[43] Yue C, Chen J, Li Z, Li L, Chen J, Guo Y (2020). microRNA-96 promotes occurrence and progression of colorectal cancer via regulation of the AMPKα2-FTO-m6A/MYC axis. J Exp Clin Cancer Res.

[44] Yang Z, Jiang X, Zhang Z, Zhao Z, Xing W, Liu Y (2021). HDAC3-dependent transcriptional repression of FOXA2 regulates FTO/m6A/MYC signaling to contribute to the development of gastric cancer. Cancer Gene Ther.

[45] Xiao L, Li X, Mu Z, Zhou J, Zhou P, Xie C (2020). FTO Inhibition Enhances the Antitumor Effect of Temozolomide by Targeting MYC-miR-155/23a Cluster-MXI1 Feedback Circuit in Glioma. Cancer Res.

[46] Yang X, Shao F, Guo D, Wang W, Wang J, Zhu R (2021). WNT/β-catenin-suppressed FTO expression increases m(6)A of c-Myc mRNA to promote tumor cell glycolysis and tumorigenesis. Cell Death Dis.

