# Supplementary material for: LncRNA FTO-IT1 promotes glycolysis and progression of hepatocellular carcinoma through modulating FTO-mediated N6-methyladenosine modification on GLUT1 and PKM2
Source: J Exp Clin Cancer Res. 2023 Oct 16;42:267. doi: 10.1186/s13046-023-02847-2 (PMC10578010; doi:10.1186/s13046-023-02847-2)
Supplement: Supplementary file 1 — Additional file 1: Supplementary Figure S1. FTO-IT1 was a lncRNA upregulated in HCC samples and associated with poor prognosis. A, B. GO (A) and KEGG (B) analyses of FTO-IT1-correlated genes in HCC tissues from TCGA database. C. The sequence of FTO-IT1 transcript from the Nucleotide database in NCBI. D. Predicted secondary structure of FTO-IT1 from the RNAfold web server (http://rna.tbi.univie.ac.at/cgi-bin/RNAWebSuite/RNAfold.cgi). E-F. The Coding potentials of FTO-IT1 were evaluated using CPC2 (E, http://cpc2.cbi.pku.edu.cn) and PyhloCSF (F). Supplementary Figure S2. FTO-IT1 promoted glycolysis and proliferation of HCC cells. A. Overexpression efficiency of pcDNA-FTO-IT1 plasmid was assessed via transfection assay followed by qRT-PCR. B. The glucose uptake (left), lactate production (middle) and pH value (right) of HCC cells transfected with empty vector or overexpression FTO-IT1 plasmid with or without the treatment of 2DG. C-E. Colony formation assays (C), EdU assays (D) and MTT assays (E) depicting the change in viability of Huh7 cells transfected with overexpression FTO-IT1 plasmid with or without the treatment of 2DG. Supplementary Figure S3. FTO was a critical target for FTO-IT1 regulating glycolysis. A. qRT-PCR (left) and Western blot (right) showed the relative levels of FTO in normal liver cell line (MIHA) and HCC cell lines. B. GSEA analyses of FTO-correlated genes in HCC tissues from TCGA database. C. The mRNA (left) and protein (right) level of FTO after overexpressing FTO-IT1. D. The knockdown and overexpression efficiency of FTO were detected in HCC cells. E. The glucose uptake (left), lactate production (middle) and pH value (right) in cell medium of Huh7 cells transfected with empty vector or overexpression FTO plasmid. F-H. Colony formation assays (F), EdU assays (G) and MTT assays (H) depicting the change in viability of Huh7 cells transfected with overexpression FTO plasmid with or without the treatment of 2DG. I-K. The proliferation of Huh7 cel [file 13046_2023_2847_MOESM1_ESM.docx]

**LncRNA FTO-IT1 promotes glycolysis and progression of hepatocellular carcinoma through modulating FTO-mediated N6-methyladenosine modification on GLUT1 and PKM2**

**
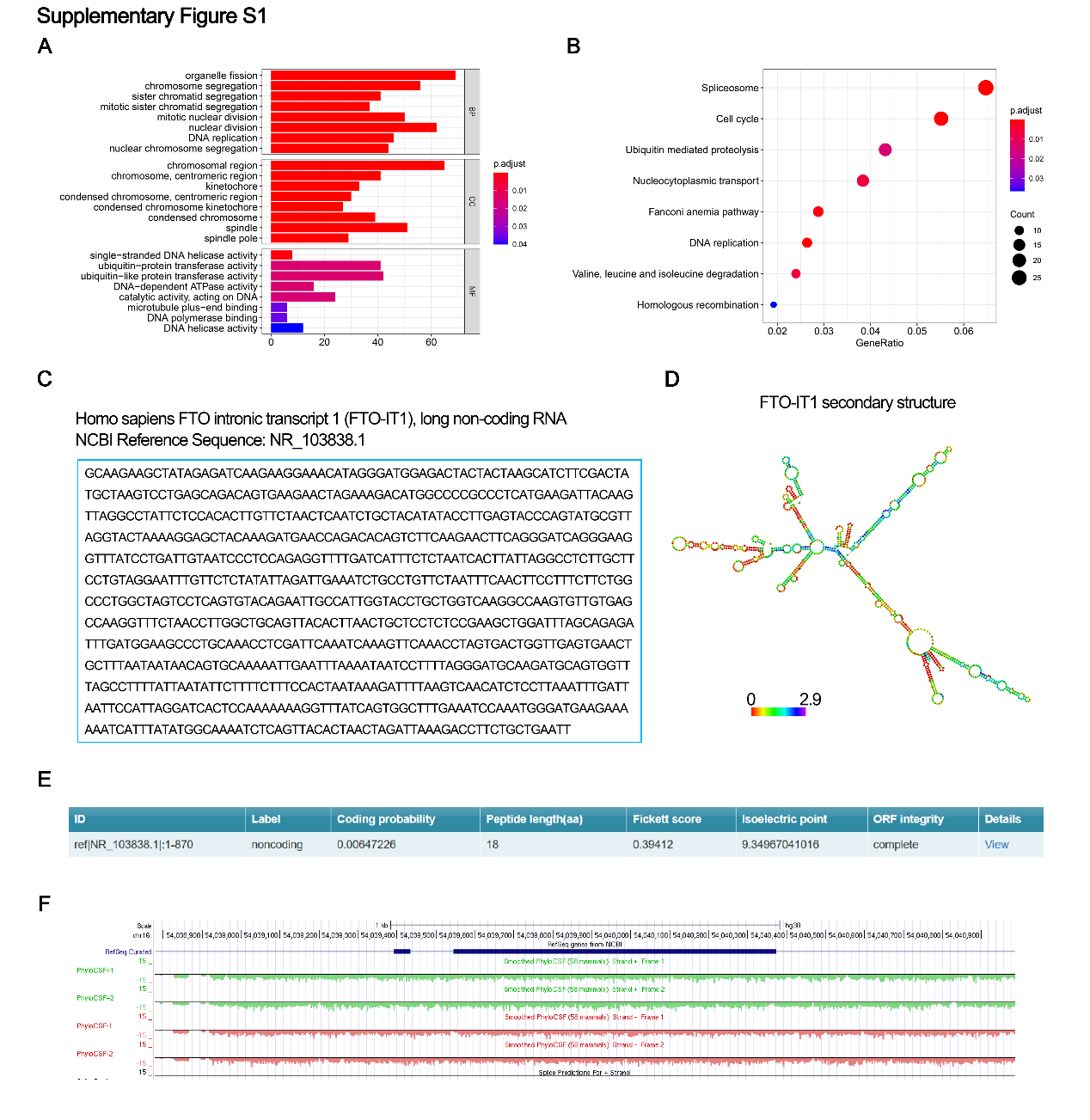
Supplementary Figure S1.** **FTO-IT1 was a lncRNA upregulated in HCC samples and associated with poor prognosis. A, B.** GO (**A**) and KEGG (**B**) analyses of FTO-IT1-correlated genes in HCC tissues from TCGA database. **C.** The sequence of FTO-IT1 transcript from the Nucleotide database in NCBI. **D.** Predicted secondary structure of FTO-IT1 from the RNAfold web server (http://rna.tbi.univie.ac.at/cgi-bin/RNAWebSuite/RNAfold.cgi). **E-F.** The Coding potentials of FTO-IT1 were evaluated using CPC2 (**E**, http://cpc2.cbi.pku.edu.cn) and PyhloCSF (**F**).


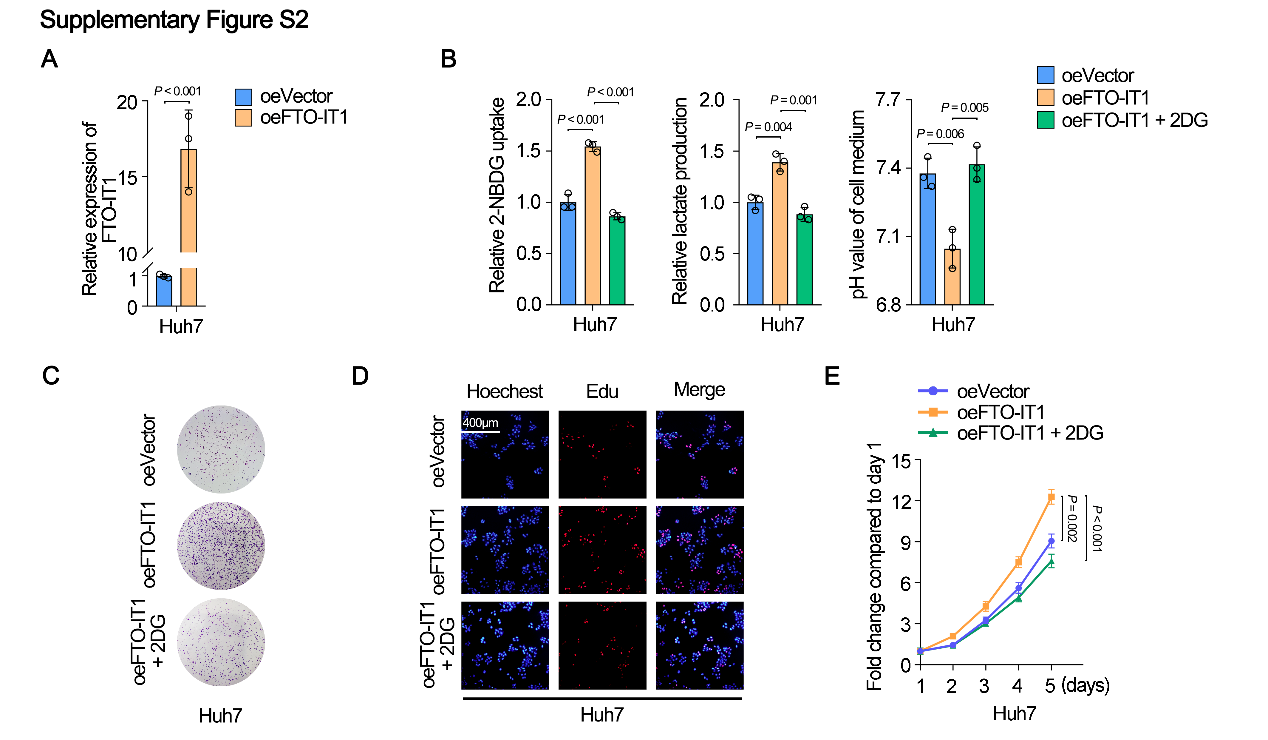


**Supplementary Figure S2.** **FTO-IT1 promoted glycolysis and proliferation of HCC cells. A.** Overexpression efficiency of pcDNA-FTO-IT1 plasmid was assessed via transfection assay followed by qRT-PCR. **B.** The glucose uptake (left), lactate production (middle) and pH value (right) of HCC cells transfected with empty vector or overexpression FTO-IT1 plasmid with or without the treatment of 2DG. **C-E.** Colony formation assays (**C**), EdU assays (**D**) and MTT assays (**E**) depicting the change in viability of Huh7 cells transfected with overexpression FTO-IT1 plasmid with or without the treatment of 2DG.


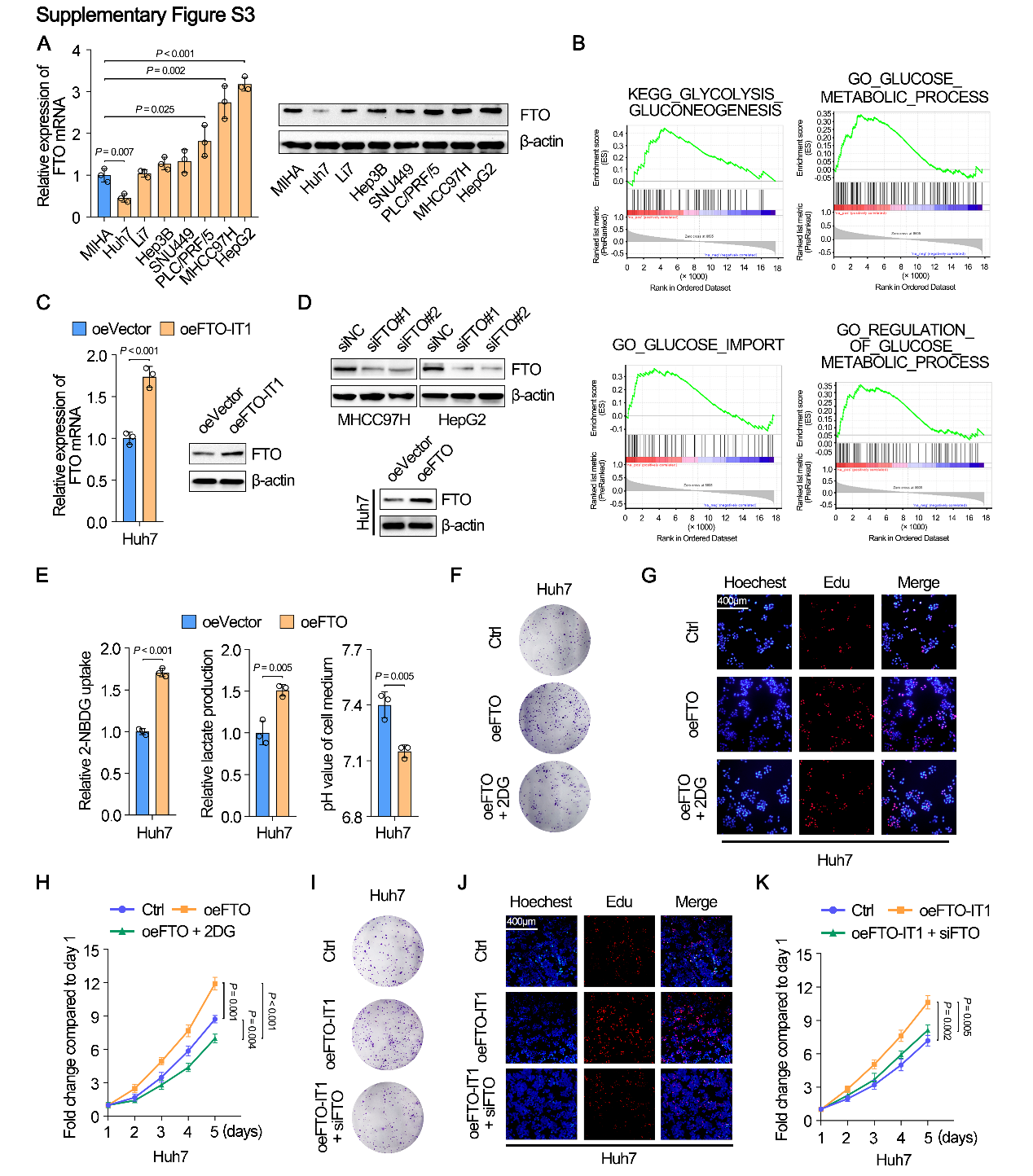


**Supplementary Figure S3. FTO was a critical target for FTO-IT1 regulating glycolysis. A.** qRT-PCR (left) and Western blot (right) showed the relative levels of FTO in normal liver cell line (MIHA) and HCC cell lines. **B.** GSEA analyses of FTO-correlated genes in HCC tissues from TCGA database. **C.** The mRNA (left) and protein (right) level of FTO after overexpressing FTO-IT1. **D.** The knockdown and overexpression efficiency of FTO were detected in HCC cells. **E.** The glucose uptake (left), lactate production (middle) and pH value (right) in cell medium of Huh7 cells transfected with empty vector or overexpression FTO plasmid. **F-H.** Colony formation assays (**F**), EdU assays (**G**) and MTT assays (**H**) depicting the change in viability of Huh7 cells transfected with overexpression FTO plasmid with or without the treatment of 2DG. **I-K.** The proliferation of Huh7 cells transfected with empty vector, overexpression FTO-IT1 plasmid and siFTO was evaluated by colony formation assays (**I**), EdU assays (**J**) and MTT assays (**K**).


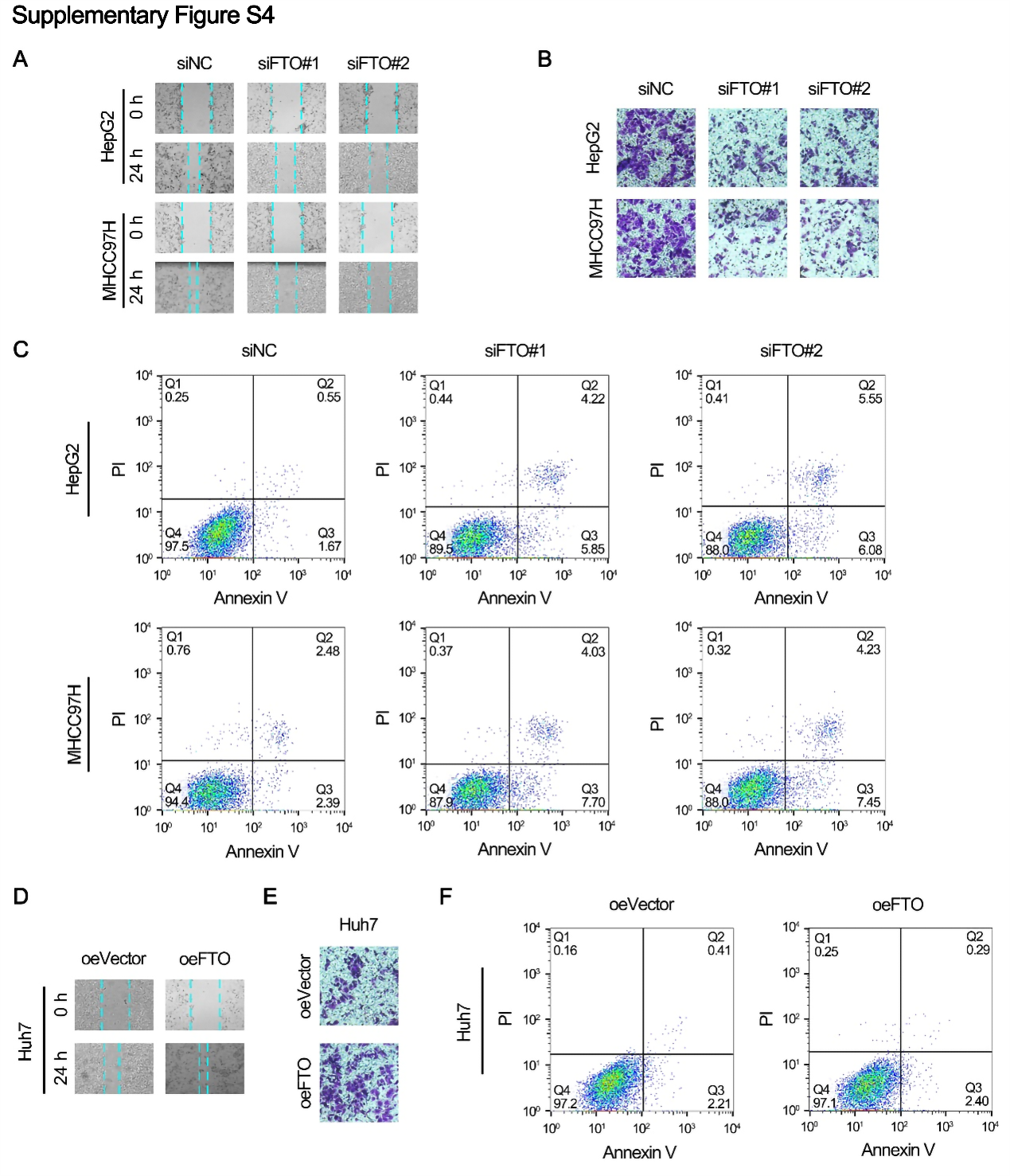


**Supplementary Figure S4. FTO regulated the invasion, metastasis and apoptosis of HCC cells. A.** Wound-healing assay was performed to evaluate the migration ability of HCC cells transfected with siNC or siFTO. Representative images of three time points (0 h, 24 h) after would scratching of cells were shown. The result displayed that the wound healing was smaller in siFTO group than siNC group.

**B.** Transwell assay was performed to evaluate the invasion ability of HCC cells transfected with siNC or siFTO. Representative images of invaded cells stained with crystal violet were shown. The result displayed that the invaded cells was less in siFTO group than siNC group.

**C.** Apoptosis of HCC cells transfected with siNC or siFTO were analyzed by flow cytometry. Q2, terminal apoptotic cells; Q3, early apoptotic cells. The result displayed that the number of apoptosis cells was more in siFTO group than siNC group.

**D.** Wound-healing assay was performed to evaluate the migration ability of Huh7 cells transfected with oeVector or oeFTO. Representative images of three time points (0 h, 24 h) after would scratching of cells were shown. The result displayed that the wound healing was bigger in oeFTO group than oeVector group.

**E.** Transwell assay was performed to evaluate the invasion ability of Huh7 cells transfected with oeVector or oeFTO. Representative images of invaded cells stained with crystal violet were shown. The result displayed that the invaded cells was more in oeFTO group than oeVector group.

**F.** Apoptosis of Huh7 cells transfected with oeVector or oeFTO were analyzed by flow cytometry. Q2, terminal apoptotic cells; Q3, early apoptotic cells. The result displayed that the number of apoptosis cells did not change significantly in oeFTO group compared to oeVector group.


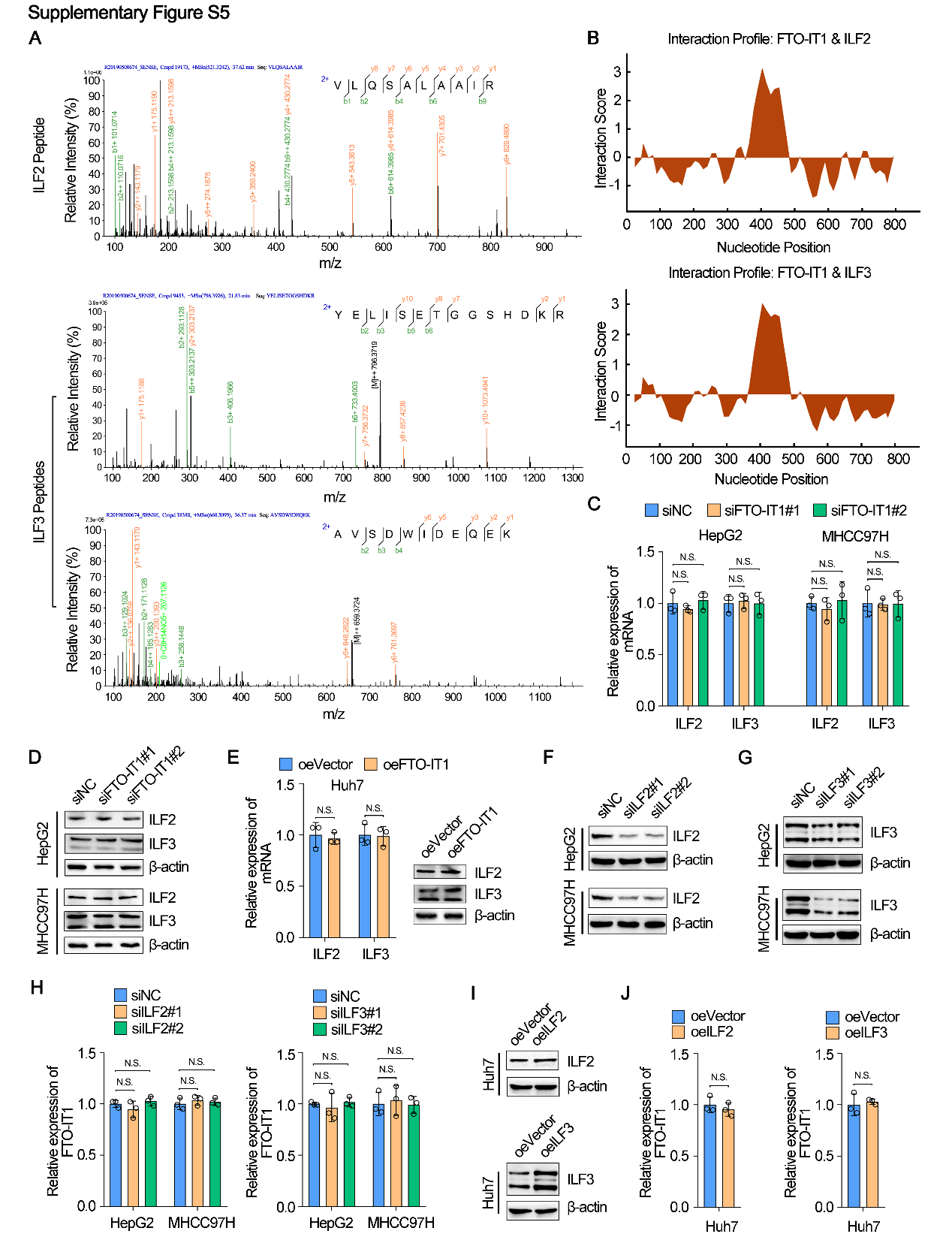


**Supplementary Figure S5. FTO-IT1 enhanced the interaction between ILF2 and ILF3 protein. A.** Biotin-labeled RNA pull-down followed by mass spectrometry showed the one of unique peptides of ILF2 and ILF3. **B.** RNA interaction profile from *cat*RAPID (http://service.tartaglialab.com/page/catrapid_group) suggested that FTO-IT1 binds to the ILF2/ILF3 protein. **C, D.** Effects of knockdown of FTO-IT1 on ILF2/ILF3 mRNA (**C**) and protein (**D**) levels. **E.** Effects of overexpression of FTO-IT1 on ILF2/ILF3 mRNA (left) and protein (right) levels. **F, G.** The knockdown efficiency of ILF2 (**F**) and ILF3 (**G**) were detected in HCC cells. **H.** Effects of knockdown of ILF2/ILF3 on FTO-IT1. **I.** The overexpression efficiency of ILF2 (left) and ILF3 (right) were detected in HCC cells. **J.** Effects of overexpression of ILF2/ILF3 on FTO-IT1.


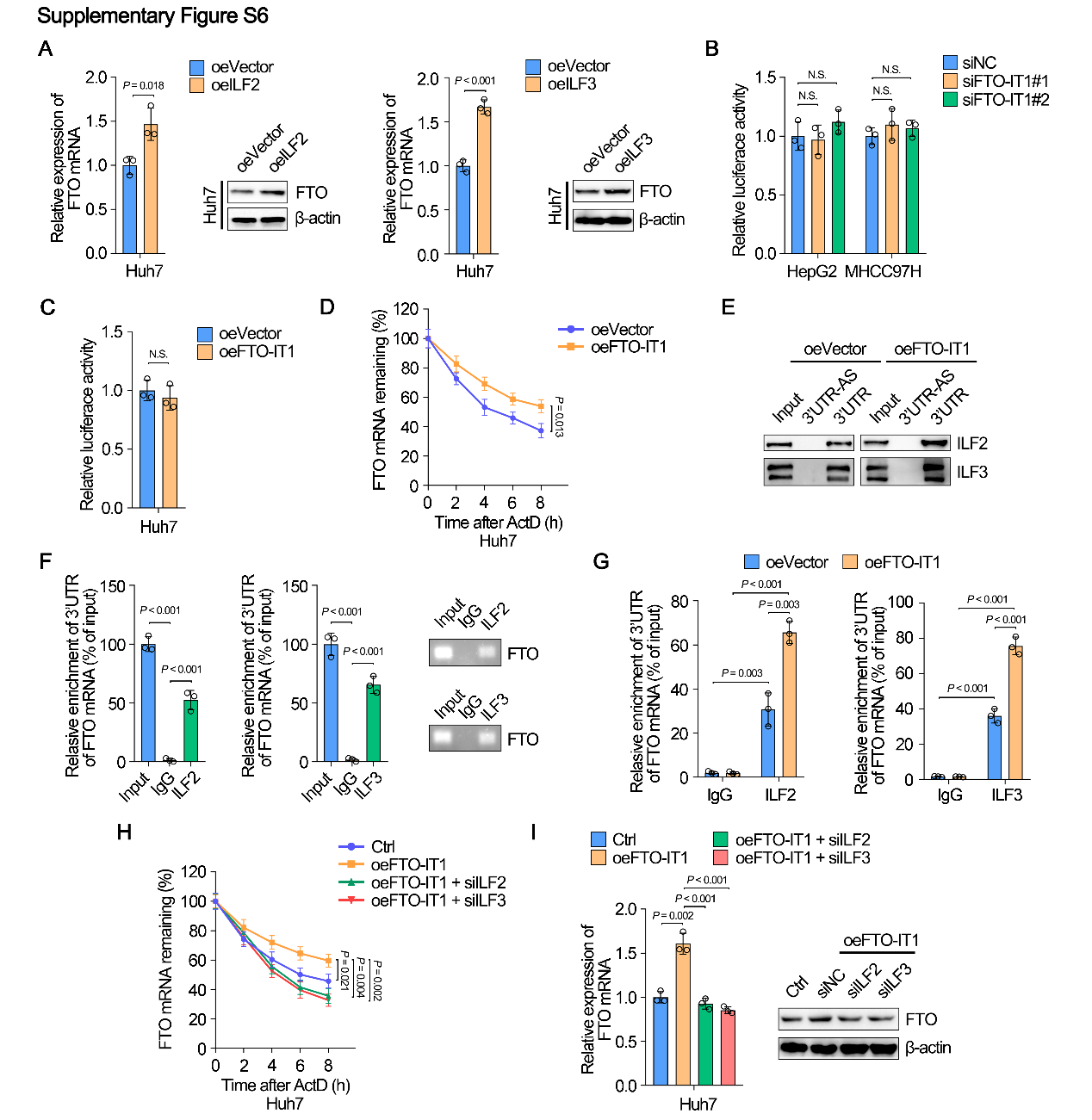


**Supplementary Figure S6. FTO-IT1 stabilized FTO mRNA by strengthening the binding between ILF2/ILF3 complex and FTO mRNA. A.** The mRNA (left) and protein (right) levels of FTO in ILF2 overexpression or ILF3 overexpression Huh7 cells compared with control. **B.** HepG2/MHCC97H cells were transfected with wild-type pGL3 FTO promotor vector and then treated with siNC or siFTO-IT1. After 48 hours, firefly luciferase activity was detected. **C.** Huh7 cells were transfected with wild-type pGL3 FTO promotor vector and then treated with empty vector or overexpression FTO-IT1 plasmid. After 48 hours, firefly luciferase activity was detected. **D.** Huh7 cells transfected with empty vector, overexpression FTO-IT1 plasmid, and then treated with ActD for 0, 2, 4, 6 and 8 hours, respectively, followed by qRT-PCR assays for FTO mRNA. **E.** Western blot of ILF2/ILF3 pulled down by 3’UTR of FTO or antisense (3’UTR-AS) in Huh7 cells transfected with empty vector or overexpression FTO-IT1 plasmid. **F.** RIP assay was applied using the anti-ILF2/ILF3 antibody and IgG antibody in HepG2 cells. qRT-PCR was used to detect the corresponding enrichment of 3’UTR of FTO mRNA. **G.** RIP assay was applied using the anti-ILF2/ILF3 antibody and IgG antibody in Huh7 cells transfected with empty vector or overexpression FTO-IT1 plasmid. **H.** Huh7 cells transfected with empty vector or overexpression FTO-IT1 plasmid, or co-transfected with siILF2 or siILF3 and treated with ActD for corresponding hours followed by qRT-PCR assays for FTO mRNA. **I.** The mRNA (left) and protein (right) levels of FTO in Huh7 cells transfected with empty vector or overexpression FTO-IT1 plasmid, or co-transfected with siILF2 or siILF3.


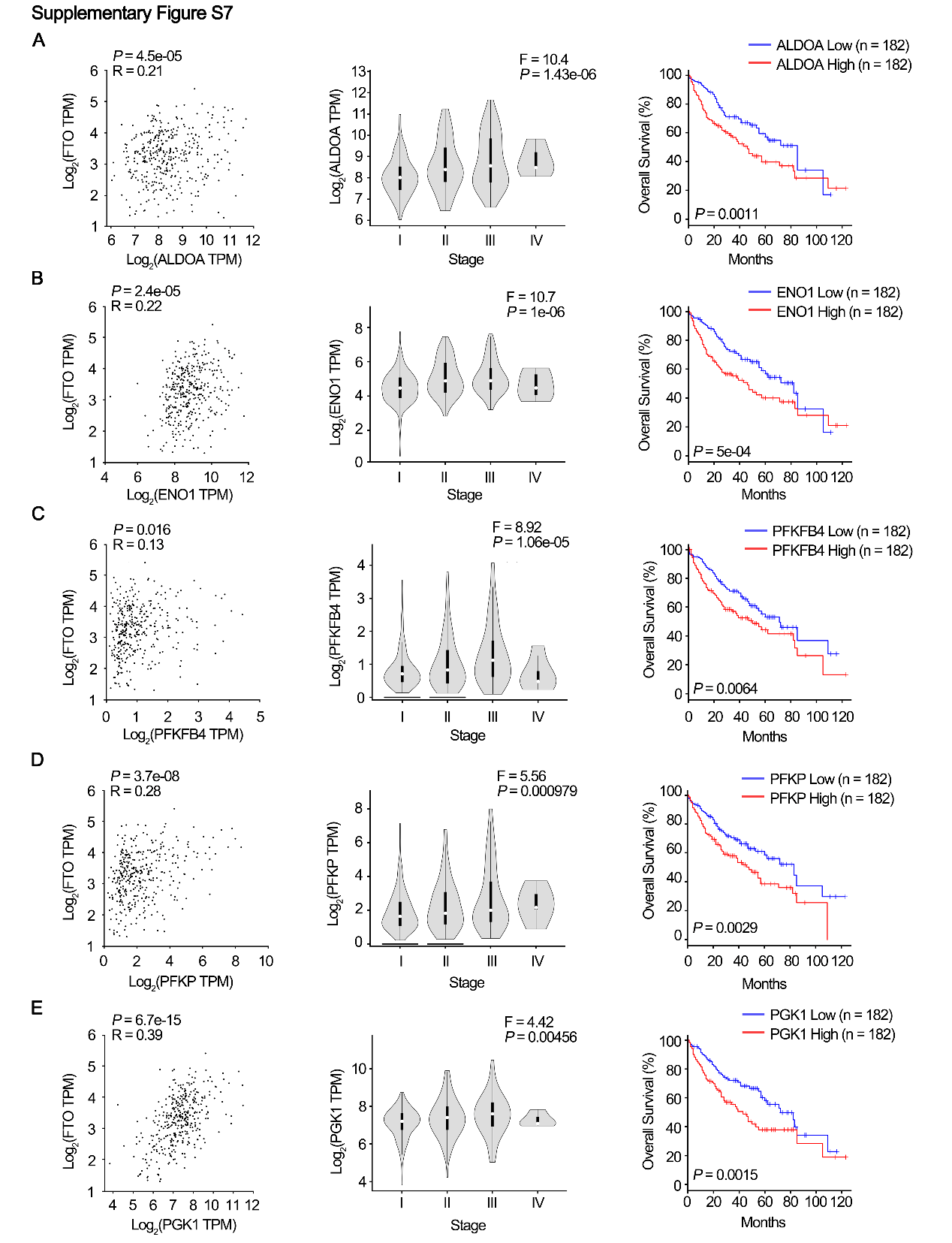


**Supplementary Figure S7. FTO facilitated glycolysis in HCC cells by targeting glycolytic enzymes. A-E.** The correlation of the expression between FTO and ALDOA (**A**), ENO1 (**B**), PFKFB4 (**C**), PFKP (**D**) or PGK1 (**E**) (left). The relative transcript levels of ALDOA (**A**), ENO1 (**B**), PFKFB4 (**C**), PFKP (**D**) or PGK1 (**E**) in HCC tissues with different status of tumor stage (middle). Kaplan-Meier analyses of OS in HCC patients with low and high levels of ALDOA (**A**), ENO1 (**B**), PFKFB4 (**C**), PFKP (**D**) or PGK1 (**E**) (right) from GEPIA online database.


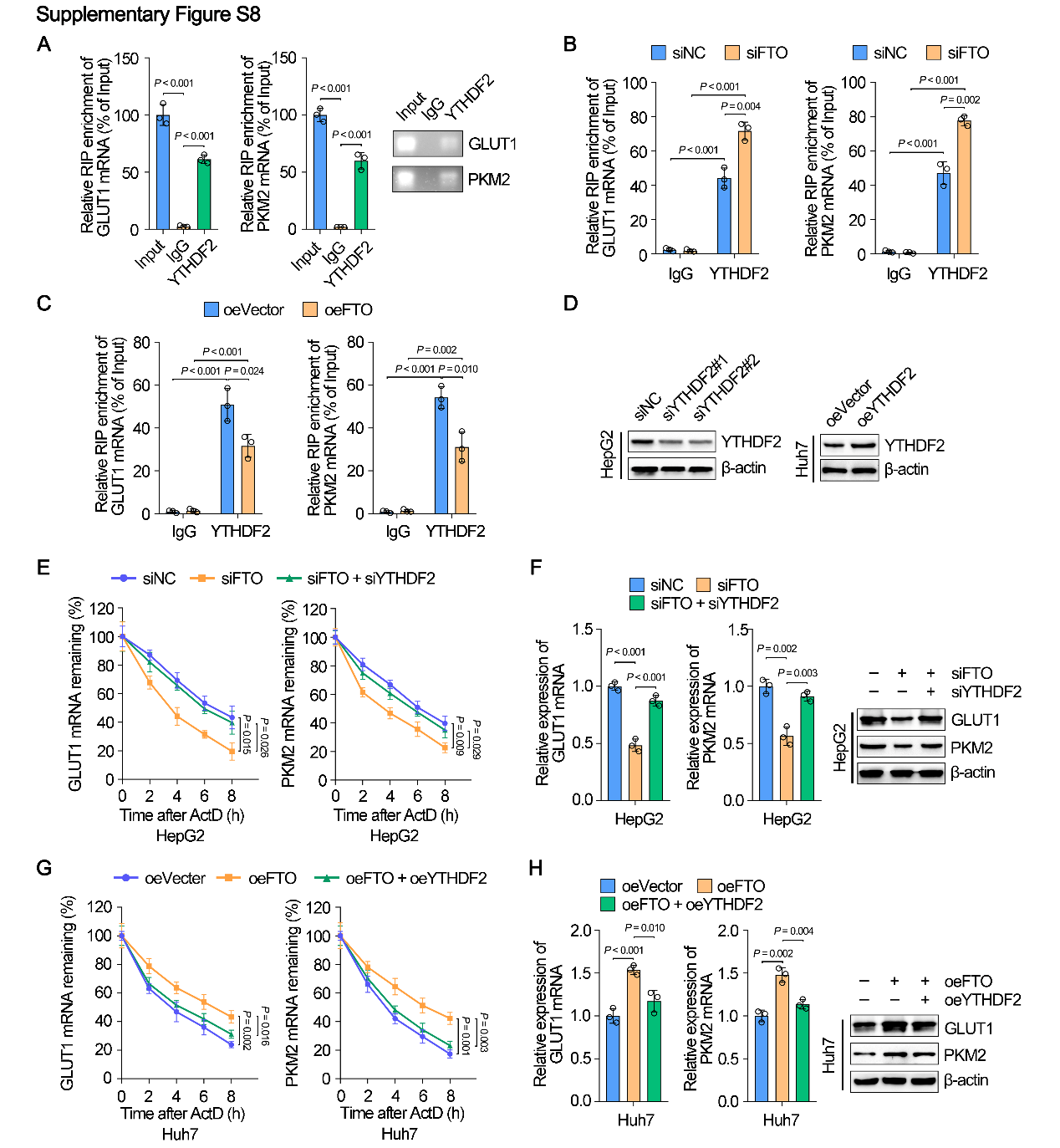


**Supplementary Figure S8. FTO-mediated demethylation inhibited YTHDF2-mediated mRNA degradation of GLUT1 and PKM2. A.** RIP assay was applied using the anti-YTHDF2 antibody and IgG antibody in HepG2 cells. qRT-PCR was used to detect the corresponding enrichment of GLUT1 and PKM2 mRNA. **B.** RIP assay was applied using the anti-YTHDF2 antibody and IgG antibody in HepG2 cells transfected with siNC or siFTO. qRT-PCR was used to detect the corresponding enrichment of GLUT1 and PKM2 mRNA. **C.** RIP assay was applied using the anti-YTHDF2 antibody and IgG antibody in Huh7 cells transfected with empty vector or overexpression FTO plasmid. qRT-PCR was used to detect the corresponding enrichment of GLUT1 and PKM2 mRNA. **D.** The knockdown and overexpression efficiency of YTHDF2 were detected in HCC cells. **E.** HepG2 cells transfected with siNC, siFTO, or co-transfected with siYTHDF2 and then treated with ActD for corresponding hours followed by qRT-PCR assays for GLUT1 and PKM2 mRNA. **F.** The mRNA (left) and protein (right) levels of GLUT1 and PKM2 in HepG2 cells transfected with siNC, siFTO, or co-transfected with siYTHDF2. **G.** Huh7 cells transfected with empty vector, overexpression FTO plasmid, or co-transfected with overexpression YTHDF2 plasmid and then treated with ActD for corresponding hours followed by qRT-PCR assays for GLUT1 and PKM2 mRNA. **H.** The mRNA (left) and protein (right) levels of GLUT1 and PKM2 in Huh7 cells transfected with empty vector, overexpression FTO plasmid, or co-transfected with overexpression YTHDF2 plasmid.


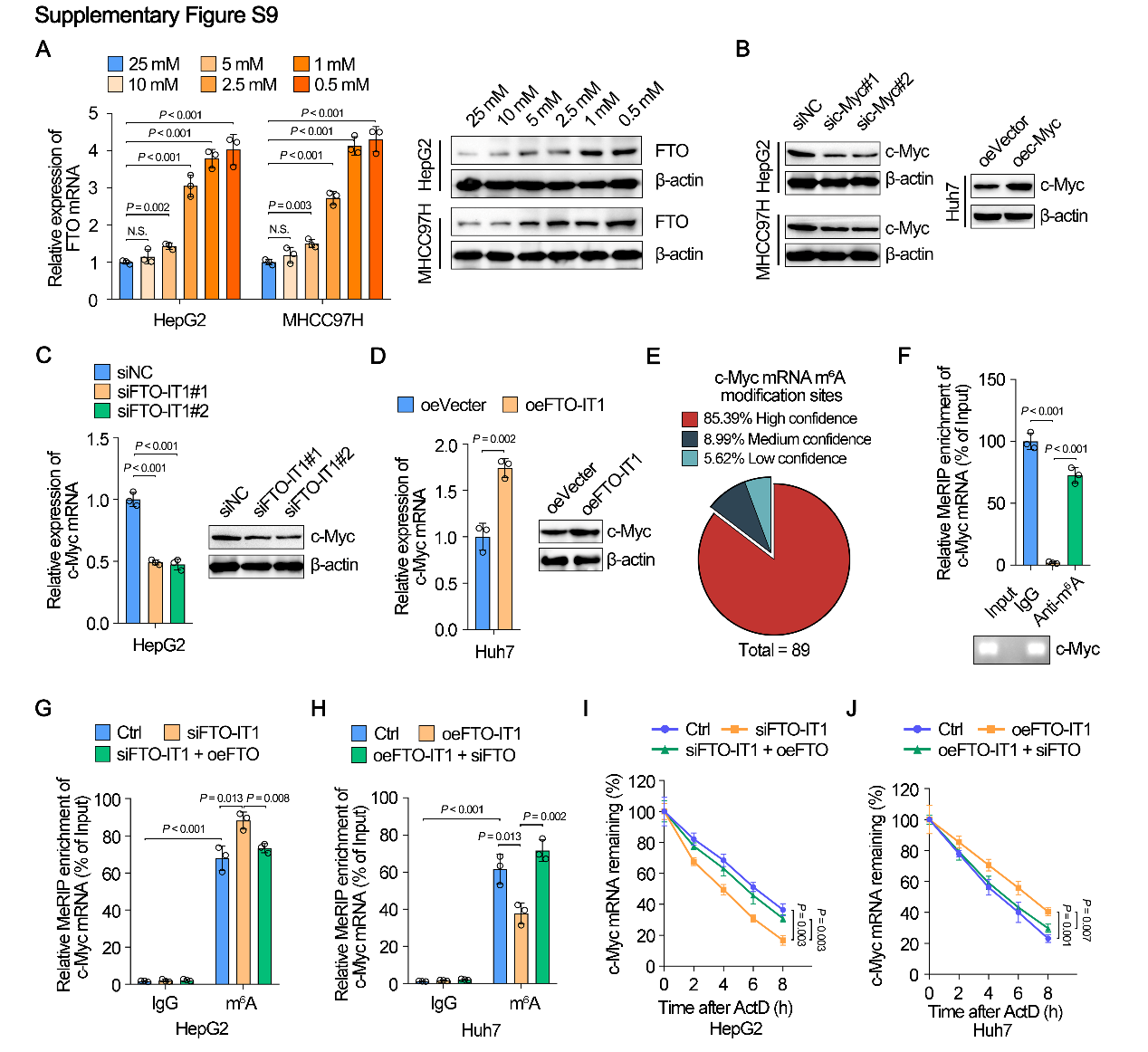


**Supplementary Figure S9. c-Myc was reciprocally regulated by FTO-IT1 via FTO-mediated m^6^A demethylation. A.** Cells were cultured with sequentially decreased concentration of glucose conditions (25, 10, 5, 2.5, 1, 0.5 mmol/L), then the mRNA (left) and protein (right) levels of FTO level were evaluated. **B.** The knockdown and overexpression efficiency of c-Myc were detected in HCC cells. **C, D.** Effects of knockdown (**C**) and overexpression (**D**) of FTO-IT1 on c-Myc mRNA and protein levels. **E.** Pie charts analysis showing the m^6^A modification sites of all sources of c-Myc from the RMvar online database. **F.** MeRIP assays were applied using the anti-m^6^A antibody and IgG antibody in HepG2 cells. qRT-PCR was used to detect the corresponding enrichment of c-Myc. **G.** MeRIP assay was applied using the anti-m^6^A antibody and IgG antibody in HepG2 cells transfected with siNC, siFTO-IT1, or co-transfected with overexpression FTO plasmid. qRT-PCR was used to detect the corresponding enrichment of c-Myc. **H.** MeRIP assay was applied using the anti-m^6^A antibody and IgG antibody in Huh7 cells transfected with empty vector, overexpression FTO plasmid, or co-transfected with siFTO. qRT-PCR was used to detect the corresponding enrichment of c-Myc. **I.** HepG2 cells transfected with siNC, siFTO-IT1, or co-transfected with overexpression FTO plasmid and then treated with ActD for corresponding hours followed by qRT-PCR assays for c-Myc mRNA. **J.** Huh7 cells transfected with empty vector, overexpression FTO-IT1 plasmid, or co-transfected with siFTO and then treated with ActD for corresponding hours followed by qRT-PCR assays for c-Myc mRNA.


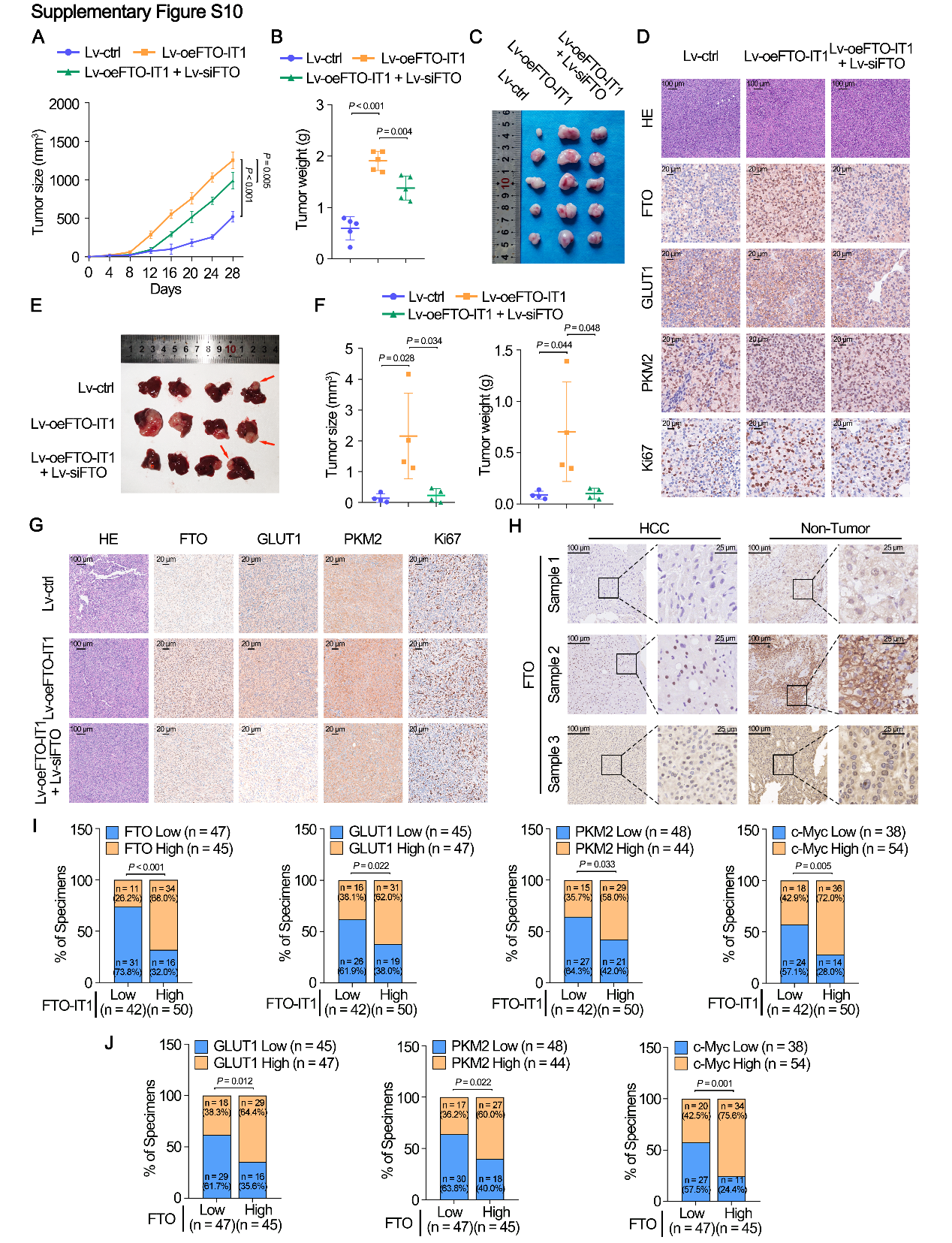


**Supplementary Figure S10. FTO-IT1/FTO signaling promoted proliferation of HCC cells *in vivo* and correlated with poor clinical outcomes. A-C.** Subcutaneous implantation mouse models were established by using Huh7 cells that were transfected with lentivirus containing overexpression FTO-IT1 plasmid and/or siFTO plasmid. Growth curve (**A**), tumor weight (**B**) representative images (**C**) of xenografts (n = 5) in the three treatment groups for 28 days were shown. **D.** Representative HE and IHC staining for FTO, GLUT1, PKM2 and Ki67 expression in the subcutaneous xenografts. The xenografts were collected 4 weeks after tumor implantation. **E, F.** Orthotopic implantation mouse models were established by using Huh7 cells transfected with lentivirus containing overexpression FTO-IT1 plasmid and/or siFTO plasmid. Representative images (**E**), tumor size and weight (**F**) of xenografts (n = 4) in the three treatment groups for 28 days were shown. **G.** Representative HE and IHC staining for FTO, GLUT1, PKM2 and Ki67 expression in the orthotopic xenografts. The xenografts were collected 4 weeks after tumor implantation. **H.** Three representative IHC images of FTO in 92 pairs HCC clinical specimens and matched paracancerous tissues. Scale bar, 100/25 μm. **I.** Correlation between FTO-IT1 and FTO, GLUT1, PKM2, c-Myc in specimens of 92 patients with HCC was analyzed by the *Chi*-square test. **J.** Correlation between FTO and GLUT1, PKM2, c-Myc in specimens of 92 patients with HCC was analyzed by the *Chi*-square test.
