# Supplementary material for: LncRNA FTO-IT1 promotes glycolysis and progression of hepatocellular carcinoma through modulating FTO-mediated N6-methyladenosine modification on GLUT1 and PKM2
Source: J Exp Clin Cancer Res. 2023 Oct 16;42:267. doi: 10.1186/s13046-023-02847-2 (PMC10578010; doi:10.1186/s13046-023-02847-2)
Supplement: Supplementary file 2 — Additional file 2. Supplementary methods. [file 13046_2023_2847_MOESM2_ESM.docx]

**LncRNA FTO-IT1 promotes glycolysis and progression of hepatocellular carcinoma through modulating FTO-mediated N6-methyladenosine modification on GLUT1 and PKM2**

**Supplementary Methods**

**Cell culture**

The human HCC cell lines HepG2, Hep3B and SNU449 were obtained from the American Typical Culture Center (ATCC, Manassas, USA), and human HCC cell lines MHCC97H, Huh7, Li7, PLC/PRF/5 and human normal liver cell lines MIHA were purchased from BeNa Culture Collection (BNCC, Beijing, China). They were tested and authenticated for genotypes by DNA fingerprinting within 6 months. Cells were cultured in RPMI-1640 (Gibco, Carlsbad, USA) or DMEM (Gibco) containing 10% fetal bovine serum (Gibco) and 100 U/mL penicillin and 100 mg/mL streptomycin at 37 °C under 5% CO_2_. Some specific experiments were carried out with cells in exponential growth, cultured in acidic (pH 6.8) or normal (pH 7.4) medium for 24 or 48 h. To achieve media acidification, 25 mM HEPES and PIPES (Sigma-Aldrich, Saint Louis, USA) were utilized. The pH of the media was measured by a high-precision pH meter. In some specific experiments, cells were cultured in low glucose medium or treated with CoCl_2_ (400 μM; Sigma-Aldrich) to simulate low glucose or hypoxia microenvironment. All cultures were monitored routinely and found to be free of contamination by mycoplasma or fungi.

**RNA isolation, reverse transcription, and quantitative real-time PCR**

Total RNA was extracted from cells and tissues by using Trizol RNAiso Plus (TaKaRa, Beijing, China). The reverse transcription was performed by the PrimeScript® RT Master Mix Perfect Real-Time (TaKaRa) based on provided directions. Reaction procedures of reverse transcriptions were as follows: 37 ℃ for 15 min; 85 ℃ for 5 s; 4 ℃ for 10 min. Quantitative real-time PCR (qPCR) was performed with the SYBR®Premix Ex Taq™ II (TaKaRa) and was conducted using StepOne-Plus System (Applied Biosystems, Waltham, USA). β-actin was used as loading control. Transcript levels were analyzed using the 2^-ΔΔCT^ method. Each sample was done in triplicate. All primer sequences used were shown in Supplementary Table S1.

**Transfection**

FTO-IT1 siRNA, FTO siRNA, ILF3 siRNA, ILF2 siRNA, YTHDF2 siRNA, c-Myc siRNA and negative controls siRNA were provided by GenePharma (Shanghai, China) and transfected with a final concentration of 50 nM. FTO-IT1, FTO, ILF3, ILF2, YTHDF2, c-Myc overexpression and empty vector plasmid were purchased from GeneChem (Shanghai, China) and transfected with 1.6 μg per 12 well plates. For stable transfection, overexpression plasmids were constructed into lentiviral vector and empty lentiviral vectors were as negative control. Lipofectamine 2000 (Invitrogen, Carlsbad, USA) and Opti-MEM (Gibco) was used for cell transfection refer to the manufacturer’s instructions. The transfection efficiency was identified via observing fluorescence intensity. Cells were cultured at normal medium or acidic medium for additional 48 h after transfection until collected for further assay. The sequences of siRNAs are shown in Supplementary Table S2.

**Cell proliferation assay**

MTT analysis was performed to observe the cell proliferation ability. The treated cells were plated at 2000 per well in 96-well plates and incubated for 1 to 5 days. 20 μL of MTT solution (0.5 mg/mL; Beyotime) was added to the medium at 37 ℃ for 4 h, and then the media was replaced by 150 μL of dimethyl sulfoxide (Sigma-Aldrich). Absorbance at 570 nm was then measured using microplate reader. There were 5 replicates per group, and the experiment was conducted independently three times.

For colony formation assay, the prepared cells were seeded into 6-well plates at a density of 2 000 cells with fresh cell culture medium and maintain the related drug concentration for 7 days. Then, the plates were washed with PBS, fixed with 4% paraformaldehyde fixing solution, and stained with 0.1% crystal violet (1 mg/mL; Beyotime) for 20 min. The colony pictures were captured using Nikon (Tokyo, Japan).

EdU cell proliferation assay was performed with BeyoClick™ EdU Cell Proliferation Kit with Alexa Fluor 555 (Beyotime) following the manufacturer’s protocol. Briefly, the cells were added with 10 μM EdU working solution and incubated at 37 ℃ for 2 h. After marking, cells were fixed by fixative and then washed three times. Subsequently, cells were treated using click additive solution, and were stained by Hoechst 33342. Stained results were observed by using LSM 5 Pascal Laser Scanning Microscope (Carl Zeiss, Oberkochen, Germany).

**Cell migration and invasion assays**

Wound healing assay was used to detect cell migration, and transwell assay was used to detect cell invasion. Please refer to our previous article for specific methods[1].

**Cell apoptosis assay**

The cells were washed with PBS 24h after transfection, centrifuged, and resuspended in PBS. After a second centrifugation, the cell pellet was resuspended in combined buffer solution. Annexin V-FITC and PI were added and incubated for 15min at 37°C. The stained cells were analyzed by flow cytometry to calculate the apoptosis rate using the formula: % apoptosis = (early + late apoptotic cells)/total cells x 100%.

**RNA fluorescence *in situ* hybridization (RNA-FISH)**

FISH analysis was conducted using the FISH Tag™ RNA Multicolour (Invitrogen) and MAXIscript^®^ (Ambion, Austin, USA) kits. The protocol was carried out as described by the manufacturers. In the brief, total RNA was extracted from the HCC cells using TRIzol reagent (TaKaRa). RNAs were reverse transcribed using primers containing a T7 promoter sequence specific for antisense of FTO-IT1. Then, the probes for FTO-IT1 were transcribed in vitro with the T7 RNA polymerase and labeled with red fluorescence. Finally, labeled probes were hybridized overnight with HCC cell samples at 55 ℃ in a humid environment. Stained results were observed by using LSM 5 Pascal Laser Scanning Microscope (Carl Zeiss). Probe sequences are given in Supplementary Table S1.

**Western blot**

Treated cells were lysed with lysis buffer combined with protease inhibitors to extract total protein. The prepared protein samples were electrophoretic in SDS-PAGE gel, and were then transferred to NC transfer membranes (Millipore, Boston, USA). After blocking, respective primary antibodies were incubated with protein bands at proper temperature and time, and secondary antibodies were incubated subsequently. Protein bands were washed ultimately, and band signals were visualized using ECL reagent (Servicebio, Wuhan, China) as well as collected by ChemiDocTm XRS Molecular Imager System (Bio-Rad, Hercules, USA). β-actin was used as a negative control.

**RNA stability assay**

For RNA stability assay, the prepared cells were seeded in 12-well plates, and then cells were treated with actinomycin D (MedChemExpress, New Jersey, USA) for indicated times at a final concentration of 5 μg/mL. Total RNA was then isolated, and real-time PCR was conducted to quantify the relative level of mRNA. 18S rRNA was used as a negative control for PCR.

**Co-immunoprecipitation (Co-IP)**

Cell lysates were prepared using Cell lysis buffer for IP (Beyotime) and adjusted to a final concentration of 1 μg/μL. Protein A agarose beads were removed to the diluted cell supernatant and agitated for 1 h at 4 ℃ to reduce the nonspecific background. Subsequently, cellular extracts were mixed overnight at 4 ℃ on a rotating platform with control IgG or primary antibodies (4 μg/mL). Protein A/G PLUS-Agarose (Beyotime) was then added and the mixture was incubated for a further 2 h at 4 ℃ with agitation. The agarose was then separated, rinsed with lysis buffer and equivalent volumes of each sample were analyzed by Western blot.

**Chromatin immunoprecipitation (ChIP)**

ChIP assays were performed by using EZ-ChIP™ Chromatin Immunoprecipitation Kit (Millipore). For each ChIP assay, 1 × 10^6^ cells were fixed in 1% formaldehyde for 10 min at 37 ℃ and washed twice using ice cold PBS containing protease inhibitors. Subsequently, cells were scraped into conical tube and lysed with SDS lysis buffer supplemented with protease inhibitors. The chromatin DNA was sonicated and sheared to lengths between 200 and 1000 bp. After centrifugation, the supernatant was transferred to a new tube and diluted with ChIP dilution buffer containing protease inhibitors. Protein A agarose beads were removed to the diluted cell supernatant and agitated for 1 h at 4 ℃ to reduce the nonspecific background. The supernatant fraction was collected, and mixed with anti-c-Myc antibody incubated overnight at 4 ℃ with rotation. Normal mouse IgG was served as a control, and an anti-RNA pol II antibody was used as the positive control. Protein A agarose beads were added to the reaction mixture for 1 h at 4 ℃ with rotation to collect the antibody/histone complex. Supernatant contained unbound and non-specific DNA was removed after gentle centrifugation. Protein A agarose/antibody/histone complex was washed on a rotating platform with the low salt immune complex wash buffer, high salt immune complex wash buffer, LiCl immune complex wash buffer and TE buffer. Elution buffer was used to separate the complex from the antibody. DNA samples were recovered by phenol/chloroform extraction and ethanol precipitation, and then amplified by PCR and separated by 2% agarose gel electrophoresis for analysis. The ChIP-qPCR primer sequences are given in Supplementary Table S1.

**Luciferase activity assay**

Wild type FTO promoter, FTO-IT1 promoter or mutant FTO-IT1 promoter (MUT1, MUT2, MUT3) were constructed into pGL3-based vectors, and transfected in HCC cells. The transfected cells were co-transfected with c-Myc siRNA or c-Myc overexpression plasmid (0.2 μg) and corresponding negative control respectively in 96-well plate. The luciferase activities were measured using dual luciferase reporter system (Promega, Madison, USA). Briefly, the prepared samples were incubated with firefly luciferase detection reagent or renilla luciferase detection reagent for 10 min at room temperature. Fluorescence signal and intensity were measured using fluorescence microplate reader. Fireﬂy luciferase activities were normalized by renilla luciferase activities. All experiments repeated three times independently and there were five samples per group.

**Immunohistochemistry (IHC)**

HCC tissues and adjacent noncancerous (NT) tissues were collected from patients with HCC. The xenograft tissues were dissected from euthanized mice. The tissues were fixed in 10% neutral buffered formalin solution overnight and then imbedded using paraffins. Briefly, paraffin sections were placed at 60 ℃ for 2 h, and washed successively in dimethylbenzene solution, ethanol, and TBS. Citrate buffer was utilized for antigen retrieval, while hydrogen peroxide was used to block endogenous peroxidase. Primary antibodies were used to incubate with tissues overnight at 4 ℃. HRP-labeled goat anti-mouse/rabbit IgG (H+L) was added on paraffin section for secondary antibody binding reaction. Diaminobenzidine was used for visualization, and hematoxylin was used for re-staining nucleus. Samples were washed again in dimethylbenzene solution and ethanol, and sealed with neutral balsam. Immunohistochemical staining results were examined under a light microscope.

**Reference**

1. Hu Y, Wang F, Xu F, Fang K, Fang Z, Shuai X, et al. A reciprocal feedback of Myc and lncRNA MTSS1-AS contributes to extracellular acidity-promoted metastasis of pancreatic cancer. Theranostics. 2020; 10: 10120-40.
