# Supplementary material for: LncRNA FTO-IT1 promotes glycolysis and progression of hepatocellular carcinoma through modulating FTO-mediated N6-methyladenosine modification on GLUT1 and PKM2
Source: J Exp Clin Cancer Res. 2023 Oct 16;42:267. doi: 10.1186/s13046-023-02847-2 (PMC10578010; doi:10.1186/s13046-023-02847-2)
Supplement: Supplementary file 3 — Additional file 3: Supplementary Table S1. The sequence of PCR primers. Supplementary Table S2. The sequence of siRNA. Supplementary Table S3. The information of antibodies used. Supplementary Table S4. Mass spectrometry data of proteins and peptides pulled down by FTO-IT1. Supplementary Table S5. The 63 glycolysis-related genes. Supplementary Table S6. Three putative c-Myc binding sites of FTO-IT1 promoter using JASPAR database. Supplementary Table S7. Clinical information and relative expression of mRNA of 92 patients with HCC. Supplementary Table S8. Correlation between FTO-IT1 and clinical characteristics of 92 patients with HCC. [file 13046_2023_2847_MOESM3_ESM.docx]

**LncRNA FTO-IT1 promotes glycolysis and progression of hepatocellular carcinoma through modulating FTO-mediated N6-methyladenosine modification on GLUT1 and PKM2**

**Supplementary Tables**

**Supplementary Table S1. The sequence of PCR primers.**

| Primers | Sequences (5’- 3’) | |
| --- | --- | --- |
| FTO-IT1 | Forward | GTCAAGGCCAAGTGTTGTGAG |
|  | Reverse | AATCCAGCTTCGGAGAGGAG |
| FTO | Forward | GCTGCTTATTTCGGGACCTG |
|  | Reverse | AGCCTGGATTACCAATGAGGA |
| ILF2 | Forward | GGGGAACAAAGTCGTGGAAAG |
|  | Reverse | CCAGTTTCGTTGGTCAGCA |
| ILF3 | Forward | GCCATTACGCCCATGAAACG |
|  | Reverse | AATGAATTGCCATCAACCTCCA |
| GLUT1 | Forward | TCTGGCATCAACGCTGTCTTC |
|  | Reverse | CGATACCGGAGCCAATGGT |
| PKM2 | Forward | ATGTCGAAGCCCCATAGTGAA |
|  | Reverse | TGGGTGGTGAATCAATGTCCA |
| ALDOA | Forward | ATGCCCTACCAATATCCAGCA |
|  | Reverse | GCTCCCAGTGGACTCATCTG |
| ENO1 | Forward | TACGTTCACCTCGGTGTCTG |
|  | Reverse | AACAGCCTTTGAGACACCCTT |
| PFKFB4 | Forward | TTTTTCTCCCCGACAATGAAGAG |
|  | Reverse | CACACAGATGGACTCGACAAA |
| PFKP | Forward | GACCTTCGTTCTGGAGGTGAT |
|  | Reverse | CACGGTTCTCCGAGAGTTTG |
| PGK1 | Forward | GACCTAATGTCCAAAGCTGAGAA |
|  | Reverse | CAGCAGGTATGCCAGAAGCC |
| c-Myc | Forward | GGCTCCTGGCAAAAGGTCA |
|  | Reverse | CTGCGTAGTTGTGCTGATGT |
| β-actin | Forward | ATGTGGCCGAGGACTTTGAT |
|  | Reverse | AGTGGGGTGGCTTTTAGGATG |
| 18S | Forward | GTAACCCGTTGAACCCCATT |
|  | Reverse | CCATCCAATCGGTAGTAGCG |
| FTO-IT1 probe for FISH | Forward | TAATACGACTCACACATTAAGCCCATTCCAGCCT |
|  | Reverse | AAGGAGCTACAAAGATGAACCA |
| FTO-IT1 *transcription in vitro* (for pull down) | Forward | TAATACGACTCACTATAGGGAGAGATCAAGAAGG |
|  | Reverse | CCTTTTTTTGGAGTGATCCTAATGG |
| anti-FTO-IT1 *transcription in vitro* (for pull down) | Forward | TAATACGACTCACTATAGGGTTTCAAAGCCAC |
|  | Reverse | TCCTGAGCAGACAGTGAAGAACTAGAAAGACA |
| ChIP for FTO-IT1 Site 1 | Forward | AAGGGACATGATTGTTTTGG |
|  | Reverse | ATACAGTGGCAGTTGGTTTG |
| ChIP for FTO-IT1 Site 2 | Forward | TCAGCAAACCAACTGCCACT |
|  | Reverse | ACATGAAAACTCAAAACCCA |
| ChIP for FTO-IT1 Site 3 | Forward | TAAAGCAAGACAGGGACACA |
|  | Reverse | CCTCATCAAAGTTCACAAAA |
| 3’UTR region of FTO (for RIP) | Forward | GTGGAGACTTCTCTTGGCCC |
|  | Reverse | GGGCACCATTTCCTAGCTGT |
| RP6-65G23.3 | Forward | AGACTTCTTTTGCCTGTTGG |
|  | Reverse | CTTGTGATTGGATCTGTTTGAG |
| RP11-932O9.10 | Forward | TTGAGAAGGCTTATGGTATG |
|  | Reverse | TAGAAGTGGCTTTGAATGGA |
| RP11-57A1.1 | Forward | AAAGAGATCCCCTCCCACCC |
|  | Reverse | CTGCTTAAAACCCCACAGCC |
| RP11-421M1.8 | Forward | AAGGAAGGTGGAGATGGATG |
|  | Reverse | GTAGGCTTTAGGTAGCAGGC |
| LL22NC03-N64E9.1 | Forward | CACTCTGATGTGAAAAAGGC |
|  | Reverse | AGATGTACTGCTCCAGCTAC |
| LL22NC03-N14H11.1 | Forward | GAATTTGATTGCAGTTCCTTCC |
|  | Reverse | CCCCCATAAACCACTCCCTA |

**Supplementary Table S2. The sequence of siRNA.**

| SiRNA Targets | Sequences (5’- 3’) | |
| --- | --- | --- |
| FTO-IT1#1 | sense | GGAUGGAGACUACUACUAATT |
|  | antisense | UUAGUAGUAGUCUCCAUCCTT |
| FTO-IT1#2 | sense | GCUGGAUUUAGCAGAGAUUTT |
|  | antisense | AAUCUCUGCUAAAUCCAGCTT |
| FTO#1 | sense | GCUGUGCUUCAUGAAGUUATT |
|  | antisense | UAACUUCAUGAAGCACAGCTT |
| FTO#2 | sense | GCACAAGCAUGGCUGCUUATT |
|  | antisense | UAAGCAGCCAUGCUUGUGCTT |
| ILF2#1 | sense | GAACUCCAUUUGGAUAUCATT |
|  | antisense | UGAUAUCCAAAUGGAGUUCTT |
| ILF2#2 | sense | GGCAACUUUAGAGUACACATT |
|  | antisense | UGUGUACUCUAAAGUUGCCTT |
| ILF3#1 | sense | GGAGGUUGAUGGCAAUUCATT |
|  | antisense | UGAAUUGCCAUCAACCUCCTT |
| ILF3#2 | sense | CCUCAUACCAAGGCAAACATT |
|  | antisense | UGUUUGCCUUGGUAUGAGGTT |
| YTHDF2#1 | sense | GCCCAAUAAUGCAUAUACUTT |
|  | antisense | AGUAUAUGCAUUAUUGGGCTT |
| YTHDF2#2 | sense | GCUCUGGAUAUAGUAGCAATT |
|  | antisense | UUGCUACUAUAUCCAGAGCTT |
| c-Myc#1 | sense | GAGGAUAUCUGGAAGAAAUTT |
|  | antisense | AUUUCUUCCAGAUAUCCUCTT |
| c-Myc#2 | sense | GCUUGUACCUGCAGGAUCUTT |
|  | antisense | AGAUCCUGCAGGUACAAGCTT |
| Negative control (NC) | Forward | UUCUCCGAACGUGUCACGUTT |
|  | Reverse | ACGUGACACGUUCGGAGAATT |

**Supplementary Table S3. The information of antibodies used.**

| Antibody | Experiment | Dilution | Supplier | Catalog no |
| --- | --- | --- | --- | --- |
| FTO | Western blot | 1:1000 | Proteintech, Wuhan, China | 27226-1-AP |
|  | IHC | 1:400 |  |  |
| ILF2 | Western blot | 1:500 | Proteintech, Wuhan, China | 14714-1-AP |
|  | RIP | 5 μg |  |  |
|  | Co-IP | 4 μg |  |  |
| ILF3 | Western blot | 1:2000 | Proteintech, Wuhan, China | 19887-1-AP |
|  | RIP | 5 μg |  |  |
|  | Co-IP | 4 μg |  |  |
| GLUT1 | Western blot | 1:1000 | Proteintech, Wuhan, China | 66290-1-Ig |
|  | IHC | 1:500 |  |  |
| PKM2 | Western blot | 1:1000 | Cell Signaling Technology, Boston, USA | 4053 |
|  | IHC | 1:200 |  |  |
| YTHDF2 | Western blot | 1:2000 | Proteintech, Wuhan, China | 24744-1-AP |
|  | RIP | 4 μg |  |  |
| c-Myc | Western blot | 1:2000 | Proteintech, Wuhan, China | 10828-1-AP |
|  | ChIP | 4 μg |  |  |
|  | IHC | 1:400 |  |  |
| ALDOA | Western blot | 1:4000 | Proteintech, Wuhan, China | 11217-1-AP |
| ENO1 | Western blot | 1:2000 | Proteintech, Wuhan, China | 11204-1-AP |
| PFKFB4 | Western blot | 1:1000 | Abcam, Cambridge, UK | ab137785 |
| PFKP | Western blot | 1:1000 | ABclonal, Wuhan, China | A21538 |
| PGK1 | Western blot | 1:5000 | Proteintech, Wuhan, China | 17811-1-AP |
| Ki67 | IHC | 1:100 | ABclonal, Wuhan, China | A20018 |
| β-actin | Western blot | 1:1000 | Proteintech, Wuhan, China | 20536-1-AP |
| m^6^A | MeRIP | 10 μg | Abcam, Cambridge, UK | ab151230 |
| HRP-labeled goat anti-mouse IgG (H+L) | Western blot | 1:3000 | Cell Signaling Technology, Boston, USA | 14709 |
| HRP-labeled goat anti-rabbit IgG (H+L) | Western blot | 1:3000 | Cell Signaling Technology, Boston, USA | 14708 |

**Supplementary Table S4. Mass spectrometry data of proteins and peptides pulled down by FTO-IT1.**

| **Accession** | **Description** | **Score SENSE** | **Coverage SENSE** | **# Peptides SENSE** | **# PSM SENSE** | **Score ANTISENSE** | **Coverage ANTISENSE** | **# Peptides ANTISENSE** | **# PSM ANTISENSE** | **# AAs** | **MW [kDa]** |
| --- | --- | --- | --- | --- | --- | --- | --- | --- | --- | --- | --- |
| A0A1S5UZ39 | Hemoglobin subunit alpha | **-** | **-** | **-** | **-** | 44.73 | 42.78 | 3 | 3 | 187 | 20.1 |
| P13645 | Keratin, type I cytoskeletal 10 | 600.68 | 32.19 | 12 | 13 | 630.95 | 31.16 | 11 | 13 | 584 | 58.8 |
| P35527 | Keratin, type I cytoskeletal 9 | 436.09 | 21.67 | 8 | 9 | 535.13 | 32.58 | 11 | 12 | 623 | 62.0 |
| H6VRF8 | Keratin 1 | 385.48 | 19.25 | 8 | 8 | 574.29 | 29.19 | 16 | 19 | 644 | 66.0 |
| P35908 | Keratin, type II cytoskeletal 2 epidermal | 194.29 | 14.55 | 6 | 6 | 386.87 | 24.10 | 11 | 11 | 639 | 65.4 |
| V9GZN0 | Uncharacterized protein (Fragment) | 51.72 | 19.15 | 1 | 1 | 46.61 | 19.15 | 1 | 1 | 47 | 5.0 |
| Q96DE1 | Uncharacterized protein (Fragment) | 65.04 | 10.06 | 1 | 1 | 63.82 | 8.18 | 1 | 1 | 159 | 17.7 |
| F8W6I7 | Heterogeneous nuclear ribonucleoprotein A1 | 147.10 | 15.96 | 3 | 3 | **-** | **-** | **-** | **-** | 307 | 33.1 |
| H7C0I5 | Uncharacterized protein (Fragment) | 32.41 | 15.71 | 1 | 1 | **-** | **-** | **-** | **-** | 140 | 15.4 |
| Q86W20 | Protease serine 1 (Fragment) | **-** | **-** | **-** | **-** | 30.98 | 15.48 | 1 | 1 | 84 | 9.2 |
| A0A024R1X6 | Keratin 14 | 101.44 | 12.64 | 3 | 3 | 69.75 | 4.98 | 1 | 1 | 261 | 30.0 |
| H0YEW6 | Myomegalin (Fragment) | 37.83 | 10.34 | 1 | 1 | **-** | **-** | **-** | **-** | 87 | 10.0 |
| H0YK49 | Electron transfer flavoprotein subunit alpha, mitochondrial | 42.62 | 8.30 | 1 | 1 | 51.10 | 8.30 | 1 | 1 | 229 | 24.1 |
| Q86U12 | Full-length cDNA clone CS0CAP007YF18 of Thymus of Homo sapiens | 0.00 | 3.39 | 1 | 1 | 41.56 | 2.42 | 1 | 1 | 413 | 49.3 |
| **B4DFG4** | **cDNA FLJ58801, highly similar to Interleukin enhancer-binding factor 3** | **55.85** | **4.94** | **2** | **2** | **-** | **-** | **-** | **-** | **506** | **54.7** |
| A0A087WWT3 | Serum albumin | **-** | **-** | **-** | **-** | 81.65 | 3.79 | 1 | 2 | 396 | 45.1 |
| B4DUH1 | cDNA FLJ51323, highly similar to Short-chain specific acyl-CoA dehydrogenase, mitochondrial | **-** | **-** | **-** | **-** | 48.72 | 3.21 | 1 | 1 | 405 | 43.6 |
| A0A384MDW7 | Enoyl Coenzyme A hydratase, short chain, 1, mitochondrial | 32.62 | 3.10 | 1 | 1 | **-** | **-** | **-** | **-** | 290 | 31.4 |
| B3KU66 | cDNA FLJ39263 fis, clone OCBBF2009571, highly similar to ATP-dependent RNA helicase A | 61.16 | 2.91 | 1 | 1 | **-** | **-** | **-** | **-** | 549 | 59.9 |
| **B4DY09** | **Interleukin enhancer-binding factor 2** | **68.40** | **2.84** | **1** | **1** | **-** | **-** | **-** | **-** | **352** | **38.9** |
| Q9H072 | Uncharacterized protein DKFZp586J151 | 30.82 | 2.44 | 1 | 1 | **-** | **-** | **-** | **-** | 533 | 58.1 |
| P25705 | ATP synthase subunit alpha, mitochondrial | 61.04 | 2.35 | 1 | 4 | 62.82 | 2.35 | 1 | 3 | 553 | 59.7 |
| P11498 | Pyruvate carboxylase, mitochondrial | 29.68 | 0.85 | 1 | 1 | **-** | **-** | **-** | **-** | 1178 | 129.6 |

Note: ILF2 and ILF3 were in bold.

**Supplementary Table S5. The 63 glycolysis-related genes.**

| **Classification** | **Genes** |  |  |  |  |
| --- | --- | --- | --- | --- | --- |
| **Glucose Transporter (GLUTs)** | SLC2A1 | SLC2A2 | SLC2A3 | SLC2A4 | SLC2A5 |
|  | SLC2A6 | SLC2A7 | SLC2A8 | SLC2A9 | SLC2A10 |
|  | SLC2A11 | SLC2A12 | SLC2A14 |  |  |
| **Glycolysis** | ADPGK |  |  |  |  |
|  | ALDOA | ALDOB | ALDOC |  |  |
|  | BPGM |  |  |  |  |
|  | ENO1 | ENO2 | ENO3 | ENO4 |  |
|  | GAPDH | GADPHS |  |  |  |
|  | GCK |  |  |  |  |
|  | GPI |  |  |  |  |
|  | HK1 | HK2 | HK3 |  |  |
|  | HKDC1 |  |  |  |  |
|  | PFKFB1 | PFKFB2 | PFKFB3 | PFKFB4 |  |
|  | PFKL | PFKM | PFKP |  |  |
|  | PGAM1 | PGAM2 | PGAM4 |  |  |
|  | PGK1 | PGK2 |  |  |  |
|  | PKL/R | PKM1/2 |  |  |  |
|  | TPI1 |  |  |  |  |
|  | LDHA | LDHB | LDHC |  |  |
|  | LDHAL6A | LDHAL6B |  |  |  |
|  | UEVLD |  |  |  |  |
| **Monocarboxylic Acid Transporter (MCTs)** | SLC16A1 | SLC16A2 | SLC16A3 | SLC16A4 | SLC16A5 |
|  | SLC16A6 | SLC16A7 | SLC16A8 | SLC16A9 | SLC16A10 |
|  | SLC16A11 | SLC16A12 |  |  |  |

**Supplementary Table S6. Three putative c-Myc binding sites of FTO-IT1 promoter using JASPAR database.**

| **Matrix ID** | **Name** | **Score** | **Sequence ID** | **Start** | **End** | **Predicted sequence** | **Corresponding site** |
| --- | --- | --- | --- | --- | --- | --- | --- |
| MA0147.3 | MYC | 13.2192 | NC_000016.10:54036393-54039393 | -2360 | -2349 | GGCCACGTGACC | Site 2 |
| MA0147.3 | MYC | 8.45547 | NC_000016.10:54036393-54039393 | -2408 | -2397 | GTCCACTTGCTC | Site 1 |
| MA0147.3 | MYC | 6.4952 | NC_000016.10:54036393-54039393 | -251 | -240 | CTACATGTGCCA | Site 3 |

**Supplementary Table S7. Clinical information and relative expression of mRNA of 92 patients with HCC.**

| **ID** | **Age (year)** | **Gender** | **Duration of follow-up (month)** | **Status** | **Stage** | **Child-Pugh** | **AFP (μg/L)** | **Tumor size (cm)** | **Lymphatic invasion** | **Distant metastasis** | **Vascular invasion** | **Differentiation** | **Relative expression of mRNA** | | | | | | |
| --- | --- | --- | --- | --- | --- | --- | --- | --- | --- | --- | --- | --- | --- | --- | --- | --- | --- | --- | --- |
|  |  |  |  |  |  |  |  |  |  |  |  |  | **FTO-IT1 in HCC** | **FTO-IT1 in NT** | **FTO in HCC** | **FTO in NT** | **GLUT1 in HCC** | **PKM2 in HCC** | **c-Myc in HCC** |
| 1 | 63 | male | 22 | survival | Ⅱ | B | 818.49 | 7.9 | negative | negative | negative | high | 22.47 | 9.94 | 4.80 | 1.93 | 5.08 | 2.91 | 1.94 |
| 2 | 52 | female | 19 | death | Ⅰ | A | 494.14 | 4.0 | negative | negative | negative | moderate | 20.22 | 7.57 | 1.96 | 2.61 | 5.11 | 2.98 | 3.28 |
| 3 | 78 | male | 20 | survival | Ⅲ | A | 886.25 | 5.4 | positive | negative | positive | moderate | 27.29 | 8.73 | 7.56 | 2.29 | 5.80 | 4.01 | 7.76 |
| 4 | 79 | male | 17 | death | Ⅲ | A | 2198.35 | 6.8 | positive | negative | positive | moderate | 20.55 | 4.23 | 7.09 | 2.61 | 5.83 | 4.35 | 7.66 |
| 5 | 69 | female | 24 | death | Ⅰ | B | 18.04 | 9.8 | negative | negative | negative | moderate | 32.57 | 9.34 | 6.38 | 1.00 | 5.94 | 4.43 | 5.66 |
| 6 | 46 | male | 13 | death | Ⅰ | B | 135.52 | 9.6 | negative | negative | negative | high | 20.14 | 10.89 | 8.08 | 2.61 | 5.96 | 4.60 | 5.75 |
| 7 | 70 | female | 28 | death | Ⅲ | A | 5.68 | 9.3 | negative | positive | negative | low | 32.45 | 12.47 | 10.36 | 4.89 | 5.99 | 4.89 | 5.76 |
| 8 | 60 | female | 27 | death | Ⅲ | B | 918.88 | 10 | negative | positive | negative | low | 21.53 | 3.97 | 8.39 | 2.31 | 6.09 | 4.50 | 7.18 |
| 9 | 79 | male | 25 | death | Ⅱ | A | 820.78 | 5.6 | negative | negative | negative | low | 23.76 | 3.54 | 5.35 | 1.20 | 5.06 | 2.52 | 4.01 |
| 10 | 64 | male | 17 | death | Ⅲ | B | 474.13 | 5.1 | negative | negative | positive | low | 39.69 | 7.26 | 5.47 | 3.01 | 4.88 | 3.25 | 3.39 |
| 11 | 47 | male | 13 | death | Ⅲ | B | 202.19 | 4.9 | positive | negative | positive | moderate | 22.81 | 8.25 | 3.04 | 2.11 | 4.94 | 2.33 | 1.01 |
| 12 | 54 | male | 17 | survival | Ⅰ | A | 726.79 | 4.2 | negative | negative | negative | moderate | 20.40 | 12.55 | 3.04 | 3.02 | 4.97 | 2.51 | 2.80 |
| 13 | 56 | female | 27 | survival | Ⅱ | A | 543.59 | 4.7 | positive | negative | negative | high | 4.63 | 3.75 | 6.83 | 2.71 | 1.00 | 2.61 | 4.51 |
| 14 | 66 | male | 8 | death | Ⅳ | C | 690.26 | 11.2 | positive | positive | positive | low | 19.96 | 9.34 | 1.21 | 3.41 | 5.77 | 4.04 | 5.02 |
| 15 | 64 | male | 16 | survival | Ⅱ | A | 441.5 | 7.4 | negative | negative | negative | high | 21.66 | 4.10 | 4.82 | 3.61 | 7.55 | 3.80 | 5.48 |
| 16 | 73 | male | 40 | death | Ⅱ | A | 1673.8 | 3.9 | positive | negative | negative | moderate | 32.84 | 11.79 | 3.31 | 2.92 | 7.66 | 3.19 | 5.38 |
| 17 | 62 | female | 20 | death | Ⅲ | A | 502.61 | 6.5 | positive | negative | positive | moderate | 22.78 | 10.04 | 3.67 | 1.16 | 7.68 | 3.57 | 7.44 |
| 18 | 76 | male | 20 | survival | Ⅱ | A | 552.11 | 10.2 | negative | negative | negative | moderate | 19.96 | 6.33 | 5.27 | 2.61 | 4.80 | 3.17 | 4.64 |
| 19 | 46 | female | 21 | death | Ⅲ | A | 486.22 | 6.2 | negative | negative | positive | low | 26.82 | 9.95 | 5.32 | 6.54 | 4.80 | 4.58 | 1.11 |
| 20 | 58 | male | 18 | survival | Ⅰ | A | 805.59 | 3.9 | negative | negative | negative | high | 5.30 | 4.10 | 3.54 | 2.40 | 1.20 | 1.82 | 1.10 |
| 21 | 72 | male | 27 | survival | Ⅲ | B | 12.04 | 8.0 | positive | negative | positive | low | 10.00 | 10.13 | 4.72 | 2.43 | 3.15 | 2.26 | 3.20 |
| 22 | 70 | female | 17 | survival | Ⅰ | A | 430.5 | 6.6 | negative | negative | negative | high | 6.46 | 9.03 | 2.79 | 3.77 | 3.82 | 2.60 | 2.55 |
| 23 | 63 | male | 24 | death | Ⅲ | A | 1417.99 | 4.1 | positive | negative | positive | moderate | 29.17 | 4.27 | 6.27 | 2.23 | 6.15 | 4.69 | 5.82 |
| 24 | 66 | male | 23 | death | Ⅳ | C | 3240.9 | 6.8 | positive | negative | positive | low | 21.77 | 8.52 | 7.25 | 2.83 | 6.19 | 3.50 | 5.63 |
| 25 | 60 | male | 15 | survival | Ⅲ | A | 302.93 | 5.3 | positive | negative | positive | low | 25.80 | 2.44 | 6.64 | 2.69 | 6.20 | 3.73 | 5.46 |
| 26 | 67 | female | 10 | death | Ⅱ | A | 300.68 | 11.2 | positive | negative | negative | low | 27.29 | 10.42 | 9.12 | 4.34 | 6.22 | 4.47 | 5.48 |
| 27 | 72 | female | 17 | survival | Ⅲ | A | 271.8 | 4.9 | positive | negative | positive | moderate | 32.57 | 10.87 | 8.41 | 1.06 | 6.29 | 4.61 | 6.02 |
| 28 | 73 | male | 13 | death | Ⅳ | C | 1265.83 | 3.9 | positive | negative | positive | low | 20.14 | 5.37 | 8.06 | 4.33 | 6.37 | 4.88 | 5.09 |
| 29 | 78 | female | 21 | death | Ⅱ | B | 456.39 | 4.9 | positive | negative | negative | moderate | 32.45 | 1.82 | 7.66 | 4.94 | 6.49 | 4.94 | 7.19 |
| 30 | 61 | male | 12 | survival | Ⅰ | A | 1.47 | 3.3 | negative | negative | negative | high | 10.57 | 9.71 | 3.59 | 4.14 | 5.44 | 2.87 | 1.81 |
| 31 | 60 | male | 15 | survival | Ⅱ | A | 2.63 | 5.8 | negative | negative | negative | moderate | 9.53 | 20.79 | 4.57 | 3.73 | 1.13 | 1.59 | 3.91 |
| 32 | 47 | male | 30 | death | Ⅱ | A | 4.62 | 5.4 | positive | negative | negative | moderate | 7.06 | 6.41 | 5.65 | 2.84 | 1.19 | 1.82 | 3.78 |
| 33 | 69 | male | 17 | survival | Ⅱ | A | 15.44 | 3.3 | positive | negative | negative | moderate | 22.18 | 6.45 | 4.17 | 3.24 | 5.16 | 3.02 | 3.28 |
| 34 | 67 | male | 24 | death | Ⅱ | A | 473.18 | 4.5 | negative | negative | negative | moderate | 14.66 | 9.95 | 6.79 | 2.80 | 2.15 | 2.58 | 1.61 |
| 35 | 75 | female | 22 | death | Ⅰ | A | 457.59 | 6.0 | negative | negative | negative | high | 10.77 | 7.88 | 7.81 | 1.71 | 2.30 | 1.29 | 1.80 |
| 36 | 81 | male | 36 | survival | Ⅱ | A | 422.91 | 3.1 | negative | negative | negative | moderate | 17.02 | 1.61 | 7.93 | 3.04 | 4.63 | 3.19 | 1.70 |
| 37 | 77 | female | 25 | survival | Ⅱ | A | 384.73 | 3.7 | negative | negative | negative | moderate | 10.77 | 14.95 | 5.65 | 1.82 | 5.34 | 2.77 | 4.27 |
| 38 | 61 | male | 17 | death | Ⅲ | A | 509.07 | 5.1 | positive | negative | positive | low | 14.06 | 8.81 | 5.27 | 1.82 | 5.43 | 2.02 | 1.46 |
| 39 | 57 | male | 15 | death | Ⅱ | A | 1131.51 | 6.7 | negative | negative | negative | moderate | 29.17 | 9.51 | 9.43 | 2.61 | 6.63 | 3.74 | 4.90 |
| 40 | 51 | female | 3 | death | Ⅱ | A | 874.35 | 7.7 | negative | negative | negative | high | 21.77 | 7.40 | 7.28 | 2.83 | 6.65 | 3.58 | 5.21 |
| 41 | 71 | male | 18 | death | Ⅳ | C | 1530.93 | 5.7 | positive | negative | negative | low | 23.48 | 3.09 | 7.71 | 2.10 | 6.83 | 4.46 | 6.45 |
| 42 | 48 | male | 23 | death | Ⅰ | A | 634.64 | 6.7 | negative | negative | negative | moderate | 4.63 | 2.01 | 10.04 | 4.67 | 4.65 | 4.09 | 1.00 |
| 43 | 55 | female | 22 | death | Ⅲ | A | 267.5 | 3.0 | negative | negative | positive | moderate | 15.35 | 4.94 | 8.57 | 4.96 | 4.67 | 4.07 | 5.71 |
| 44 | 60 | male | 30 | death | Ⅱ | A | 654.5 | 9.9 | negative | negative | negative | low | 10.77 | 2.27 | 6.58 | 1.88 | 5.53 | 3.86 | 5.27 |
| 45 | 48 | male | 17 | survival | Ⅱ | A | 224.12 | 4.9 | negative | negative | negative | moderate | 10.74 | 9.89 | 6.78 | 2.87 | 7.21 | 3.69 | 4.61 |
| 46 | 69 | female | 27 | survival | Ⅰ | A | 2.56 | 3.9 | negative | negative | negative | high | 12.50 | 5.20 | 5.35 | 4.66 | 5.17 | 2.97 | 1.27 |
| 47 | 61 | female | 26 | survival | Ⅱ | A | 411.22 | 4.7 | negative | negative | negative | high | 2.94 | 7.48 | 4.92 | 4.25 | 5.26 | 2.74 | 1.54 |
| 48 | 74 | male | 19 | survival | Ⅲ | A | 4.73 | 4.1 | positive | negative | positive | low | 16.16 | 1.76 | 3.84 | 1.90 | 5.28 | 1.39 | 2.14 |
| 49 | 57 | female | 24 | death | Ⅲ | A | 2456.55 | 6.7 | positive | negative | positive | moderate | 25.80 | 10.16 | 9.35 | 2.13 | 6.87 | 3.93 | 5.06 |
| 50 | 70 | male | 18 | death | Ⅲ | B | 2318.78 | 5.7 | positive | negative | positive | low | 23.26 | 8.31 | 6.25 | 2.61 | 6.90 | 3.27 | 5.32 |
| 51 | 71 | male | 20 | survival | Ⅱ | A | 829.63 | 6.5 | negative | negative | negative | high | 25.39 | 5.13 | 10.22 | 1.58 | 6.96 | 4.36 | 7.16 |
| 52 | 79 | male | 21 | survival | Ⅲ | B | 642.09 | 6.5 | positive | negative | positive | low | 22.48 | 11.65 | 8.61 | 3.22 | 6.99 | 1.83 | 6.29 |
| 53 | 55 | male | 25 | death | Ⅰ | A | 6.05 | 5.4 | negative | negative | negative | high | 27.43 | 6.78 | 8.86 | 2.61 | 7.00 | 1.84 | 5.71 |
| 54 | 66 | male | 20 | survival | Ⅲ | A | 765.86 | 8.1 | positive | negative | positive | low | 21.37 | 8.86 | 6.71 | 2.00 | 7.12 | 1.97 | 6.14 |
| 55 | 57 | male | 10 | death | Ⅳ | C | 2067.46 | 6.7 | positive | negative | positive | low | 23.68 | 1.00 | 6.79 | 3.12 | 7.15 | 2.16 | 5.29 |
| 56 | 64 | male | 25 | death | Ⅲ | B | 71.46 | 4.2 | positive | negative | positive | low | 8.21 | 9.65 | 2.41 | 3.41 | 4.68 | 1.91 | 2.79 |
| 57 | 72 | male | 24 | survival | Ⅱ | A | 271.03 | 3.8 | negative | negative | negative | moderate | 7.65 | 11.53 | 6.35 | 3.08 | 7.31 | 4.56 | 5.63 |
| 58 | 76 | female | 15 | survival | Ⅰ | B | 590.26 | 8.5 | negative | negative | negative | high | 6.10 | 8.63 | 7.07 | 2.42 | 9.69 | 4.72 | 7.77 |
| 59 | 70 | male | 24 | death | Ⅲ | B | 263.15 | 5.1 | positive | negative | positive | moderate | 17.55 | 5.18 | 7.15 | 2.71 | 9.79 | 3.42 | 6.07 |
| 60 | 68 | male | 18 | death | Ⅰ | A | 101.49 | 4.5 | negative | negative | negative | high | 16.85 | 17.72 | 3.84 | 3.04 | 4.47 | 1.70 | 3.16 |
| 61 | 49 | female | 21 | survival | Ⅰ | A | 45.52 | 3.8 | negative | negative | negative | high | 16.76 | 13.86 | 5.47 | 2.39 | 4.60 | 2.40 | 3.17 |
| 62 | 59 | male | 24 | death | Ⅲ | A | 529.15 | 4.2 | positive | negative | positive | low | 19.64 | 4.38 | 8.08 | 3.31 | 7.21 | 2.24 | 6.64 |
| 63 | 57 | male | 17 | survival | Ⅰ | A | 356.5 | 8.4 | negative | negative | negative | moderate | 30.19 | 6.44 | 9.84 | 4.07 | 3.53 | 2.68 | 5.89 |
| 64 | 74 | female | 10 | death | Ⅰ | A | 348.2 | 4.4 | negative | negative | negative | high | 35.26 | 9.11 | 7.41 | 4.47 | 3.93 | 2.41 | 6.00 |
| 65 | 47 | male | 16 | death | Ⅲ | A | 616.25 | 6.4 | positive | negative | positive | high | 44.07 | 4.64 | 8.31 | 3.12 | 4.09 | 2.21 | 5.36 |
| 66 | 50 | male | 18 | death | Ⅱ | B | 612.8 | 4.1 | negative | negative | negative | moderate | 29.84 | 4.70 | 7.68 | 4.51 | 4.10 | 1.00 | 6.37 |
| 67 | 38 | female | 9 | survival | Ⅲ | B | 547.01 | 6.1 | negative | negative | positive | high | 40.81 | 6.33 | 15.92 | 2.07 | 4.15 | 2.41 | 1.87 |
| 68 | 72 | female | 20 | death | Ⅱ | A | 506.21 | 5.8 | negative | negative | negative | high | 31.51 | 3.85 | 9.14 | 2.10 | 4.16 | 2.41 | 2.29 |
| 69 | 55 | male | 11 | survival | Ⅲ | A | 622.8 | 8.4 | positive | negative | positive | low | 22.18 | 14.47 | 7.68 | 2.55 | 4.29 | 2.65 | 3.79 |
| 70 | 63 | male | 26 | death | Ⅲ | A | 24.29 | 4.6 | negative | negative | positive | low | 10.17 | 9.21 | 5.80 | 4.75 | 4.78 | 2.63 | 2.39 |
| 71 | 59 | male | 19 | death | Ⅱ | A | 3.09 | 8.8 | negative | negative | negative | moderate | 6.15 | 3.00 | 2.86 | 2.39 | 5.63 | 3.17 | 6.23 |
| 72 | 66 | female | 22 | survival | Ⅲ | B | 170.65 | 5.4 | positive | negative | positive | moderate | 4.53 | 7.25 | 4.42 | 3.41 | 7.31 | 3.18 | 5.09 |
| 73 | 64 | male | 23 | death | Ⅱ | A | 297.73 | 3.3 | negative | negative | negative | high | 12.68 | 6.63 | 3.56 | 1.88 | 7.39 | 3.18 | 5.17 |
| 74 | 63 | male | 3 | survival | Ⅲ | A | 125.68 | 6.2 | positive | negative | positive | low | 11.12 | 7.17 | 3.56 | 2.01 | 8.57 | 3.14 | 6.29 |
| 75 | 44 | female | 24 | death | Ⅱ | A | 121.88 | 6.6 | negative | negative | negative | high | 15.58 | 8.48 | 5.67 | 2.76 | 8.66 | 1.97 | 5.71 |
| 76 | 65 | male | 30 | survival | Ⅰ | B | 479.64 | 3.6 | negative | negative | negative | high | 13.77 | 3.81 | 3.74 | 5.20 | 8.86 | 2.96 | 5.87 |
| 77 | 64 | female | 23 | death | Ⅲ | A | 676.6 | 8.1 | positive | negative | positive | low | 20.11 | 5.06 | 11.54 | 4.47 | 4.33 | 2.78 | 2.94 |
| 78 | 70 | male | 14 | death | Ⅳ | C | 1011.42 | 6.7 | positive | positive | positive | low | 24.40 | 4.39 | 6.47 | 2.02 | 4.35 | 2.86 | 3.98 |
| 79 | 78 | male | 22 | death | Ⅰ | A | 212.48 | 5.8 | negative | negative | negative | high | 27.08 | 4.35 | 6.50 | 3.10 | 4.38 | 2.99 | 2.51 |
| 80 | 57 | female | 2 | survival | Ⅰ | A | 211.58 | 5.3 | negative | negative | negative | high | 26.79 | 18.79 | 5.12 | 3.04 | 5.68 | 4.34 | 5.32 |
| 81 | 72 | female | 5 | survival | Ⅲ | A | 588.22 | 5.9 | negative | positive | positive | low | 26.85 | 5.19 | 5.22 | 3.12 | 5.69 | 4.58 | 5.00 |
| 82 | 72 | male | 18 | death | Ⅲ | A | 544.93 | 6.2 | negative | positive | positive | low | 31.05 | 4.03 | 6.13 | 3.75 | 5.73 | 3.39 | 5.39 |
| 83 | 67 | male | 11 | survival | Ⅱ | B | 169.55 | 4.0 | positive | negative | negative | moderate | 15.55 | 9.88 | 5.20 | 4.51 | 2.37 | 3.09 | 5.09 |
| 84 | 61 | male | 25 | death | Ⅲ | A | 1368.65 | 3.1 | negative | negative | positive | low | 19.27 | 6.85 | 4.29 | 2.05 | 2.42 | 2.42 | 3.77 |
| 85 | 43 | female | 22 | survival | Ⅱ | A | 591.72 | 7.4 | negative | negative | negative | moderate | 10.28 | 5.43 | 3.41 | 3.65 | 2.42 | 1.46 | 2.01 |
| 86 | 38 | female | 24 | death | Ⅲ | A | 183.2 | 3.4 | positive | negative | positive | low | 13.48 | 7.77 | 5.07 | 2.63 | 3.32 | 2.33 | 4.14 |
| 87 | 53 | male | 22 | survival | Ⅱ | A | 111.99 | 6.0 | negative | negative | negative | moderate | 13.80 | 6.85 | 6.10 | 3.95 | 4.45 | 2.03 | 3.85 |
| 88 | 47 | female | 10 | survival | Ⅲ | A | 1757.45 | 5.2 | positive | negative | positive | moderate | 7.60 | 9.13 | 5.15 | 1.77 | 7.74 | 3.66 | 5.43 |
| 89 | 72 | female | 22 | survival | Ⅲ | A | 3.86 | 4.8 | positive | negative | positive | moderate | 8.35 | 5.15 | 5.60 | 5.76 | 7.76 | 4.04 | 5.24 |
| 90 | 61 | male | 32 | survival | Ⅰ | A | 361.29 | 5.9 | negative | negative | negative | moderate | 18.52 | 13.05 | 5.75 | 2.61 | 8.07 | 4.18 | 7.26 |
| 91 | 69 | female | 48 | death | Ⅱ | A | 35.73 | 4.7 | negative | negative | negative | moderate | 11.06 | 3.42 | 3.49 | 2.61 | 8.07 | 3.65 | 6.04 |
| 92 | 57 | male | 4 | survival | Ⅲ | A | 1434.02 | 6.3 | negative | negative | positive | low | 10.54 | 13.37 | 5.07 | 1.60 | 8.41 | 3.13 | 4.89 |

Note: China liver cancer staging (CNLC, 2022 edition) was used for staging of these patients. HCC, hepatocellular carcinoma; NT: non-tumor.

**Supplementary Table S8. Correlation between FTO-IT1 and clinical characteristics of 92 patients with HCC.**

| **Characteristics** | **Number of cases** | **FTO-IT1 expression** | | ***P* value (Chi-Squared Test)** |
| --- | --- | --- | --- | --- |
|  |  | **High** | **Low** |  |
| Total cases | 92 | 50 | 42 | - |
| Gender |  |  |  |  |
| Male | 59 | 33 | 26 | 0.683 |
| Female | 33 | 17 | 16 |  |
| Age (year) |  |  |  |  |
| < 60 | 31 | 17 | 14 | 0.946 |
| ≥ 60 | 61 | 33 | 28 |  |
| AFP (μg/L) |  |  |  | **0.002** |
| < 25 | 13 | 4 | 9 |  |
| ≥ 25 and < 400 | 26 | 9 | 17 |  |
| ≥ 400 | 53 | 37 | 16 |  |
| Child-Pugh |  |  |  | **0.043** |
| A | 68 | 33 | 35 |  |
| B | 18 | 11 | 7 |  |
| C | 6 | 6 | 0 |  |
| Tumor size (cm) |  |  |  | **0.003** |
| < 5 | 35 | 12 | 23 |  |
| ≥ 5 | 57 | 38 | 19 |  |
| Lymphatic invasion |  |  |  | 0.065 |
| Positive | 38 | 25 | 13 |  |
| Negative | 54 | 25 | 29 |  |
| Distant metastasis |  |  |  | **0.020** |
| Positive | 6 | 6 | 0 |  |
| Negative | 86 | 44 | 42 |  |
| Vascular invasion |  |  |  | 0.107 |
| Positive | 39 | 25 | 14 |  |
| Negative | 53 | 25 | 28 |  |
| Differentiation |  |  |  | 0.184 |
| High | 25 | 12 | 13 |  |
| Moderate | 36 | 17 | 19 |  |
| Low | 31 | 21 | 10 |  |
| Stage * |  |  |  | **0.049** |
| I | 20 | 9 | 11 |  |
| II | 30 | 13 | 17 |  |
| III | 36 | 22 | 14 |  |
| IV | 6 | 6 | 0 |  |

Note: High expression of FTO-IT1 was significantly correlated with AFP, Child-Pugh grade, tumor size, distant metastasis and CNLC stage, but not with gender, age, lymphatic invasion, vascular invasion and differentiation grade (Statistically significant P value were in bold).

*: China liver cancer staging (CNLC, 2022 edition) was used for staging of these patients.
